# Analysis and comparison of the pan-genomic properties of sixteen well-characterized bacterial genera

**DOI:** 10.1186/1471-2180-10-258

**Published:** 2010-10-13

**Authors:** Brett Trost, Monique Haakensen, Vanessa Pittet, Barry Ziola, Anthony Kusalik

**Affiliations:** 1 Department of Computer Science, University of Saskatchewan, 176 Thorvaldson Building, 110 Science Place, Saskatoon, Saskatchewan, S7N 5C9, Canada; 2 Department of Pathology and Laboratory Medicine, University of Saskatchewan, 2841 Royal University Hospital, 103 Hospital Drive, Saskatoon, Saskatchewan, S7N 0W8, Canada; 3 Saskatchewan Research Council, 125-15 Innovation Boulevard, Saskatoon, Saskatchewan, S7N 2X8, Canada

## Abstract

**Background:**

The increasing availability of whole genome sequences allows the gene or protein content of different organisms to be compared, leading to burgeoning interest in the relatively new subfield of pan-genomics. However, while several studies have analyzed protein content relationships in specific groups of bacteria, there has yet to be a study that provides a general characterization of protein content relationships in a broad range of bacteria.

**Results:**

A variation on reciprocal BLAST hits was used to infer relationships among proteins in several groups of bacteria, and data regarding protein conservation and uniqueness in different bacterial genera are reported in terms of "core proteomes", "unique proteomes", and "singlets". We also analyzed the relationship between protein content similarity and the percent identity of the 16S rRNA gene in pairs of bacterial isolates from the same genus, and found that the strength of this relationship varied substantially depending on the genus, perhaps reflecting different rates of genome evolution and/or horizontal gene transfer. Finally, core proteomes and unique proteomes were used to study the proteomic cohesiveness of several bacterial species, revealing that some bacterial species had little cohesiveness in their protein content, with some having fewer proteins unique to that species than randomly-chosen sets of isolates from the same genus.

**Conclusions:**

The results described in this study aid our understanding of protein content relationships in different bacterial groups, allowing us to make further inferences regarding genome-environment relationships, genome evolution, and the soundness of existing taxonomic classifications.

## Background

Historically, taxonomic analyses have been performed using a diverse and often arbitrary selection of morphological and phenotypic characteristics. Today, these characteristics are generally considered unsuitable for generating reliable and consistent taxonomies for prokaryotes, as there is no rational basis for choosing which morphological or phenotypic properties should be examined. Moreover, it is doubtful that individual phenotypes or small collections of phenotypes can consistently and correctly represent evolutionary relationships [1]. The unsuitability of phenotypic traits, along with the advent of DNA sequencing, has led to 16S rRNA gene sequence comparisons becoming the standard technique for taxonomic analyses [1], although it has been argued that the *cpn60 *gene allows for greater evolutionary discrimination [2]. Over time, the trend has moved toward using a greater number of genes to infer phylogenetic relationships--in part due to the increasing ease and reduced cost associated with DNA sequencing, but also due to doubts about the accuracy of evolutionary relationships inferred from a single gene. Phylogeny can be inferred from a number of universally conserved housekeeping genes using multi-locus sequence analysis (MLSA) [3,4].

While 16S rRNA gene sequence analysis and MLSA have proven to be effective tools for phylogenetics, a major deficiency inherent in these techniques is that only a small amount of information is used to represent an entire organism. This practice has largely been accepted due to the time and cost of genome sequencing. However, recent improvements in sequencing technology have substantially reduced the resources necessary to sequence a genome, and there are now numerous genome sequences available in publicly accessible databases. The accelerating pace of genome sequencing provides the opportunity to explore the use of entire genomes in analyzing evolutionary relationships.

Numerous approaches to determining relatedness via whole genomes have been devised (reviewed in [5]), with examples being dinucleotide frequencies [6], G + C content [7], codon usage [8,9], gene order [10], and oligopeptide composition [11,12]. Yet another approach to whole-genome phylogenetics is the comparison of gene content. This technique works by predicting orthologues in pairs of organisms and then assigning a "distance" between each pair based on the putative number of shared genes. This technique was originally proposed by Snel et al. [13] and was subsequently revisited with larger groups of organisms [14,15]. However, horizontal gene transfer is a major complicating factor in using these methods to infer evolutionary relationships in prokaryotes [16].

Recently, a new subfield called pan-genomics has become established as a framework for exploring the genomic relatedness of bacterial groups. Unlike the studies cited in the previous paragraph, pan-genomics does not involve inferring phylogeny from genome content; rather, it encompasses broad-based characterizations of gene- or protein-content relationships in a given group of organisms. Pan-genomics was introduced by Tettelin et al. [17], who sequenced several strains of the bacterium *Streptococcus agalactiae *and then analyzed the genomic diversity of those isolates in terms of a "core genome" (genes present in all isolates) and a "dispensable genome" (genes not present in all isolates). Two more examples of pan-genomic analyses are those done for *Vibrio *[18] and for *Escherichia coli *[19]. Review articles summarizing concepts and developments in microbial pan-genomics are also available [20,21].

Despite the increasing interest in pan-genomics, we do not know of a study providing a general characterization and comparison of gene/protein content relationships in many different bacterial groups. To fill this gap, this study reports the results of several different analyses that compare the protein content of different bacteria. When beginning this study, we were faced with the choice of comparing either gene content or protein content. Both have been examined in previous work; for example, Tettelin et al. [17] studied both gene sets and predicted protein sets, whereas Rasko et al. [19] used predicted proteins exclusively. For two reasons, we chose to explore protein content rather than gene content. First, since protein content is more directly related to function and physiology than gene content, the use of protein content was more appropriate for relating pan-genomic properties to factors like habitats, environmental niches, and selective pressures. Second, since we perform comparisons across diverse genera, the lower level of variability in protein sequences compared to gene sequences (due to the degeneracy of the genetic code) may provide an advantage when using BLAST to compare the more divergent organisms. The popularity of tools such as tblastx [22,23] also speaks to the desirability of comparing gene sequences via the corresponding proteins. While we expect the use of gene content versus protein content to yield largely similar results, the reader should be aware that there could be some differences.

This paper communicates the results of three major analyses, with the first two involving protein content comparisons at the genus level, and the third involving comparisons at the species level. In the first analysis, we quantify and analyze the number of proteins (i.e. orthologues) found in all members of a given bacterial genus (its "core proteome"), the number of proteins found in one genus, but in none of the other genera used in this study (its "unique proteome"), and the number of proteins found in only a single isolate of a genus ("singlets"). The second analysis examines the relationship between protein content similarity and 16S rRNA gene percent identity in pairs of bacterial isolates from the same genus. Finally, the third analysis examines several bacterial species to determine whether their proteomes are more cohesive than randomly-selected sets of isolates from the same genus. For the third analysis, we use an operational definition of "cohesion". Specifically, we say that a bacterial species is proteomically cohesive if it satisfies two criteria: first, that its core proteome is larger than those of randomly-selected groups of isolates from the same genus; and second, that it contains more proteins unique to all members of that species than there are proteins unique to randomly-selected groups of isolates from the same genus.

## Results and Discussion

### Proteomes used

Sixteen genera met the requirements outlined in the Methods section, comprising a total of 211 isolates from 106 species. Table 1 shows the number of isolates and species used for each genus, while additional file 1 provides more detailed information about each individual isolate (i.e. genus, species, strain/isolate identity, proteome size, and genome size).

**Table 1:** Bacteria used in this study

Genus	***N***_***I***_	***N***_***S***_
*Bacillus*	16	10
*Brucella*	8	5
*Burkholderia*	19	10
*Clostridium*	19	10
*Lactobacillus*	15	12
*Mycobacterium*	14	11
*Neisseria*	6	2
*Pseudomonas*	15	7
*Rhizobium*	4	2
*Rickettsia*	11	9
*Shigella*	7	4
*Staphylococcus*	18	4
*Streptococcus*	31	9
*Vibrio*	8	5
*Xanthomonas*	8	3
*Yersinia*	12	3

For each bacterial genus used in this study, the number of isolates used (*N*_*I*_), as well as the number of species (*N*_*S*_), is indicated.

### Orthologue detection

To detect orthologues, we used a variation on the reciprocal BLAST hits (RBH) method. Specifically, for two proteins to be declared orthologues, they had to be each other's best BLAST hit, and both BLAST hits had to attain E-values less than a defined threshold. The Methods section describes an analytical method for choosing this E-value threshold, as well as an empirical technique for estimating the degree to which the chosen E-value threshold will affect our analyses. In this section, we apply those techniques to choose an appropriate E-value threshold for the comparisons done in this study.

#### Analytical method

In the Methods section, we show that an appropriate E-value threshold can be chosen using the equation $\text{E}=M/(n_{p}^{2}n_{o}^{2})$, where *E *is the E-value threshold, *M *is the desired value for the expected number of spurious matches, *n*_*p *_is the number of proteins in a given organism's proteome, and *n*_*o *_is the number of organisms involved in a comparison. In choosing a threshold for the comparisons used in this study, we noted that the bacterial isolate examined in this paper with the largest genome, *Burkholderia xenovorans *strain LB400, encodes 8951 ≈ 10^4 ^proteins. Thus, a conservative value for *n*_*p *_would be 10^4^. Furthermore, the greatest number of organisms used in a single comparison was *n*_*o *_= 211 (when finding proteins unique to a given genus). Finally, we chose *M *= 1, since the results of a given comparison would be only negligibly affected by a single spurious match. Thus, the chosen E-value threshold was E = 1/((10^4^)^2 ^× 211^2^) ≈ 10^-13^, meaning that two proteins were considered orthologues if the matches between the two proteins (in both directions) had E-values less than 10^-13^, in addition to each being the other's best BLAST hit.

#### Empirical method

**Figure 1:**
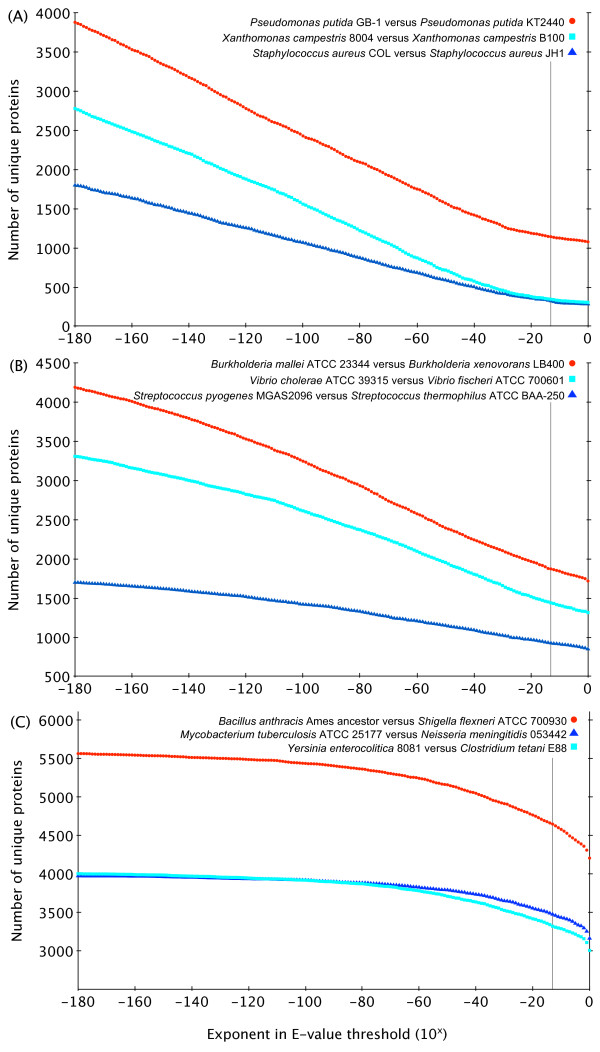
**Relationship between the E-value threshold and numbers of unique proteins in pairs of isolates**. For a given comparison, these graphs denote the number of proteins in the first isolate (e.g. *Pseudomonas putida *GB-1) that are not found in the second isolate (e.g. *Pseudomonas putida *KT2440). The relationship between pairs of isolates is: (A) same species; (B) same genus but different species; and (C) different genera. As an E-value threshold of 10^-13 ^was ultimately chosen for our analyses, a vertical line corresponding to this E-value is indicated on each graph.

To estimate the potential impact of the choice of E-value threshold on our analyses, three pairs of proteomes were arbitrarily selected in each of three categories: isolates from the same species; isolates from different species but the same genus; and isolates from different genera. These three categories were selected as they span the range of relatedness encountered in our analysis. For each pair of proteomes, the orthologue detection procedure described in the Methods section was used to determine the number of proteins in the first proteome, but not in the second proteome, over the range of E-value thresholds 10^0^, 10^-1^,...,10^-180^. Figure 1 shows the number of unique proteins for each comparison for each E-value threshold used.

For all three comparisons in all three categories, the number of unique proteins differed substantially depending on the E-value threshold chosen. For example, the number of proteins found in the proteome of *Pseudomonas putida *strain GB-1 but not in that of *P. putida *strain KT2440 (see Figure 1A) ranged from 3882 when using an E-value threshold of 10^-180 ^to 1075 when using a threshold of 10^0^. The plot for *P. putida *can be divided into two distinct sections. The first section of the plot ranged from an E-value threshold of 10^180 ^to a threshold of approximately 10^-31^, in which there was a nearly perfectly linear decrease in the number of unique proteins as the exponent in the E-value threshold was increased. The second section ranged from E-value thresholds between 10^-30 ^and 10^0^. Like the first section, the number of unique proteins decreased as the E-value threshold was increased, although the slope was much smaller. In other words, compared to the first section, increasing the E-value threshold in this region seemed to result in smaller decreases in the number of unique proteins. This same trend was observed in the other two intra-species comparisons. Owing to the more divergent sequences of their proteins, all three inter-genus comparisons (Figure 1C) showed a distinctly different pattern--a very gradual slope between thresholds of 10^-180 ^and 10^-51^, and then a steeper slope between thresholds of 10^-50 ^and 10^0^. As expected, the trend seen in all three inter-species (but intra-genus) comparisons (Figure 1B) was intermediate between the intra-species and inter-genus comparisons.

Figure 1 shows that, while the number of unique proteins differed substantially over the full range of E-value thresholds tested, the values did not differ by much over the range of E-value thresholds that might reasonably be chosen (say, between 10^-30 ^and 10^-2^). For example, Figure 1A shows that *P. putida *strain GB-1 had 1097 proteins not found in *P. putida *strain KT2440 at an E-value threshold of 10^-3^, versus 1144 at a threshold of 10^-13^. Similarly, Figure 1C shows that *Yersinia enterocolitica *had 3185 proteins not found in *Clostridium tetani *at a threshold of 10^-3^, versus 3322 at a threshold of 10^-13^. As the magnitudes of these differences are small, and because an E-value threshold of 10^-13 ^is justified by the above analytical method, we used this threshold for the rest of our analyses.

### Comparing the protein content of selected genera

#### Identification of core proteomes, unique proteomes, and singlets

**Figure 2:**
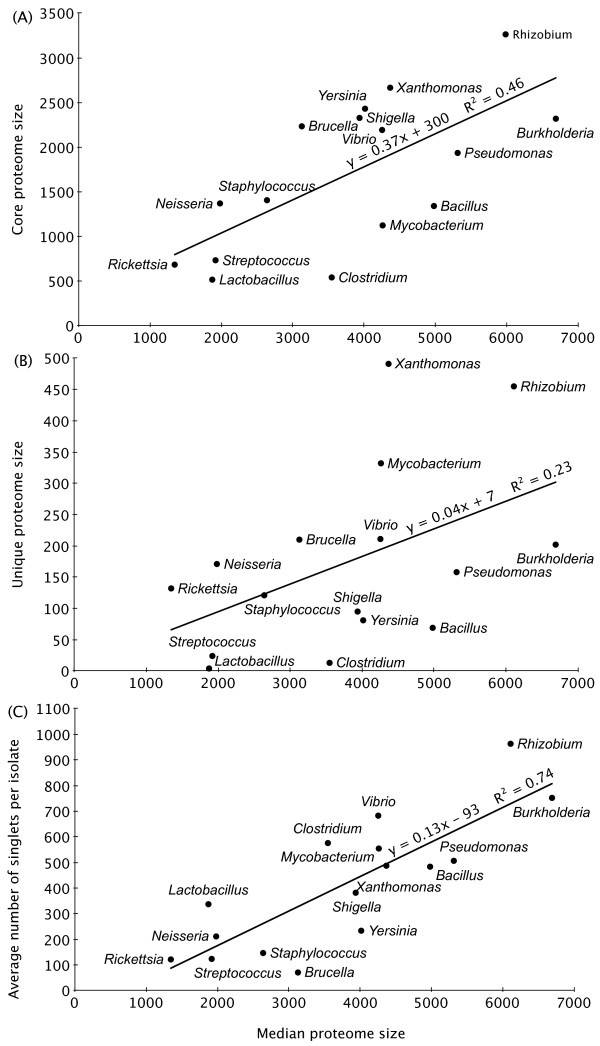
**Comparison of the protein content characteristics of selected genera**. For each of the bacterial genera listed in Table 1, the relationship is given between the median proteome size of a genus and (A) its core proteome size, (B) its unique proteome size, and (C) the average number of singlets per isolate.

To provide a general characterization of pan-genomic relationships in different genera, the orthologue detection procedure described in the Methods section was used to find core proteomes, unique proteomes, and singlets for each of the 16 genera listed in Table 1. If a given orthologous group contained proteins from all isolates of a given genus, it was considered to be part of the core proteome for that genus. If a given orthologous group contained proteins from all isolates of a given genus *and *no proteins from any other isolate in any of the other genera given in Table 1, then it was considered to be part of the unique proteome for that genus. Finally, if a given group contained just a single protein from a single isolate of a given genus, then it was referred to as a singlet. Note that although a singlet protein for a given isolate could not have been found in any other isolates from the same genus (by definition), it may have been found in the proteomes of isolates from other genera. Figure 2 displays the relationship between a genus's median proteome size and its core proteome size (A), its unique proteome size (B), and the average number of singlets per isolate (C). We compared against the median proteome size rather than the mean to eliminate the effect of outliers, since some genera have one or more isolates with far larger or smaller proteomes than most other isolates from the same genus.

Figure 2A shows that the different genera varied significantly in the ratio of their median proteome size to their core proteome size. Genera appearing below the best-fit line had a larger ratio of median proteome size to core proteome size than those appearing above the line. This ratio could be interpreted as showing the relative proteomic similarity of the isolates of each genus. For example, if genus *A *has a very low ratio, then many proteins found in a given isolate of genus *A *are actually found in all genus *A *isolates, whereas if genus *B *has a very high ratio, then many proteins found in a given isolate of genus *B *are not found in all genus *B *isolates. To use the language of Tettelin et al. [17], genera with a high ratio contain isolates that generally have large dispensable genomes, and vice versa.

The fact that genera such as *Lactobacillus *and *Clostridium *had a large ratio is consistent with reports that characterize the taxonomic classifications of these genera as overly broad. For instance, Ljungh and Wadstrom [24] argued that *Lactobacillus *should be split up into a number of separate genera, and Collins et al. [25] made a similar argument for *Clostridium*. On the other side of the spectrum, *Brucella *and *Xanthomonas*, among others, had low median proteome size to core proteome size ratios. This is consistent with the fact that all pairs of isolates in each of these two genera had 16S rRNA genes that were more than 99.5% identical to each other (see also the next section, which provides a comparison of proteomic similarity with 16S rRNA gene similarity).

The best-fit line in Figure 2A had an *R*^2 ^value of 0.46, showing that the median proteome size of a given genus explained less than half of the variation in core proteome size. Another factor that could explain differences in core proteome sizes is simply the number of isolates used, since the core proteome size of a given genus can only decrease (or remain the same) as more isolates are added to the analysis. In their report on the pan-genomics of *Streptococcus agalactiae *[17], for example, Tettelin and co-authors showed that, as additional isolates were added, the core genome of this species decreased in a fashion consistent with a decaying exponential function, eventually approaching some asymptotic value. Other factors that could explain differences in core proteome sizes include the quality of a genus's taxonomic classification, the frequency of horizontal gene transfer, the number of mobile genetic elements (e.g. plasmids), and the nature and variety of environments that the isolates inhabit.

The proteins comprising the core proteome of a given genus could be considered the fundamental units of information required for the existence of isolates of that genus as they currently exist in their environments, and include both housekeeping proteins and proteins required for environment-specific functions. The latter category of proteins would be the most informative in terms of characterizing the commonalities of a given group of bacteria. For instance, the protein encoded by the *acpM *gene, which is involved in mycolic acid synthesis [26], comprises part of the core proteome of the *Mycobacterium *genus, and thus is part of the unique lipid metabolism that characterizes mycobacteria. As a greater number of core proteomes are revealed through additional genome sequencing, core proteomes may be capable of revealing the fundamental requirements for life in relation to basal function or to specific niches, habitats, and diseases. 

Whereas the core proteome is the set of proteins that a particular group of bacteria have in common, the unique proteome is what makes a group different from other groups (i.e. would not include conserved housekeeping proteins). The relationship between median proteome size and unique proteome size for the genera used in this study is given in Figure 2B. The trend was somewhat similar to that shown in Figure 2A, with both *Lactobacillus *and *Clostridium *having very few unique proteins and *Xanthomonas *having many unique proteins. However, there were some interesting differences. For instance, *Mycobacterium *had a fairly small core proteome, but had a larger unique proteome than all genera except *Xanthomonas *and *Rhizobium*. We hypothesized that this may be a reflection of the diverse lipid metabolism of mycobacteria, which among other things provides these organisms with their unique cell wall structure [27]. *Mycobacterium tuberculosis *strain H37Rv, for instance, contains around 250 enzymes for fatty acid biosynthesis alone, compared to a fifth of that for *E. coli *[28]. To tentatively examine this hypothesis, we analyzed the annotations of the 332 proteins unique to the mycobacteria. We report data here for a representative isolate, *Mycobacterium ulcerans *strain Agy99. Many of the 332 proteins were associated, in this isolate, with the structure or synthesis of the cell membrane, with 83 membrane proteins, 12 transferases, and 17 lipoproteins. In addition, 65 of the proteins were uncharacterized, and it is plausible that many of these uncharacterized proteins may also be associated with the mycobacterial cell wall, since our knowledge of its biology is still far from complete [29,30].

The *R*^2 ^value of 0.23 for the best-fit line indicates that median proteome size explains little of the variation in unique proteome size. It is likely that much of this variation could be explained by some of the same factors mentioned for core proteome size, in particular the environments inhabited by a particular genus and the amount of specialization required to adapt to those environments.

The unique proteome of a given group of bacteria (not necessarily a genus) can be regarded as the protein complement that makes it distinct from other taxonomic groups. The DNA sequences of the open reading frames corresponding to the unique proteome would therefore be good candidates for group-specific identification methods, such as group-specific PCR. Given that PCR-based identification methods require conserved regions in the DNA sequences, the unique proteome would provide a broad range of possible targets. Conserved regions of DNA have been used for group-specific identification before; for instance, three of us performed phylum-specific PCR using conserved regions in the 16S rRNA gene as targets [31,32]. As another example, O'Sullivan et al. [33] determined orthologous relationships among the genes in several lactic acid bacteria in order to identify niche-specific (specifically, gut-specific and dairy-specific) genes.

Another interesting application of unique proteomes could be to strengthen the argument for the taxonomic reclassification of certain genera. For example, the *Lactobacillus *genus had a very small unique proteome compared to other genera. While this fact alone would not be enough to show that the taxonomy of *Lactobacillus *should be re-examined, it does help support this contention in combination with other data (e.g. [24]). If care is used in the selection of groups, unique proteomes could also provide insight on factors or evolutionary trends leading to virulence, adaptation to specific environmental niches, or currently-unknown metabolic functions.

In contrast to the core and unique proteomes, the average number of singlets per isolate in a given genus (Figure 2C) exhibited a fairly strong relationship with the median proteome size (*R*^2 ^= 0.74). This was not surprising, since one would expect the number of singlets to increase with proteome size. Nonetheless, it is still rather striking that most isolates have hundreds of proteins not found in any other isolate from the same genus, reflecting the sheer amount of diversity in the protein content of even very closely related organisms. This is consistent with previous observations that new genes continue to be added to a given bacterial species with each new genome sequenced, and thus that it may be impossible to ever fully describe a given species in terms of its collective genome content [21].

Whereas unique proteins may be useful for developing genus-specific (or, more generally, group-specific) identification techniques, singlets would be similarly useful for facilitating strain-specific identification. Additionally, whereas the core proteome represents the protein complement necessary for life among all the niches and habitats occupied by the different strains of a given group, singlets could be linked to more specific lifestyle requirements of a single strain.

#### Comparison of proteomic similarity with 16S rRNA gene similarity

Phylogenetic studies currently use 16S rRNA gene sequence comparisons as the standard method for the taxonomic classification of prokaryotes. Two isolates are typically described as being of the same species if their 16S rRNA genes are more than 97% identical, and of the same genus if their 16S rRNA genes are more than 95% identical [34], although our data (see Table 2) suggest that the lower limit for a genus is closer to 90% (and *Clostridium *and *Lactobacillus *represent exceptions even to this boundary, as some pairs of isolates in these genera have identities well below 90%). However, analogous thresholds for proteomic similarity--if they exist--are currently unknown. Additionally, while other studies have reported a relationship between genomic similarity and identity of the 16S rRNA gene, no statistical correlation has been reported (a substantial review of this topic is given by Rosello-Mora and Amann [35]). We therefore sought to investigate the relationship between protein content similarity and 16S rRNA gene similarity in pairs of isolates from the same genus. In doing so, we used two different measures of proteomic similarity: "shared proteins" (the number of proteins found in the proteomes of both isolates--in other words, the number of orthologues), and "average unique proteins" (the average of the number of proteins found in isolate A but not isolate B, and the number of proteins found in isolate B but not isolate A). For a given genus, both of these proteomic similarity measures were plotted against the 16S rRNA gene percent identity for all pairs of isolates, and linear regression was used to describe the nature of the relationship (slope and *R*^2 ^value) between these variables. As described in the Methods section, only pairs of isolates whose 16S rRNA genes were less than 99.5% identical were included in this analysis. As a result, no slope and *R*^2 ^values could be determined for *Brucella *and *Xanthomonas*, as no pairs of isolates within these genera had 16S rRNA gene percent identities less than this cutoff. Table 2 contains the results of these analyses.

**Table 2:** Results of comparison between protein content similarity and 16S rRNA gene percent identity

Genus	16S range	Shared proteins			Average unique proteins		
		
		Range	Slope	***R***^**2**^	Range	Slope	***R***^**2**^
*Bacillus*	90.4-100%	1741-5204	231	0.83*	248-3000	-176	0.69*
*Brucella*	99.9-100%	2495-3060	ND^a^	ND	154-454	ND^a^	ND
*Burkholderia*	93.8-100%	2861-6337	192	0.26*	337-4554	-394	0.67*
*Clostridium*	80.3-100%	917-3333	38	0.47*	141-2987	-60	0.36*
*Lactobacillus*	85.8-100%	720-2348	42	0.49*	235-1595	-46	0.19*
*Mycobacterium*	91.3-100%	1258-4327	99	0.13*	87-2994	-151	0.47*
*Neisseria*	98.4-100%	1470-1794	-263	0.19	206-753	305	0.03
*Pseudomonas*	93.1-100%	2368-5339	68	0.06*	383-2847	-129	0.37*
*Rhizobium*	98.9-99.9%	3482-4690	178	0.03	1296-2095	12	0.00
*Rickettsia*	97.2-100%	743-1275	92	0.49*	48-556	51	0.07
*Shigella*	97.4-99.7%	2781-3481	122	0.13	463-1185	-113	0.11
*Staphylococcus*	97.4-100%	1674-2653	72	0.41*	49-923	-18	0.02
*Streptococcus*	92.6-100%	929-1954	46	0.28*	84-1028	-35	0.15*
*Vibrio*	90.9-99.8%	2345-3879	142	0.81*	396-2167	-21	0.03
*Xanthomonas*	99.8-100%	2802-3982	ND	ND	201-1653	ND	ND
*Yersinia*	97.2-100%	2675-3825	347	0.94*	216-1319	-27	0.94*

For each genus, the range of 16S rRNA gene percent identities for all pairs of isolates from that genus is listed. Under the "shared proteins" heading, "range" indicates the range of shared proteins in pairs of isolates from that genus. The "slope" column indicates the slope of the regression line when the number of shared proteins in each pair of isolates is plotted against their 16S rRNA gene percent identities. The "*R*^2^" column contains the square of the standard correlation coefficient between these two variables, and indicates the strength of their relationship. The data under the "average unique proteins" heading are analogous to those under the "shared proteins" heading. Isolates sharing ≥ 99.5% identity of the 16S rRNA gene were not used in the calculation of slope or *R*^2^. Values marked with "ND" were not determined; despite having different species names, all isolates with sequenced genomes within these genera shared ≥ 99.5% identity of the 16S rRNA gene. An asterisk (*) beside an *R*^2 ^value indicates that it is statistically significant with P-value < 0.05.

In contrast to 16S rRNA gene percent identity, Table 2 shows that there is no specific range of proteomic diversity for a genus. In other words, although a reasonably consistent cutoff has traditionally been used for bounding the 16S rRNA gene identity of isolates from the same genus, there does not seem to be a corresponding lower limit for shared proteins or upper limit for average unique proteins.

Table 2 indicates that most genera exhibited a direct relationship between shared proteins and 16S rRNA gene percent identity, and an inverse relationship between average unique proteins and 16S rRNA gene percent identity. This was expected given that larger numbers for the shared proteins measure indicate greater similarity, whereas larger numbers for the average unique proteins measure indicate greater dissimilarity. Interestingly, however, *Neisseria *exhibited the opposite trend; also anomalous were *Rickettsia *and *Rhizobium*, which had positive slopes for both proteomic similarity metrics. Surprisingly, the relationship between 16S rRNA gene similarity and protein content similarity was fairly weak for most genera. Specifically, only four of the 14 genera exhibited a strong (*R*^2 ^> 0.5) relationship between 16S rRNA gene identity and either of the proteomic similarity measures. Two of these genera (*Bacillus *and *Yersinia*) showed a strong relationship between 16S rRNA gene identity and both proteomic similarity measures, whereas *Vibrio *exhibited a strong correlation only for the shared proteins measure and *Burkholderia *had a strong correlation only for the average unique proteins measure.

Perhaps most interestingly, the *R*^2 ^values for the shared proteins measure and the average unique proteins measure were sometimes quite different even for the same genus. This could be attributed to the fact that the number of shared proteins in two isolates is a measure of gene conservation, whereas the average number of unique proteins in two isolates is a measure of gene gain or loss. For example, the *R*^2 ^value for *Vibrio *when using the shared proteins measure was 0.81, compared to just 0.03 when using the average unique proteins measure. This could indicate that a subset of genes were highly conserved over time while a large amount of gene loss/acquisition occurred, which ultimately enabled *Vibrio *isolates to inhabit the various niches in which they are currently found.

**Figure 3:**
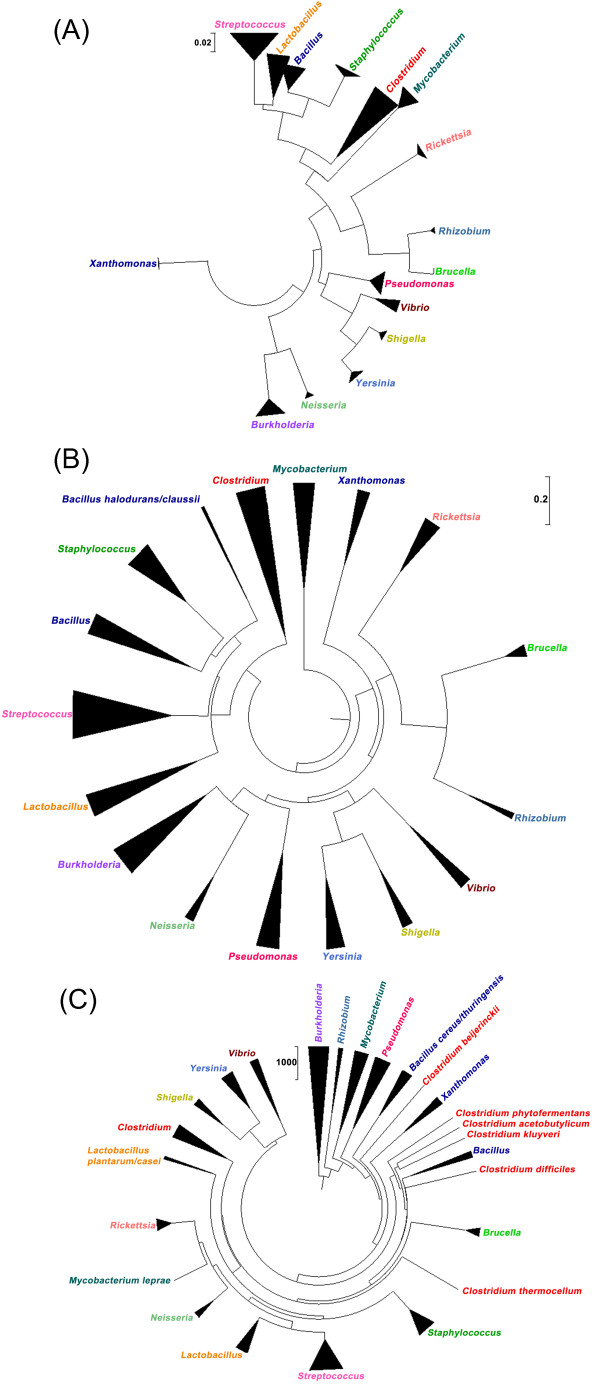
**Phylogenetic relationships among the organisms used in this study**. Three phylogenetic trees were constructed, each of which used a different distance metric. Panel (A) depicts the tree constructed using the 16S rRNA gene similarity between two isolates, while panels (B) and (C) depict trees based on shared proteins and average unique proteins, respectively. Due to space constraints, collapsed trees are shown; the full trees are available as additional files 2, 3, and 4. The length of the base of each triangle represents the number of species within the genus, while the height indicates the amount of intra-genus divergence.

As described in the Methods section, we also created three phylogenetic trees, with the first based on 16S rRNA gene similarity, the second based on the number of shared proteins between two isolates, and the third based on the average unique proteins between two isolates. Collapsed versions of these trees are given in Figures 3A, 3B, and 3C, respectively, while trees showing all individual isolates are available as additional files 2, 3 and 4.

There are several notable observations that can be made through comparisons of these three phylogenetic trees. For the most part, the trees were similar; for example, the intra-genus diversity was large for *Lactobacillus *and *Clostridium *in all three phylogenetic trees (demonstrated by the height of each triangle). However, the methods based on protein content did sometimes give results different from those given by the method based on 16S rRNA gene similarity, which is typically used for nomenclature. Notably, the *Bacillus *genus was divided in both protein content-based trees, but not in the tree based on the 16S rRNA gene. Additionally, there were marked differences between the shared protein method (proposed by Snel et al. [13]) and the average unique proteins method (introduced in this paper). The shared proteins method resulted in a taxonomy fairly similar to that found when using the 16S rRNA gene, suggesting that their respective rates of evolution are similar. Conversely, the average unique proteins method gave a somewhat different view of taxonomy. For example, the genus *Clostridium *has been described as extremely heterogeneous [25], and this is reflected in the divergence of some species of this genus from the rest of the clostridia in the average unique proteins tree. As another example, the species *Lactobacillus casei *and *Lactobacillus plantarum *both have much larger proteomes than other lactobacilli, which is likely the cause of their divergence from the rest of their genus.

It is a widely held assumption that the 16S rRNA gene is one of the few genes that can be regarded as an approximate molecular clock, and that other genes--and the genome as a whole--can have a very different rate of evolution compared to the 16S rRNA gene, due to various selective pressures and horizontal gene transfer [1]. Table 2 represents a quantitative approach to examining the relationship between the evolutionary relatedness of different organisms (as measured by the similarity of their 16S rRNA genes) and their degree of genomic similarity (as measured by shared proteins or average unique proteins). It seems reasonable to hypothesize that a stronger relationship between 16S rRNA gene similarity and proteomic similarity for a given genus would imply a lower selective pressure on the organisms' genomes, and vice versa. This difference in selective pressure may in turn reflect the fact that different genera live in different environments, or that the organisms belonging to a given genus may inhabit a greater variety of environments than the organisms belonging to a second genus. As evolutionary pressures experienced by organisms differ based on their environmental niche and life cycle, we expect to see different patterns of association between 16S rRNA gene identity and proteomic content emerge as a greater number of genome sequences become available.

### Comparing the protein content of selected species

#### Evaluating taxonomic classifications by determining how well species are clustered based on protein content

In this section, we provide a novel perspective on the soundness of the taxonomic classifications of different species. Broadly speaking, the classification of a set of organisms into a single species could be described as "good" if two criteria are met: the organisms are very similar to each other, and they are distinct from other organisms of the same genus. This section reports the results of examining these two criteria from the perspective of protein content; specifically, the isolates of a given species are considered to be similar to each other if they have a larger core proteome than randomly-selected sets of isolates of the same genus, and are considered to be distinct from other organisms of the same genus if they have a larger unique proteome than randomly-selected sets of isolates of the same genus.

For each species from the genera listed in Table 1 that had two or more isolates sequenced, we compared the core proteome size and the unique proteome size of that species to those of randomly-generated sets of isolates from the same genus. The results of this analysis are given in Tables 3 and 4. Also, additional file 5 contains the organisms comprising each random group, as well as the core proteome size and unique proteome size of each.

**Table 3:** Results of protein content cohesiveness experiments

		Core proteomes				Unique proteomes			
		
*S*	***N***_***I***_	$N_{C}^{A}$	$N_{C}^{R}$	***P***_***C***_	$N_{C}^{>}$	$N_{U}^{A}$	$N_{U}^{R}$	***P***_***U***_	$N_{U}^{>}$
*Bacillus anthracis*	3	4941	2123	**	0/25	168	1	**	0/25
*Bacillus cereus*	4	2881	1840	**	0/25	2	0	-	0/25
*Bacillus thuringiensis*	2	4255	2864	**	5/25	4	7	n.s.	7/25
*Brucella abortus*	3	2699	2603	**	6/25	2	1	*	4/25
*Brucella suis*	2	3025	2760	**	2/24	5	4	n.s.	5/24
*Burkholderia ambifaria*	2	5609	3798	**	1/25	198	17	**	0/25
*Burkholderia cenocepacia*	3	5908	3352	**	0/25	168	0	**	0/25
*Burkholderia mallei*	4	3623	3086	**	1/25	18	0	-	0/25
*Burkholderia pseudomallei*	4	4972	3086	**	0/25	45	0	-	0/25
*Clostridium botulinum*	8	1514	763	**	0/25	10	0	-	0/25
*Clostridium perfringens*	3	2110	1085	**	0/25	298	0	**	0/25
*Lactobacillus casei*	2	2355	959	**	0/25	593	5	**	0/25
*Lactobacillus delbrueckii*	2	1372	959	**	0/25	222	5	**	0/25
*Lactobacillus reuteri*	2	1402	959	**	0/25	120	5	**	0/25
*Mycobacterium bovis*	2	3822	2577	**	1/25	36	38	n.s.	3/25
*Mycobacterium tuberculosis*	3	3724	2118	**	0/25	26	17	n.s.	3/25
*Neisseria gonorrhoeae*	2	1795	1560	**	0/8	229	3	**	0/8
*Neisseria meningitidis*	4	1547	1426	**	0/14	75	4	**	0/14

Column headings are: *S*, species; *N*_*I *_, number of sequenced isolates of species *S*; $N_{C}^{A}$, core proteome size of the sequenced isolates of *S*; $N_{C}^{R}$, average core proteome size of the randomly-generated sets; *P*_*C*_, probability that the average core proteome size of the randomly-generated sets is different than the core proteome size of the sequenced isolates of *S*; $N_{C}^{>}$, fraction of random sets having a core proteome larger than *S*. $N_{U}^{A}$, $N_{U}^{R}$, *P*_*U *_and $N_{U}^{>}$ are analogous to $N_{C}^{A}$, $N_{C}^{R}$, *P*_*C*_, and $N_{C}^{>}$, respectively, and refer to the comparisons involving the number of proteins found in all sequenced isolates of *S*, but no other isolates from the same genus ("unique proteomes"). In some cases, all of the random sets corresponding to a particular species had zero unique proteins. No P-value could be computed for these because the standard deviation of these values was zero. In these situations, the *P*_*U *_column contains a dash character (-). The averages in both column $N_{C}^{R}$ and column $N_{U}^{R}$ are rounded to the nearest whole number. For certain rows, column $N_{U}^{R}$ shows a value of 0; in some cases, this value is exact, while in other situations, it is due to rounding. If due to rounding, then the standard deviation of the random sets is non-zero, and column *P*_*U *_contains a P-value. For columns *P*_*C *_and *P*_*U *_, "n.s." means "not significant", a single asterisk indicates a P-value of less than 0.05, and a double asterisk indicates a P-value of less than 0.001. See Table 4 for the continuation of this table.

**Table 4:** Results of protein content cohesiveness experiments (continued)

		Core proteomes				Unique proteomes			
		
Species	***N***_***I***_	$N_{C}^{A}$	$N_{C}^{R}$	*P*_*C*_	$N_{C}^{>}$	$N_{U}^{A}$	$N_{U}^{R}$	*P*_*U*_	$N_{U}^{>}$
*Pseudomonas aeruginosa*	3	4959	2877	**	0/25	571	1	**	0/25
*Pseudomonas fluorescens*	2	4206	3199	**	0/25	142	6	**	0/25
*Pseudomonas putida*	4	3799	2592	**	0/25	69	0	**	0/25
*Pseudomonas syringae*	3	3894	2877	**	0/25	290	1	**	0/25
*Rhizobium etli*	2	4700	4063	n.s.	0/4	431	176	n.s.	0/4
*Rhizobium leguminosarum*	2	3678	4063	n.s.	2/4	148	176	n.s.	2/4
*Rickettsia bellii*	2	1277	850	**	0/25	219	1	**	0/25
*Rickettsia rickettsii*	2	1221	850	**	0/25	93	1	**	0/25
*Shigella boydii*	2	3170	2989	**	1/17	95	12	**	0/17
*Shigella flexneri*	3	3255	2770	**	0/25	130	6	**	0/25
*Staphylococcus aureus*	14	1917	1486	**	0/25	157	0	**	0/25
*Staphylococcus epidermidis*	2	2080	1798	**	0/25	131	0	**	0/25
*Streptococcus agalactiae*	3	1688	1019	**	0/25	156	0	-	0/25
*Streptococcus pneumoniae*	6	1543	922	**	0/25	150	0	-	0/25
*Streptococcus pyogenes*	13	1348	811	**	0/25	49	0	-	0/25
*Streptococcus suis*	2	1971	1087	**	0/25	336	0	**	0/25
*Streptococcus thermophilus*	3	1359	1019	**	0/25	145	0	-	0/25
*Vibrio cholerae*	2	3384	2764	**	1/25	425	20	**	0/25
*Vibrio fischeri*	2	3380	2764	**	1/25	447	20	**	0/25
*Vibrio vulnificus*	2	3882	2764	**	0/25	321	20	**	0/25
*Xanthomonas campestris*	4	3376	2818	**	0/25	49	4	**	0/25
*Xanthomonas oryzae*	3	3276	2915	**	5/25	299	0	**	0/25
*Yersinia pestis*	7	2986	2717	**	4/25	21	0	**	0/25
*Yersinia pseudotuberculosis*	4	3424	3003	**	0/25	21	0	**	0/25

For the meanings of each column, see Table 3.

The primary purpose of this section was to investigate the utility of this cohesiveness analysis for identifying bacterial species that might be misclassified. A cursory reading of Tables 3 and 4 revealed that, while most species satisfied both of the above criteria, some species either had core or unique proteomes that were not significantly larger than the average of the random groups, or had several corresponding random groups that had larger core or unique proteomes than the species itself. A lack of cohesiveness in the proteomes of a given species indicates that its taxonomic classification may need revisiting. However, these results must be interpreted with caution. A closer look at these species revealed that the classification of some really did appear to warrant re-examination, whereas the apparent lack of cohesiveness of others had alternative explanations. In the following paragraphs, we discuss several examples. First, we describe the cohesiveness results for *Bacillus anthracis*, which is indeed proteomically cohesive based on Tables 3 and 4. Next, we discuss *Rhizobium leguminosarum *and *Yersinia pestis*, both of which look uncohesive based on these tables but whose lack of cohesiveness can readily be explained. Finally, we look at two species that probably do warrant reclassification, *Bacillus cereus *and *Bacillus thuringiensis*.

As an example of reading Tables 3 and 4, consider the first row of Table 3, which contains *B. anthracis*. The core proteome of the three sequenced *B. anthracis *isolates contained 4941 proteins. When sets of three *Bacillus *isolates were randomly chosen as described in the Methods section, however, the average core proteome size was just 2123. According to a two-tailed t-test, the P-value for this comparison was less than 0.001, indicating that the difference in core proteome size between the three *B. anthracis *isolates, and randomly chosen sets of three *Bacillus *isolates, was statistically significant. In fact, none of the 25 randomly-generated sets contained a larger core proteome than the set of *B. anthracis *isolates. *B. anthracis *therefore satisfied our first criterion, since the three *B. anthracis *isolates had more similar protein content than randomly-chosen sets of three *Bacillus *isolates. *B. anthracis *also satisfied the second criterion, which stated that species should be distinct from other isolates of the same genus. Table 3 shows that the *B. anthracis *isolates contained 168 proteins not found in any other *Bacillus *isolate, compared to an average of just one unique protein for the 25 randomly-generated sets (P-value < 0.001). None of the 25 randomly-generated sets contained more unique proteins than the three *B. anthracis *isolates. Overall, the fact that *B. anthracis *satisfied both criteria supports its current taxonomic classification.

As another example, consider *R. leguminosarum*. There were 3678 proteins in its core proteome, compared to an average of 4063 for randomly selected sets of two *Rhizobium *isolates. This difference was not statistically significant due to the fact that only four corresponding random groups could be created. Two of the four random groups--the first containing *Rhizobium etli *strain ATCC 51251 and *R. leguminosarum *strain 3841, and the second containing *R. etli *strain CIAT 652 and *R. leguminosarum *strain 3841--had larger core proteome sizes than the two *R. leguminosarum *isolates. The results for unique proteomes were similar, with the same two random groups having a larger unique proteome size than the two *R. leguminosarum *isolates. However, this apparent lack of cohesiveness can be attributed to differences in the proteome sizes of the individual isolates: the proteome of *R. leguminosarum *strain WSM2304 contains just 4320 proteins, compared to 5921 for the next-smallest *Rhizobium *isolate. As such, it might be expected that two *Rhizobium *isolates having proteomes much larger than that of *R. leguminosarum *strain WSM2304 would also have a larger core and/or unique proteome.

The apparent lack of cohesiveness of *Y. pestis *can also be readily explained, although the reason is different than that for *R. leguminosarum*. There were four random groups of seven isolates each, all of which contained a mixture of *Y. pestis *and *Yersinia pseudotuberculosis *isolates, that had larger core proteomes than the seven *Y. pestis *isolates. All of the isolates of both *Y. pestis *and *Y. pseudotuberculosis *had proteome sizes that fall within a fairly narrow range (about 3900-4300 proteins), so the larger core proteomes of these random groups cannot be attributed to large differences in proteome sizes. Rather, these results make sense given that *Y. pestis *and *Y. pseudotuberculosis *are very closely related, with *Y. pestis *having recently diverged from *Y. pseudotuberculosis*. However, it is known that *Y. pestis *has acquired additional factors that enable it to cause a very different and severe disease than that caused by *Y. pseudotuberculosis *[36].

Finally, the lack of cohesiveness of some species' proteomes does indeed suggest the need for taxonomic reclassification. For example, *B. cereus *had a much larger core proteome than the randomly generated sets, but had just two unique proteins. While two unique proteins was more than the average for the randomly-generated sets (none of which had any unique proteins), it was much less than the number of unique proteins possessed by other species having four (or more) sequenced isolates. Similarly, *B. thuringiensis *had a larger core proteome than the corresponding random sets, but actually had a smaller unique proteome than the average of the random sets. In addition, the *B. thuringiensis *isolates had fewer unique proteins than seven of the 25 corresponding random sets. Unlike *R. leguminosarum *and *Y. pestis*, we could not identify any reason for the lack of cohesiveness of *B. cereus *and *B. thuringiensis*, other than a possible misclassification. Given that there are many different ways in which the taxonomic classification of a given species can be evaluated, the reclassification of these species could not be justified using only one kind of analysis. However, data like those given in this section could be combined with other kinds of data in order to make a stronger argument. For instance, some of the *B. cereus *and *B. thuringiensis *isolates used in this study in fact have 99-100% 16S rRNA identity with isolates of the opposite species, and a lower percent identity (less than 99%) with isolates of the species to which they are currently assigned. Combined with the very small unique proteomes of *B. cereus *and *B. thuringiensis*, this suggests that there may be isolates named as *thuringiensis *that should really be named as *cereus*, and vice versa. As it can be difficult or uncertain to resolve speciation using only the 16S rRNA gene, using the core/unique proteome analyses introduced here may well assist in the proper naming of isolates that are difficult to speciate.

## Conclusions

In this paper, we examined pan-genomic relationships and their applications in several groups of bacteria. It was found that different bacterial genera vary widely in core proteome size, unique proteome size, and the number of singlets that their isolates contain, and that these variables are explained only partly by differences in proteome size. We also found that the relationship between protein content similarity and the percent identity of the 16S rRNA gene varied substantially in different genera, with a fairly strong association in a few genera and little or no association in most other genera. Finally, we found that most bacterial species were fairly cohesive in their protein content, but that the protein content of some species (such as *B. thuringiensis*) was no more cohesive than that of randomly selected sets of isolates from the same genus, indicating that the current taxonomy of those species may need to be revisited. The differing pan-genomic properties of the various genera reported in this paper reflect the fact that different groups of bacteria have diverse evolutionary pressures and unequal rates of genomic evolution, and provide a starting point for a general, genome-based understanding of such differences in a broad range of bacteria.

We also note that the analyses described in this paper could be applied to any groups of interest, whether or not the bacteria included in each group have a common taxonomic classification. The commonalities in each group could instead be related to phenotype; for example, ability to live in a particular environment, physiological properties, metabolic capabilities, or even disease pathogenesis. As such, the methods described in this paper have broad applicability and should be useful for further pan-genomic comparisons in the future.

There are a number of opportunities to build upon the work performed in this study. For instance, it would be interesting to further characterize proteins that are found in only a single isolate of a given genus (singlets). Our research revealed that the isolates of most genera contain, on average, hundreds of singlets. This phenomenon could be further described by answering questions like: how much variation is there in the number of singlets in isolates of the same genus? Do isolates inhabiting certain environments possess more singlets than other isolates? Do singlets tend to be biased toward any particular functional category of protein? Another avenue for future work would be to enhance our study of the relationship between protein content similarity and 16S rRNA gene similarity. Despite the existence of usually-consistent lower bounds for 16S rRNA gene similarity for isolates of the same genus, in this study we were unable to determine corresponding bounds for protein content similarity. However, we considered only absolute measures of protein content (i.e. absolute numbers of shared proteins or average unique proteins), and it would also be worthwhile to devise biologically meaningful bounds using a relative measure that could take into account factors like the proteome sizes of the individual isolates, the number of individual isolates, and so on. Finally, perhaps the most obvious opportunity for future work is simply to repeat the analyses described in this paper when more genome sequences become available. Given the increasing pace of genome sequencing, in the future it should be possible to do a study similar to this one with dozens or even hundreds of genera, rather than just 16, which will allow us to gain a far richer understanding of the pan-genomic relationships among bacteria.

## Methods

### Proteomes used

A given bacterial genus was used in this study if it met two requirements: first, two or more species of the genus had sequenced genomes; second, at least two of those species had at least two isolates with sequenced genomes. The latter requirement was used so that intra-species comparisons could be conducted. All bacterial proteomes were downloaded on November 28th, 2008 from Integr8 [37](http://www.ebi.ac.uk/integr8 ).

### Orthologue detection

Many techniques have been proposed for identifying orthologous proteins. These include COGs [38-41], Ortholuge [42], OrthologID [43], RIO [44], Orthostrapper [45], and INPARANOID [46,47]. Our analyses involving orthologue detection could theoretically have made use of any of these methods. Unfortunately, it would be difficult to justify choosing one tool over any of the others, and comparing all of the tools with respect to our analyses would have been complicated by the fact that each tool uses different techniques and parameters. As such, in this paper we used a slight variation on the commonly-used RBH method for orthologue detection. With standard RBH, two proteins *P*_1 _and *P*_2 _(from organisms *O*_1 _and *O*_2_, respectively) are considered to be orthologues if and only if: (a) *P*_2 _is the best BLAST [22,23] hit (i.e. having the smallest E-value) when *P*_1 _is used as the query sequence and the proteins in *O*_2 _are used as the database, and (b) *P*_1 _is the best hit when *P*_2 _is used as the query sequence and the proteins in *O*_1 _are used as the database. In our analyses, we imposed an additional criterion: the E-values reported for both comparisons must each be less than some threshold. RBH was chosen because it is a common, well-understood method that is often used as the basis for more complex or specialized approaches to orthologue detection; in addition, the aforementioned variation on RBH requires only a single, though important, parameter--the E-value threshold.

For a given set of organisms, once orthologous relationships between pairs of proteins were determined, a graph was created wherein each vertex represented a protein, and two vertices were connected by an edge if the proteins represented by each were orthologues based on the above RBH-based method. Identification of orthologous groups was then performed by finding the connected components of the graph (i.e. sets of vertices for which there was a path from any vertex to any other vertex) using the Perl module Graph (http://search.cpan.org/dist/Graph/lib/Graph.pod ).

The choice of the aforementioned E-value threshold can affect the results of orthologue detection; as such, it was important to choose this threshold carefully. Below, we describe an analytical method for choosing this threshold, and an empirical method for characterizing the degree to which this threshold would affect our results.

#### Analytical method

In this study, BLAST is used to compare dozens of proteomes, each of which contains thousands of proteins. As such, using a relatively large E-value threshold, such as 0.001, would result in many matches occurring simply by chance. Therefore, we choose a more appropriate threshold using the reasoning shown below.

Suppose that the proteomes of *n*_*o *_organisms are to be compared, and that the number of proteins encoded by the organism with the largest proteome in a given comparison is *n*_*p*_. For each pair of organisms, there will be at most $n_{p}\times n_{p}=n_{p}^{2}$ pairwise comparisons between proteins. The number of pairs of organisms that must be compared (note that comparisons must be performed in both directions) is $n_{o}\times (n_{o}-1)\approx n_{o}^{2}$. Thus, the total number of protein-protein comparisons that must be performed will be bounded above by $n_{p}^{2}n_{o}^{2}$. The expected number of spurious matches *M *will be equal to the number of comparisons performed, multiplied by the probability of a spurious match (*P*) in each comparison. Then

$$M=Pn_{p}^{2}n_{o}^{2}$$

How can a value for *P *be derived? The E-value, simply denoted as *E *in this section, represents for a particular match with raw score *R *the number of matches attaining a score better than or equal to *R *that would occur at random given the size of the database. While *E *does not represent a probability, *P *can be derived from it: since the probability of finding no random matches with a score greater than or equal to *R *is *e*^-*E*^, where *e *is the base of the natural logarithm, the chance of obtaining one or more such matches is *P *= 1 - *e*^-*E *^[48]. Since *P *is nearly equal to *E *when *E *< 0.01, *E *can reasonably be used as a proxy for *P*. As such, the expected number of spurious matches *M *can be written as:

$$M=En_{p}^{2}n_{o}^{2}$$

By rearranging, an equation was obtained that expresses the E-value threshold that should be chosen in terms of *n*_*p*_, *n*_*o*_, and *M*:

$$\text{E}=\frac{M}{n_{p}^{2}n_{o}^{2}}$$

#### Empirical method

To empirically evaluate the impact of the E-value threshold on our orthologue detection procedure, pairs of organisms *A *and *B *were selected, and the number of proteins in the proteome of organism *A *but not in organism *B *(unique proteins) was determined for the E-value thresholds 10^0^, 10^-1^,...,10^-179^, 10^-180^. Scatterplots were then created using these data.

It is reasonable to expect that the relatedness of the organisms involved in a comparison would affect the interaction between the E-value threshold and the number of unique proteins reported. Thus, three different degrees of relatedness were considered--two isolates from the same species; two isolates from the same genus but different species; and two isolates from different genera. These degrees of relatedness were selected as they span the range represented in this report. Three pairs of organisms were arbitrarily selected for each of these three types of comparisons.

### Comparing the protein content of selected genera

#### Identification of core proteomes, unique proteomes, and singlets

To find the core proteome of a particular set of isolates, orthologue detection was performed on the proteins in that set, and connected components of the graph that contained proteins from all isolates in the set were identified. These connected components were then counted to determine the size of the core proteome. It is important to note that the size of the core proteome was defined in terms of the number of orthologous groups, not in terms of the total number of individual proteins (from one specific organism) in those groups. For example, suppose that we were finding the size of the core proteome for a genus with eight isolates, and that there were 500 orthologous groups containing proteins from all eight of those isolates. Further, suppose that each of these groups actually contained ten individual proteins (say, with six isolates having one protein each, and two isolates having two each). Then the size of the core proteome would be reported as 500, not as 500 × 10 = 5000. Unique proteomes were found in a similar manner--by counting the number of connected components that contained proteins from all members of a particular group, but in no members of a second group. Finally, the number of singlets in a particular genus was found by performing orthologue detection on the proteins from that genus (only), and identifying the number of connected components containing just a single protein.

Most comparisons done in this study involved a fairly small number of isolates (and therefore proteins). For example, finding the core proteome of a particular genus involved performing orthologue detection for the isolates of that genus (between 4 and 31 isolates, depending on the genus), each of which had a proteome containing around 1000 to 9000 proteins. However, one type of comparison--finding the proteins unique to each genus--required finding orthologues among all proteins in the proteomes of all isolates used in this study. Due to memory constraints, this could not be done using a single orthologue detection comparison. Instead, comparisons were performed between all possible pairs of genera. For example, in finding the proteins unique to genus *A*, we first determined the list of proteins in all isolates of genus *A*, but no isolates of genus *B*; we then determined the list of proteins found in all isolates of *A*, but no isolates of *C*, and so on. Once all lists had been calculated, the proteins that were present in every list were the proteins unique to genus *A*.

#### Comparison of proteomic similarity with 16S rRNA gene similarity

To determine 16S rRNA gene percent identities, the 16S rRNA gene was obtained from each sequenced genome used in this study and the RDP10 tool [49] was used to align sequences based on known conserved and variable regions according to the rRNA's secondary structure. The percent identity of the 16S rRNA gene between pairs of isolates from the same genus was calculated to the nearest 0.01%. Two methods, both of which used the orthologue detection procedure described above, were used to determine the proteomic similarity between pairs of isolates *A *and *B *(again from the same genus): (a) the number of orthologous groups containing proteins from both isolate *A *and isolate *B *("shared proteins"), and (b) the average of the number of proteins in the proteome of isolate *A *but not isolate *B*, and the number of proteins in the proteome of isolate *B *but not isolate *A *("average unique proteins"). Linear regression using least squares was used to determine the correlation and the equation of the best-fit line between the 16S rRNA gene percent identity and the shared proteins measure, and between the 16S rRNA gene percent identity and the average unique proteins measure.

Preliminary results showed that genera having many very closely related isolates (such as many isolates of the same species) had much higher correlations between 16S rRNA gene percent identity and the two proteomic similarity measures than genera having fewer very closely related isolates. Further analysis revealed that this phenomenon was caused by pairs of these closely related isolates "anchoring" the regression line, leading to an artificially good linear relationship. To avoid this bias, we initially tried excluding pairs of isolates from the same species. This approach was problematic, however, because the nomenclature for some pairs of isolates classifies them as belonging to different species even though their 16S rRNA genes are nearly identical. For example, the 16S rRNA gene of *B. anthracis *strain Sterne is 99.85% identical to that of *Bacillus cereus *strain ATCC 14579. Thus, we instead included pairs of isolates in the analysis only if their 16S rRNA genes were less than 99.5% identical, regardless of their accepted species naming.

To further compare 16S rRNA gene similarity with our two proteomic similarity measures, we generated three phylogenetic trees, each of which was based on a different distance metric. The distance metric used for the first tree was 16S rRNA gene similarity. 16S rRNA gene alignments were created by downloading sequences from the RDP10 website that were pre-aligned based on secondary structure [49]. The evolutionary history was inferred using the maximum likelihood neighbour-joining method [50] within the Molecular Evolutionary Genetics Analysis (MEGA) program [51]. Within MEGA, a bootstrap test with 1000 replicates was used. The second tree used the same metric employed by Snel et al. [13], which is 1 - *S*/*P*, where *S *is the number of shared proteins between two isolates and *P *is the size of the smaller proteome. The metric used for the third tree was simply the average unique proteins measure described above. For the protein-based distance metrics, trees were created using the unweighted pair group method with arithmetic mean (UPGMA). Graphical representations of the complete trees were created using Geneious [52], while those of the collapsed trees were created using MEGA [51].

### Comparing the protein content of selected species

#### Evaluating taxonomic classifications by determining how well species are clustered based on protein content

This section describes an analysis that examines the quality of current taxonomic classifications from a novel perspective--specifically, by determining the level of cohesiveness in the protein content of a given species. This can be conceptualized as a clustering problem. The general idea behind clustering is that each element in a given cluster should be similar to other elements in the same cluster, but dissimilar to elements from other clusters. In the context of taxonomy and protein content, the clustering of a given species could be considered sound if two criteria are satisfied: first, members of the species are similar to each other (i.e. have a large core proteome); second, they are distinct from other organisms (i.e. have many proteins found only in that species). To determine whether existing taxonomic classifications fit these criteria, we answered the following two questions. First, is the core proteome of a particular species having *N*_*I *_sequenced isolates larger than the core proteome of *N*_*I *_randomly selected organisms from the same genus? Second, is the number of proteins that are found in all *N*_*I *_isolates of a given species, but none of the other organisms from the same genus (i.e. unique proteins), larger than the number of proteins found in *N*_*I *_randomly selected isolates of that genus, but no others?

The rationale behind asking these questions is that one would expect the isolates of a given species to have a larger core proteome and unique proteome than randomly selected sets of isolates from the same genus. Thus, a "yes" answer to each of the above questions would support the species' current taxonomic classification. In contrast, "no" answers to one or both questions would suggest that the species does not fit the clustering criteria given above, and its taxonomic classification may therefore warrant reexamination. The following describes only the methodology used to address the first question; however, the methodology used to answer the second question was analogous, and is briefly described in the final paragraph of this section. Once again, let *N*_*I *_be the number of isolates that have been sequenced for a particular species *S*. The following methodology was performed for each species from the genera used in this study that had at least two isolates sequenced. First, a set of *N*_*I *_isolates from the same genus as *S *was randomly selected. Each random isolate was allowed to be from any species from the same genus as *S*; they were not limited to the species meeting the "at least two isolates sequenced" requirement. This set was examined to ensure that its members were not all from the same species. For instance, when generating random sets of two organisms each corresponding to the two *B. thuringiensis *isolates (*N*_*I *_= 2), a random set containing both *B. thuringiensis *isolates would have been disallowed, as would a random set containing two *B. anthracis *isolates. However, a random set containing one *B. thuringiensis *isolate and one *B. anthracis *would have been valid. If a random set was generated, but all of its members were from the same species, then the set was discarded and another generated in its place. The size of the core proteome of this set of organisms was then determined. This procedure was then repeated 24 more times; in other words, 25 random sets of *N*_*I *_organisms were constructed, and the size of the core proteome was determined for each. The 25 sets were also checked to ensure that none of the sets were the same. The reasons for choosing 25 random sets, rather than some other quantity, were: (a) this number is large enough to make the results statistically meaningful, and (b) this number is not much larger than the maximum number of random sets that could be generated for some species.

As just mentioned, some genera had too few sequenced isolates to enable 25 sets to be created. For instance, the genus *Neisseria *had only six isolates sequenced in total, with two *Neisseria gonorrhoeae *isolates and four *Neisseria meningitidis *isolates. When generating random sets corresponding to *N. gonorrhoeae*, the number of possible ways to choose two items from six is C(6, 2) = 15. However, seven of these sets had both organisms from the same species, leaving just eight valid sets. Similarly, in generating random sets corresponding to *N. meningitidis*, the number of ways in which one can choose four items from six is the same: C(6, 4) = 15. One of these sets (the one containing all four *N. meningitidis *isolates) was invalid, leaving 14 sets. Besides these two *Neisseria *species, other species for which fewer than 25 sets could be constructed were *Brucella suis *(24 sets), *R. leguminosarum *(4 sets), *R. etli *(4 sets), and *Shigella boydii *(17 sets). These species were analyzed in the same manner as the others, but with statistical tests (see below) taking into account the smaller sample sizes.

After finding the core proteome sizes of all 25 (or fewer for the aforementioned species) random sets for a given species, a t-test was performed to determine whether the mean of the core proteome sizes for the randomly-generated sets was different than the core proteome size of the *N*_*I *_isolates of the species in question.

The approach to the second question was analogous to the procedure given above, except that rather than finding proteins that are found in all members of a given set of organisms, proteins were found that exist in all members of a given set, *and *in no other organisms from the same genus.

## Authors' contributions

BT participated in the design and coordination of the study, developed and implemented the necessary software, performed computational analyses, and drafted parts of the manuscript. MH conceived of the study, participated in the design, performed statistical analyses and biological interpretation, and drafted parts of the manuscript. VP helped to draft the manuscript, assembled data, and provided scientific input regarding biological interpretation. BZ and AK participated in the design and coordination of the study, helped to draft the manuscript, supervised the research, and are holders of research grants used to fund the study. All authors read and approved the final manuscript.

## Supplementary Material

Additional file 1 **Complete list of organisms used**. These tables list the isolates used for each of the genera listed in Table 1 of the main paper. Where it would not lead to ambiguity some strain designations have been removed or shortened to save space. For instance, the full description of the bacterium listed as "*B. thailandensis *E264/ATCC 700388" is actually "*B. thailandensis *(strain E264/ATCC 700388/DSM 13276/CIP 106301)". The name of each organism is accompanied by its taxonomic ID, the number of proteins in its proteome, and its genome size. Click here for file

Additional file 2 **Full phylogenetic tree based on 16S rRNA gene similarity**. 16S rRNA gene alignments were created by downloading sequences from the RDP10 website that were prealigned based on secondary structure. The evolutionary history was inferred using the maximum likelihood neighbor-joining method within the Molecular Evolutionary Genetics Analysis (MEGA) program. Within MEGA, a bootstrap test with 1000 replicates was used. The graphical representation of the tree was created using Geneious. Click here for file

Additional file 3 **Full phylogenetic tree based on shared proteins**. Distances between organisms were calculated using the formula 1 - *S/P*, where *S *is the number of shared proteins between two isolates and *P *is the size of the smaller proteome. The unweighted pair group method with arithmetic mean (UPGMA) was used to create a dendrogram from these distances. The graphical representation of the tree was created using Geneious. Click here for file

Additional file 4 **Full phylogenetic tree based on average unique proteins**. The distance between a given pair of organisms was simply the average unique proteins measure for that pair. The unweighted pair group method with arithmetic mean (UPGMA) was used to create a dendrogram from these distances. The graphical representation of the tree was created using Geneious. Click here for file

Additional file 5 **Complete list of random groups**. These tables list the random groups used for the analysis whose results are summarized in Tables 3 and 4 of the main paper. The column heading **N**_**C **_indicates the number of proteins in that group's core proteome, while **N**_**U **_indicates the number of proteins found in the proteomes of all members of that group, but no other isolates from the same genus. Click here for file
