# Supplementary material for: Analysis and comparison of the pan-genomic properties of sixteen well-characterized bacterial genera
Source: BMC Microbiol. 2010 Oct 13;10:258. doi: 10.1186/1471-2180-10-258 (PMC3020658; doi:10.1186/1471-2180-10-258)
Supplement: Additional file 1 — Complete list of organisms used. These tables list the isolates used for each of the genera listed in Table 1 of the main paper. Where it would not lead to ambiguity some strain designations have been removed or shortened to save space. For instance, the full description of the bacterium listed as "B. thailandensis E264/ATCC 700388" is actually "B. thailandensis (strain E264/ATCC 700388/DSM 13276/CIP 106301)". The name of each organism is accompanied by its taxonomic ID, the number of proteins in its proteome, and its genome size. [file 1471-2180-10-258-S1.ZIP › Bacillus.pdf]

Complete list of *Bacillus* isolates used.

**TaxID**	**Isolate**	**Proteins (#)**	**Genome size (bp)**
326423	*B. amyloliquefaciens* FZB42	3692	3,918,589
261594	*B. anthracis* Ames ancestor	5590	5,227,419
198094	*B. anthracis* Ames, isolate Porton	5313	5,227,293
260799	*B. anthracis* Sterne	5288	5,228,663
222523	*B. cereus* ATCC 10987	5821	5,224,283
226900	*B. cereus* ATCC 14579 / DSM 31	5240	5,411,809
288681	*B. cereus* ZK / E33L	5638	5,300,915
315749	*B. cereus* subsp. cytotoxis, strain NVH 391-98	3840	4,087,024
66692	*B. clausii* KSM-K16	4082	4,303,871
272558	*B. halodurans* C-125 / ATCC BAA-125	4006	4,202,352
279010	*B. licheniformis* DSM 13 / ATCC 14580	4162	4,222,597
315750	*B. pumilus* SAFR-032	3675	3,704,465
224308	*B. subtilis* 168	4112	4,215,606
412694	*B. thuringiensis* Al Hakam	4792	5,257,091
281309	*B. thuringiensis* konkukian, strain 97-27	5169	5,237,682
315730	*B. weihenstephanensis* KBAB4	5650	5,262,775
