# Supplementary material for: Analysis and comparison of the pan-genomic properties of sixteen well-characterized bacterial genera
Source: BMC Microbiol. 2010 Oct 13;10:258. doi: 10.1186/1471-2180-10-258 (PMC3020658; doi:10.1186/1471-2180-10-258)
Supplement: Additional file 1 — Complete list of organisms used. These tables list the isolates used for each of the genera listed in Table 1 of the main paper. Where it would not lead to ambiguity some strain designations have been removed or shortened to save space. For instance, the full description of the bacterium listed as "B. thailandensis E264/ATCC 700388" is actually "B. thailandensis (strain E264/ATCC 700388/DSM 13276/CIP 106301)". The name of each organism is accompanied by its taxonomic ID, the number of proteins in its proteome, and its genome size. [file 1471-2180-10-258-S1.ZIP › Brucella.pdf]

Complete list of *Brucella* isolates used.

**TaxID**	**Isolate**	**Proteins (#)**	**Genome size (bp)**
262698	*B. abortus* biovar 1, strain 9-941	3077	3,286,445
359391	*B. abortus* 2308	3022	3,278,307
430066	*B. abortus* S19	2993	3,283,936
483179	*B. canis* ATCC 23365 / NCTC 10854	3238	3,312,769
224914	*B. melitensis* NCTC 10094 / ATCC 23456 / 16M	3178	3,294,931
444178	*B. ovis* ATCC 25840 / 63/290 / NCTC 10512	2820	3,275,590
204722	*B. suis* biovar 1, strain 1330	3256	3,315,175
470137	*B. suis* ATCC 23445 / NCTC 10510	3214	3,324,607
