# Supplementary material for: Analysis and comparison of the pan-genomic properties of sixteen well-characterized bacterial genera
Source: BMC Microbiol. 2010 Oct 13;10:258. doi: 10.1186/1471-2180-10-258 (PMC3020658; doi:10.1186/1471-2180-10-258)
Supplement: Additional file 1 — Complete list of organisms used. These tables list the isolates used for each of the genera listed in Table 1 of the main paper. Where it would not lead to ambiguity some strain designations have been removed or shortened to save space. For instance, the full description of the bacterium listed as "B. thailandensis E264/ATCC 700388" is actually "B. thailandensis (strain E264/ATCC 700388/DSM 13276/CIP 106301)". The name of each organism is accompanied by its taxonomic ID, the number of proteins in its proteome, and its genome size. [file 1471-2180-10-258-S1.ZIP › Burkholderia.pdf]

Complete list of *Burkholderia* isolates used.

TaxID	Isolate	Proteins (#)	Genome size (bp)
339670	*B. ambifaria* AMMD / ATCC BAA-244	6607	7,484,986
398577	*B. ambifaria* MC40-6	6690	7,340,944
331271	*B. cenocepacia* AU 1054	6450	7,279,116
331272	*B. cenocepacia* HI2424	6898	7,537,983
406425	*B. cenocepacia* MC0-3	6986	7,971,389
216591	*B. cepacia* J2315 / LMG 16656	6993	7,963,121
243160	*B. mallei* ATCC 23344	4797	5,835,527
412022	*B. mallei* NCTC 10229	5309	5,742,303
320389	*B. mallei* NCTC 10247	5619	5,848,380
320388	*B. mallei* SAVP1	4981	5,232,401
391038	*B. phymatum* DSM 17167 / STM815	7461	6,176,561
398527	*B. phytofirmans* DSM 17436 / PsJN	7197	8,093,536
357348	*B. pseudomallei* 1106a	7138	7,089,249
320372	*B. pseudomallei* 1710b	6329	7,308,054
320373	*B. pseudomallei* 668	7215	7,040,403
272560	*B. pseudomallei* K96243	5717	7,247,547
271848	*B. thailandensis* E264 / ATCC 700388	5561	6,723,972
269482	*B. vietnamiensis* R1808 / G4 / LMG 22486	7409	7,305,580
266265	*B. xenovorans* LB400	8591	9,731,138
