# Supplementary material for: Analysis and comparison of the pan-genomic properties of sixteen well-characterized bacterial genera
Source: BMC Microbiol. 2010 Oct 13;10:258. doi: 10.1186/1471-2180-10-258 (PMC3020658; doi:10.1186/1471-2180-10-258)
Supplement: Additional file 1 — Complete list of organisms used. These tables list the isolates used for each of the genera listed in Table 1 of the main paper. Where it would not lead to ambiguity some strain designations have been removed or shortened to save space. For instance, the full description of the bacterium listed as "B. thailandensis E264/ATCC 700388" is actually "B. thailandensis (strain E264/ATCC 700388/DSM 13276/CIP 106301)". The name of each organism is accompanied by its taxonomic ID, the number of proteins in its proteome, and its genome size. [file 1471-2180-10-258-S1.ZIP › Clostridium.pdf]

Complete list of *Clostridium* isolates used.

TaxID	Isolate	Proteins (#)	Genome size (bp)
272562	*C. acetobutylicum* DSM 792 / JCM 1419	3847	3,940,880
290402	*C. beijerinckii* ATCC 51743 / NCIMB 8052	5003	6,000,632
441770	*C. botulinum* ATCC 19397 / Type A	3547	3,863,450
508767	*C. botulinum* Alaska E43 / type E3	3255	3,659,644
508765	*C. botulinum* Eklund 17B / type B	3525	3,800,327
441771	*C. botulinum* ATCC 3502, substrain Los Alamos	3401	3,760,560
413999	*C. botulinum* ATCC 3502, substrain Sanger	3590	3,886,916
441772	*C. botulinum* Langeland / NCTC 10281 / Type F	3657	3,995,387
498214	*C. botulinum* Loch Maree / Type A3	3982	3,992,906
498213	*C. botulinum* Okra / Type B1	3850	3,958,233
272563	*C. difficile* 630	3712	4,290,252
431943	*C. kluyveri* ATCC 8527 / DSM 555	3828	3,964,618
386415	*C. novyi* NT	2305	2,547,720
195102	*C. perfringens* 13 / Type A	2721	3,031,430
195103	*C. perfringens* ATCC 13124 / NCTC 8237	2873	3,256,683
289380	*C. perfringens* SM101 / Type A	2568	2,897,393
357809	*C. phytofermentans* ATCC 700394	3891	4,847,594
212717	*C. tetani* Massachusetts / E88	2414	2,799,251
203119	*C. thermocellum* ATCC 27405 / DSM 1237	3102	3,843,301
