# Supplementary material for: Analysis and comparison of the pan-genomic properties of sixteen well-characterized bacterial genera
Source: BMC Microbiol. 2010 Oct 13;10:258. doi: 10.1186/1471-2180-10-258 (PMC3020658; doi:10.1186/1471-2180-10-258)
Supplement: Additional file 1 — Complete list of organisms used. These tables list the isolates used for each of the genera listed in Table 1 of the main paper. Where it would not lead to ambiguity some strain designations have been removed or shortened to save space. For instance, the full description of the bacterium listed as "B. thailandensis E264/ATCC 700388" is actually "B. thailandensis (strain E264/ATCC 700388/DSM 13276/CIP 106301)". The name of each organism is accompanied by its taxonomic ID, the number of proteins in its proteome, and its genome size. [file 1471-2180-10-258-S1.ZIP › Lactobacillus.pdf]

Complete list of *Lactobacillus* isolates used.

**TaxID**	**Isolate**	**Proteins (#)**	**Genome size (bp)**
272621	*L. acidophilus* NCFM	1859	1,993,560
387344	*L. brevis* ATCC 367 / JCM 1170	2201	2,291,220
321967	*L. casei* ATCC 334	2708	2,895,264
543734	*L. casei* BL23	2999	3,079,196
390333	*L. delbrueckii* ATCC 11842	1519	1,864,998
321956	*L. delbrueckii* ATCC BAA-365	1682	1,856,951
334390	*L. fermentum* IFO 3956 / LMG 18251	1818	2,098,685
324831	*L. gasseri* ATCC 33323 / DSM 20243	1694	1,894,360
405566	*L. helveticus* DPC 4571	1580	2,080,931
257314	*L. johnsonii* NCC 533	1809	1,992,676
220668	*L. plantarum* WCFS1 / ATCC BAA-793	3051	3,308,274
349123	*L. reuteri* 100-23	1972	2,174,299
299033	*L. reuteri* F275	1939	1,999,618
314315	*L. sakei* subsp. sakei, strain 23K	1872	1,884,661
362948	*L. salivarius* subsp. salivarius, strain UCC118	1998	1,827,111
