# Supplementary material for: Analysis and comparison of the pan-genomic properties of sixteen well-characterized bacterial genera
Source: BMC Microbiol. 2010 Oct 13;10:258. doi: 10.1186/1471-2180-10-258 (PMC3020658; doi:10.1186/1471-2180-10-258)
Supplement: Additional file 1 — Complete list of organisms used. These tables list the isolates used for each of the genera listed in Table 1 of the main paper. Where it would not lead to ambiguity some strain designations have been removed or shortened to save space. For instance, the full description of the bacterium listed as "B. thailandensis E264/ATCC 700388" is actually "B. thailandensis (strain E264/ATCC 700388/DSM 13276/CIP 106301)". The name of each organism is accompanied by its taxonomic ID, the number of proteins in its proteome, and its genome size. [file 1471-2180-10-258-S1.ZIP › Mycobacterium.pdf]

Complete list of *Mycobacterium* isolates used.

**TaxID**	**Isolate**	**Proteins (#)**	**Genome size (bp)**
36809	*M. abscessus* ATCC 19977 / DSM 44196	4939	5,067,172
243243	*M. avium* 104	5040	5,475,491
233413	*M. bovis* AF2122/97 / ATCC BAA-935	3911	4,345,492
410289	*M. bovis* BCG / Pasteur 1173P2	3891	4,374,522
350054	*M. gilvum* ATCC 700033 / PYR-GCK	5499	5,619,607
272631	*M. leprae* TN	1603	3,268,203
216594	*M. marinum* ATCC BAA-535 / M	5418	6,636,827
262316	*M. paratuberculosis* ATCC BAA-968 / K-10	4316	4,829,781
246196	*M. smegmatis* ATCC 700084 / mc(2)155)	6597	6,988,209
419947	*M. tuberculosis* ATCC 25177 / H37Ra	3990	6,988,209
83332	*M. tuberculosis* ATCC 25618 / H37Rv	3949	6,988,209
83331	*M. tuberculosis* Oshkosh / CDC 1551	4196	4,403,837
362242	*M. ulcerans* Agy99	4206	5,631,606
350058	*M. vanbaalenii* DSM 7251 / PYR-1	5902	6,491,865
