# Supplementary material for: Analysis and comparison of the pan-genomic properties of sixteen well-characterized bacterial genera
Source: BMC Microbiol. 2010 Oct 13;10:258. doi: 10.1186/1471-2180-10-258 (PMC3020658; doi:10.1186/1471-2180-10-258)
Supplement: Additional file 1 — Complete list of organisms used. These tables list the isolates used for each of the genera listed in Table 1 of the main paper. Where it would not lead to ambiguity some strain designations have been removed or shortened to save space. For instance, the full description of the bacterium listed as "B. thailandensis E264/ATCC 700388" is actually "B. thailandensis (strain E264/ATCC 700388/DSM 13276/CIP 106301)". The name of each organism is accompanied by its taxonomic ID, the number of proteins in its proteome, and its genome size. [file 1471-2180-10-258-S1.ZIP › Neisseria.pdf]

Complete list of *Neisseria* isolates used.

**TaxID**	**Isolate**	**Proteins (#)**	**Genome size (bp)**
242231	*N. gonorrhoeae* ATCC 700825 / FA 1090	1963	2,153,922
521006	*N. gonorrhoeae* NCCP11945	2595	2,232,025
272831	*N. meningitidis* serogroup C, strain ATCC 700532	1865	2,194,961
374833	*N. meningitidis* serogroup C, strain 053442	1998	2,153,416
122587	*N. meningitidis* serogroup A, strain Z2491	1887	2,184,406
122586	*N. meningitidis* serogroup B, strain MC58	2001	2,272,360
