# Supplementary material for: Analysis and comparison of the pan-genomic properties of sixteen well-characterized bacterial genera
Source: BMC Microbiol. 2010 Oct 13;10:258. doi: 10.1186/1471-2180-10-258 (PMC3020658; doi:10.1186/1471-2180-10-258)
Supplement: Additional file 1 — Complete list of organisms used. These tables list the isolates used for each of the genera listed in Table 1 of the main paper. Where it would not lead to ambiguity some strain designations have been removed or shortened to save space. For instance, the full description of the bacterium listed as "B. thailandensis E264/ATCC 700388" is actually "B. thailandensis (strain E264/ATCC 700388/DSM 13276/CIP 106301)". The name of each organism is accompanied by its taxonomic ID, the number of proteins in its proteome, and its genome size. [file 1471-2180-10-258-S1.ZIP › Pseudomonas.pdf]

Complete list of *Pseudomonas* isolates used.

**TaxID**	**Isolate**	**Proteins (#)**	**Genome size (bp)**
208964	*P. aeruginosa* LMG 12228 / ATCC 15692	5558	6,264,404
381754	*P. aeruginosa* PA7	6246	6,588,339
208963	*P. aeruginosa* UCBPP-PA14	5886	6,537,648
384676	*P. entomophila* L48	5126	5,888,780
220664	*P. fluorescens* Pf-5 / ATCC BAA-477	6137	7,074,893
205922	*P. fluorescens* PfO-1	5728	6,438,405
399739	*P. mendocina* ymp	4563	5,072,807
351746	*P. putida* F1 / ATCC 700007	5245	5,959,964
76869	*P. putida* GB-1	5396	6,078,430
160488	*P. putida* KT2440	5313	6,181,863
390235	*P. putida* W619	5179	5,774,330
379731	*P. stutzeri* A1501	4093	4,567,418
264730	*P. syringae* 1448A	5044	5,928,787
205918	*P. syringae* pathovar syringae, strain B728a	5071	6,093,698
223283	*P. syringae* tomato, strain DC3000	5424	6,397,126
