# Supplementary material for: Analysis and comparison of the pan-genomic properties of sixteen well-characterized bacterial genera
Source: BMC Microbiol. 2010 Oct 13;10:258. doi: 10.1186/1471-2180-10-258 (PMC3020658; doi:10.1186/1471-2180-10-258)
Supplement: Additional file 1 — Complete list of organisms used. These tables list the isolates used for each of the genera listed in Table 1 of the main paper. Where it would not lead to ambiguity some strain designations have been removed or shortened to save space. For instance, the full description of the bacterium listed as "B. thailandensis E264/ATCC 700388" is actually "B. thailandensis (strain E264/ATCC 700388/DSM 13276/CIP 106301)". The name of each organism is accompanied by its taxonomic ID, the number of proteins in its proteome, and its genome size. [file 1471-2180-10-258-S1.ZIP › Rhizobium.pdf]

Complete list of *Rhizobium* isolates used.

**TaxID**	**Isolate**	**Proteins (#)**	**Genome size (bp)**
347834	*R. etli* CFN 42 / ATCC 51251	5921	4,381,608
491916	*R. etli* CIAT 652	6050	4,513,324
395492	*R. leguminosarum* bv. trifolii WSM2304	4320	4,537,948
216596	*R. leguminosarum* bv. viciae, strain 3841	7109	5,057,142
