# Supplementary material for: Analysis and comparison of the pan-genomic properties of sixteen well-characterized bacterial genera
Source: BMC Microbiol. 2010 Oct 13;10:258. doi: 10.1186/1471-2180-10-258 (PMC3020658; doi:10.1186/1471-2180-10-258)
Supplement: Additional file 1 — Complete list of organisms used. These tables list the isolates used for each of the genera listed in Table 1 of the main paper. Where it would not lead to ambiguity some strain designations have been removed or shortened to save space. For instance, the full description of the bacterium listed as "B. thailandensis E264/ATCC 700388" is actually "B. thailandensis (strain E264/ATCC 700388/DSM 13276/CIP 106301)". The name of each organism is accompanied by its taxonomic ID, the number of proteins in its proteome, and its genome size. [file 1471-2180-10-258-S1.ZIP › Rickettsia.pdf]

Complete list of *Rickettsia* isolates used.

**TaxID**	**Isolate**	**Proteins (#)**	**Genome size (bp)**
293614	*R. akari* Hartford	1257	1,231,060
391896	*R. bellii* OSU 85-389	1443	1,528,980
336407	*R. bellii* RML369-C	1400	1,522,076
293613	*R. canadensis* McKiel	1091	1,159,772
272944	*R. conorii* ATCC VR-613 / Malish 7	1372	1,268,755
315456	*R. felis* ATCC VR-1525 / URRWXCal2	1428	1,485,148
416276	*R. massiliae* Mtu5	969	1,360,898
272947	*R. prowazekii* Madrid E	834	1,111,523
452659	*R. rickettsii* Iowa	1384	1,268,175
392021	*R. rickettsii* Sheila Smith	1345	1,257,710
257363	*R. typhi* Wilmington / ATCC VR-144	837	1,111,496
