# Supplementary material for: Analysis and comparison of the pan-genomic properties of sixteen well-characterized bacterial genera
Source: BMC Microbiol. 2010 Oct 13;10:258. doi: 10.1186/1471-2180-10-258 (PMC3020658; doi:10.1186/1471-2180-10-258)
Supplement: Additional file 1 — Complete list of organisms used. These tables list the isolates used for each of the genera listed in Table 1 of the main paper. Where it would not lead to ambiguity some strain designations have been removed or shortened to save space. For instance, the full description of the bacterium listed as "B. thailandensis E264/ATCC 700388" is actually "B. thailandensis (strain E264/ATCC 700388/DSM 13276/CIP 106301)". The name of each organism is accompanied by its taxonomic ID, the number of proteins in its proteome, and its genome size. [file 1471-2180-10-258-S1.ZIP › Shigella.pdf]

Complete list of *Shigella* isolates used.

**TaxID**	**Isolate**	**Proteins (#)**	**Genome size (bp)**
344609	*S. boydii* serovar 18, strain CDC 3083-94	4140	4,615,997
300268	*S. boydii* serovar 4, strain Sb227	3937	4,519,823
300267	*S. dysenteriae* serovar 1, strain Sd97 / Sd197	3890	4,369,232
198215	*S. flexneri* serovar 2a, strain ATCC 700930	3786	4,599,354
198214	*S. flexneri* serovar 2a, strain 301	4102	4,607,203
373384	*S. flexneri* serovar 5b, strain 8401	3867	4,574,284
300269	*S. sonnei* Ss046	4053	4,825,265
