# Supplementary material for: Analysis and comparison of the pan-genomic properties of sixteen well-characterized bacterial genera
Source: BMC Microbiol. 2010 Oct 13;10:258. doi: 10.1186/1471-2180-10-258 (PMC3020658; doi:10.1186/1471-2180-10-258)
Supplement: Additional file 1 — Complete list of organisms used. These tables list the isolates used for each of the genera listed in Table 1 of the main paper. Where it would not lead to ambiguity some strain designations have been removed or shortened to save space. For instance, the full description of the bacterium listed as "B. thailandensis E264/ATCC 700388" is actually "B. thailandensis (strain E264/ATCC 700388/DSM 13276/CIP 106301)". The name of each organism is accompanied by its taxonomic ID, the number of proteins in its proteome, and its genome size. [file 1471-2180-10-258-S1.ZIP › Staphylococcus.pdf]

Complete list of *Staphylococcus* isolates used.

**TaxID**	**Isolate**	**Proteins (#)**	**Genome size (bp)**
93062	*S. aureus* COL	2679	2,809,422
359787	*S. aureus* JH1	2761	2,906,507
359786	*S. aureus* JH9	2708	2,906,700
282458	*S. aureus* MRSA252	2639	2,902,619
282459	*S. aureus* MSSA476	2602	2,799,802
196620	*S. aureus* MW2	2660	2,820,462
418127	*S. aureus* Mu3 / ATCC 700698	2684	2,880,168
158878	*S. aureus* Mu50 / ATCC 700699	2714	2,878,529
158879	*S. aureus* N315	2580	2,814,816
93061	*S. aureus* NCTC 8325	2890	2,821,361
426430	*S. aureus* Newman	2578	2,878,897
451516	*S. aureus* USA300 / TCH1516	2688	2,872,915
451515	*S. aureus* USA300	2607	2,872,769
273036	*S. aureus* bovine RF122 / ET3-1 / RF122	2513	2,742,531
176280	*S. epidermidis* ATCC 12228	2461	2,499,279
176279	*S. epidermidis* ATCC 35984 / RP62A	2492	2,616,530
279808	*S. haemolyticus* JCSC1435	2640	2,685,015
342451	*S. saprophyticus* ATCC 15305	2404	2,516,575
