# Supplementary material for: Analysis and comparison of the pan-genomic properties of sixteen well-characterized bacterial genera
Source: BMC Microbiol. 2010 Oct 13;10:258. doi: 10.1186/1471-2180-10-258 (PMC3020658; doi:10.1186/1471-2180-10-258)
Supplement: Additional file 1 — Complete list of organisms used. These tables list the isolates used for each of the genera listed in Table 1 of the main paper. Where it would not lead to ambiguity some strain designations have been removed or shortened to save space. For instance, the full description of the bacterium listed as "B. thailandensis E264/ATCC 700388" is actually "B. thailandensis (strain E264/ATCC 700388/DSM 13276/CIP 106301)". The name of each organism is accompanied by its taxonomic ID, the number of proteins in its proteome, and its genome size. [file 1471-2180-10-258-S1.ZIP › Streptococcus.pdf]

Complete list of *Streptococcus* isolates used.

| TaxID | Isolate | Proteins (#) | Genome size (bp) |
|---|---|---|---|
| 211110 | *S. agalactiae* serovar III, strain NEM316 | 1999 | 2,211,485 |
| 205921 | *S. agalactiae* serovar Ia, strain ATCC 27591 | 1983 | 2,127,839 |
| 208435 | *S. agalactiae* serovar V, strain ATCC BAA-611 | 2105 | 2,160,267 |
| 552526 | *S. equi* MGCS10565 | 1861 | 2,024,171 |
| 467705 | *S. gordonii* ATCC 35105 / CH1 | 2050 | 2,196,662 |
| 210007 | *S. mutans* serovar c, strain ATCC 700610 | 1951 | 2,030,921 |
| 512566 | *S. pneumoniae* serovar 19F, strain G54 | 2106 | 2,078,953 |
| 373153 | *S. pneumoniae* serovar 2, strain NCTC 7466 | 1918 | 2,046,115 |
| 171101 | *S. pneumoniae* ATCC BAA-255 / R6 | 2030 | 2,038,615 |
| 516950 | *S. pneumoniae* CGSP14 | 2193 | 2,209,198 |
| 487214 | *S. pneumoniae* Hungary19A-6 | 2152 | 2,245,615 |
| 170187 | *S. pneumoniae* TIGR4 / ATCC BAA-334 | 2109 | 2,160,842 |
| 370553 | *S. pyogenes* serovar M12, strain MGAS2096 | 1886 | 1,860,355 |
| 370551 | *S. pyogenes* serovar M12, strain MGAS9429 | 1868 | 1,836,467 |
| 370552 | *S. pyogenes* serovar M2, strain MGAS10270 | 1964 | 1,928,252 |
| 370554 | *S. pyogenes* serovar M4, strain MGAS10750 | 1964 | 1,937,111 |
| 160491 | *S. pyogenes* serovar M5, strain Manfredo | 1736 | 1,841,271 |
| 293653 | *S. pyogenes* serovar M1, strain ATCC BAA-947 | 1840 | 1,838,554 |
| 160490 | *S. pyogenes* serovar M1, strain ATCC 700294 | 1691 | 1,852,441 |
| 186103 | *S. pyogenes* serovar M18, strain MGAS8232 | 1835 | 1,895,017 |
| 319701 | *S. pyogenes* serovar M28, strain MGAS6180 | 1884 | 1,897,573 |
| 198466 | *S. pyogenes* serovar M3, strain ATCC BAA-595 | 1858 | 1,900,521 |
| 193567 | *S. pyogenes* serovar M3, strain SSI-1 | 1852 | 1,894,275 |
| 286636 | *S. pyogenes* serovar M6, strain ATCC BAA-946 | 1879 | 1,899,877 |
| 471876 | *S. pyogenes* NZ131 | 1700 | 1,815,785 |
| 388919 | *S. sanguinis* SK36 | 2269 | 2,388,435 |
| 391295 | *S. suis* 05ZYH33 | 2179 | 2,096,309 |
| 391296 | *S. suis* 98HAH33 | 2179 | 2,095,698 |
| 264199 | *S. thermophilus* ATCC BAA-250 / LMG 18311 | 1577 | 1,796,846 |
| 322159 | *S. thermophilus* ATCC BAA-491 / LMD-9 | 1704 | 1,856,368 |
| 299768 | *S. thermophilus* CNRZ 1066 | 1590 | 1,796,226 |
