# Supplementary material for: Analysis and comparison of the pan-genomic properties of sixteen well-characterized bacterial genera
Source: BMC Microbiol. 2010 Oct 13;10:258. doi: 10.1186/1471-2180-10-258 (PMC3020658; doi:10.1186/1471-2180-10-258)
Supplement: Additional file 1 — Complete list of organisms used. These tables list the isolates used for each of the genera listed in Table 1 of the main paper. Where it would not lead to ambiguity some strain designations have been removed or shortened to save space. For instance, the full description of the bacterium listed as "B. thailandensis E264/ATCC 700388" is actually "B. thailandensis (strain E264/ATCC 700388/DSM 13276/CIP 106301)". The name of each organism is accompanied by its taxonomic ID, the number of proteins in its proteome, and its genome size. [file 1471-2180-10-258-S1.ZIP › Vibrio.pdf]

Complete list of *Vibrio* isolates used.

**TaxID**	**Isolate**	**Proteins (#)**	**Genome size (bp)**
243277	*V. cholerae* serovar O1, strain ATCC 39315	3784	4,033,464
345073	*V. cholerae* serovar O1, strain ATCC 39541	3772	4,132,319
312309	*V. fischeri* ATCC 700601 / ES114	3814	4,227,869
388396	*V. fischeri* MJ11	4034	4,323,877
338187	*V. harveyi* ATCC BAA-1116 / BB120	5608	5,969,369
223926	*V. parahaemolyticus* RIMD 2210633	4821	5,165,770
216895	*V. vulnificus* CMCP6	4473	5,126,797
196600	*V. vulnificus* YJ016	4990	5,211,578
