# Supplementary material for: Analysis and comparison of the pan-genomic properties of sixteen well-characterized bacterial genera
Source: BMC Microbiol. 2010 Oct 13;10:258. doi: 10.1186/1471-2180-10-258 (PMC3020658; doi:10.1186/1471-2180-10-258)
Supplement: Additional file 1 — Complete list of organisms used. These tables list the isolates used for each of the genera listed in Table 1 of the main paper. Where it would not lead to ambiguity some strain designations have been removed or shortened to save space. For instance, the full description of the bacterium listed as "B. thailandensis E264/ATCC 700388" is actually "B. thailandensis (strain E264/ATCC 700388/DSM 13276/CIP 106301)". The name of each organism is accompanied by its taxonomic ID, the number of proteins in its proteome, and its genome size. [file 1471-2180-10-258-S1.ZIP › Xanthomonas.pdf]

Complete list of *Xanthomonas* isolates used.

TaxID	Isolate	Proteins (#)	Genome size (bp)
190486	*X. axonopodis* pathovar citri, strain 306	4354	5,175,554
314565	*X. campestris* pathovar campestris, strain 8004	4239	5,148,708
340	*X. campestris* pathovar campestris, strain B100	4410	5,079,002
456327	*X. campestris* pathovar vesicatoria, strain 85-10	4628	5,178,466
190485	*X. campestris* campestris, strain ATCC 33913	4127	5,076,188
342109	*X. oryzae* pathovar oryzae, strain MAFF 311018	4204	4,940,217
360094	*X. oryzae* pathovar oryzae, strain PXO99A	4587	5,240,075
291331	*X. oryzae* oryzae, strain KXO85 / KACC10331	4380	4,941,439
