# Supplementary figures and images for: Analysis and comparison of the pan-genomic properties of sixteen well-characterized bacterial genera
Source: BMC Microbiol. 2010 Oct 13;10:258. doi: 10.1186/1471-2180-10-258 (PMC3020658; doi:10.1186/1471-2180-10-258)

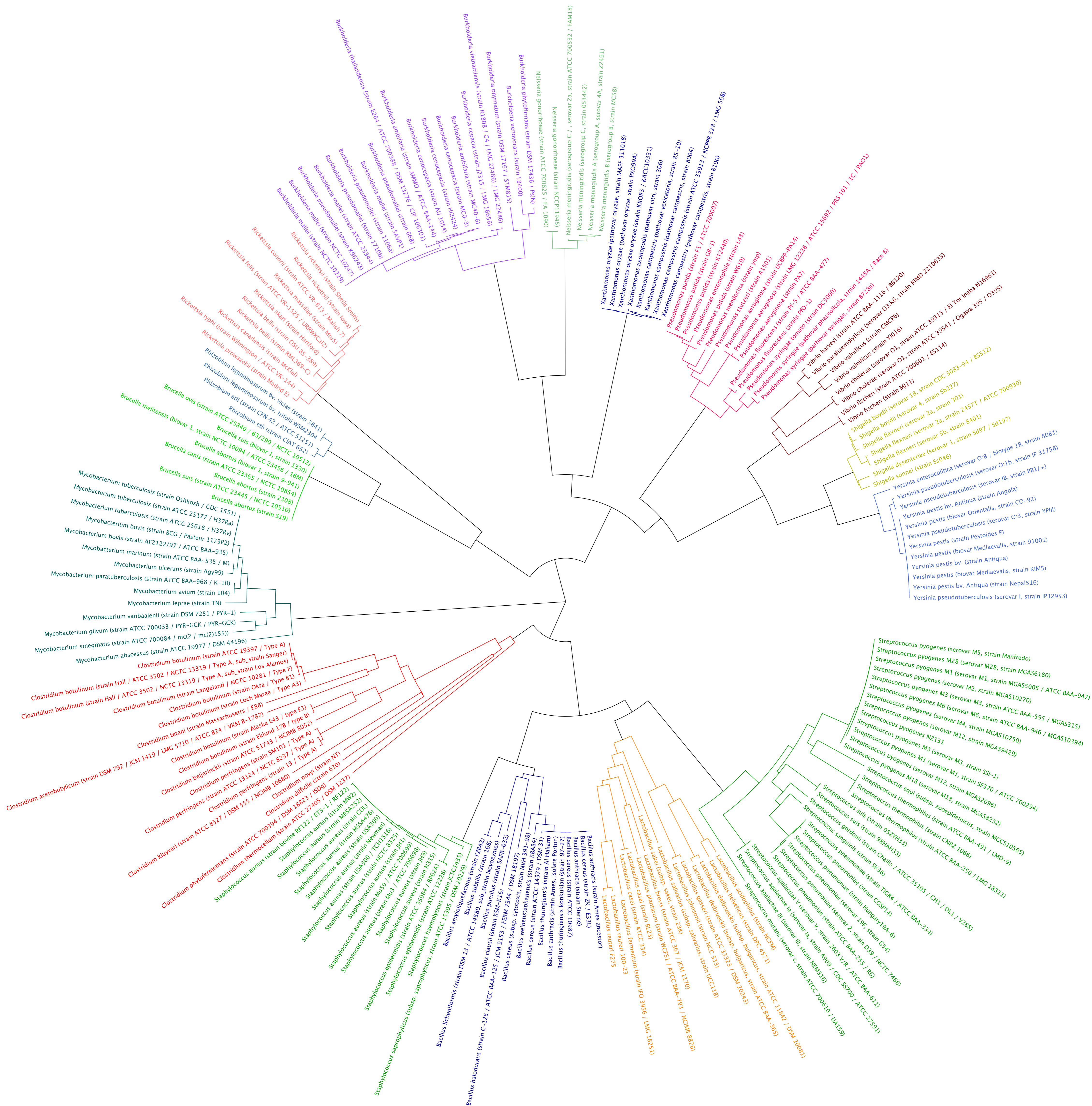

Supplement: Additional file 2 — Full phylogenetic tree based on 16S rRNA gene similarity. 16S rRNA gene alignments were created by downloading sequences from the RDP10 website that were prealigned based on secondary structure. The evolutionary history was inferred using the maximum likelihood neighbor-joining method within the Molecular Evolutionary Genetics Analysis (MEGA) program. Within MEGA, a bootstrap test with 1000 replicates was used. The graphical representation of the tree was created using Geneious. [file 1471-2180-10-258-S2.PDF]

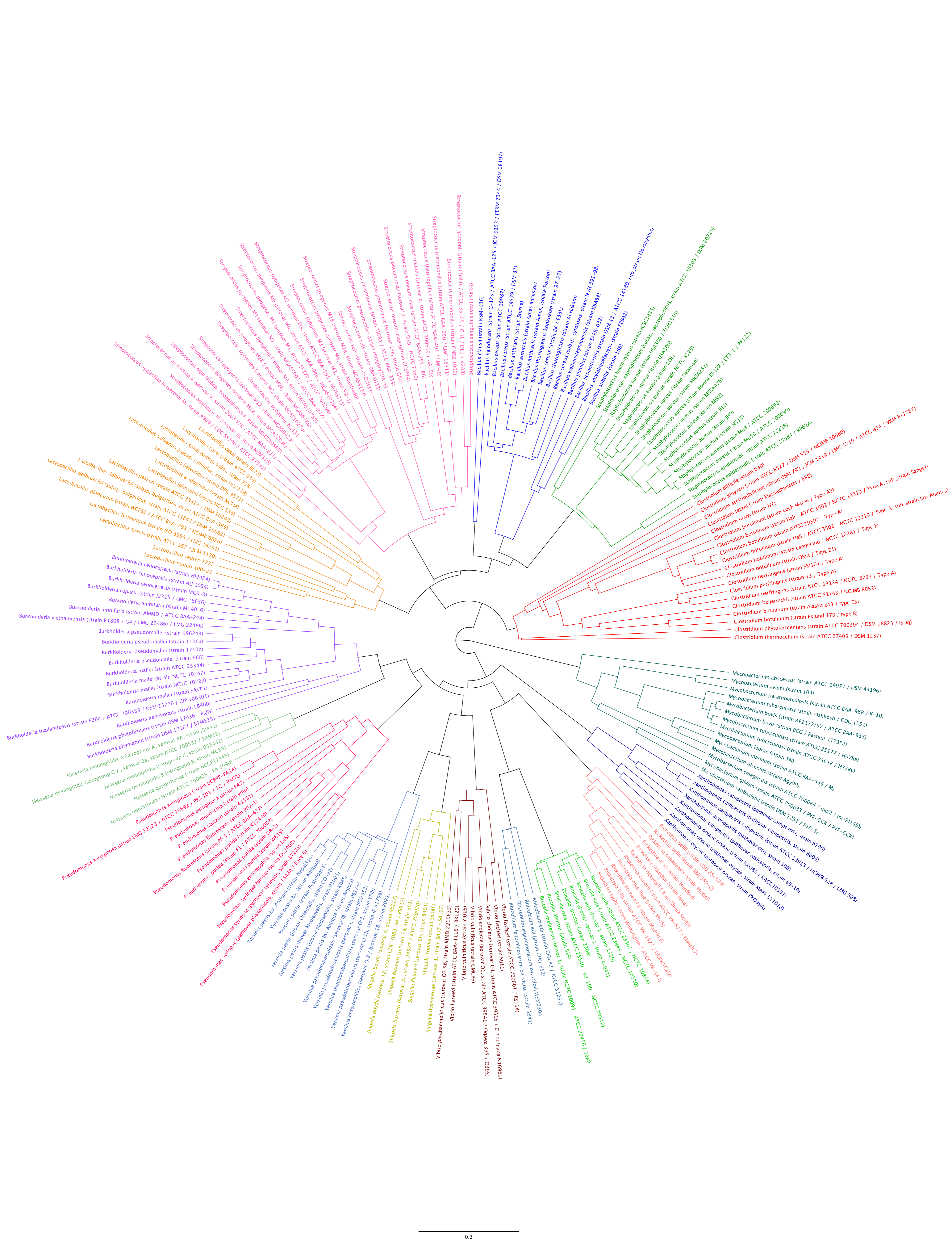

Supplement: Additional file 3 — Full phylogenetic tree based on shared proteins. Distances between organisms were calculated using the formula 1 - S/P, where S is the number of shared proteins between two isolates and P is the size of the smaller proteome. The unweighted pair group method with arithmetic mean (UPGMA) was used to create a dendrogram from these distances. The graphical representation of the tree was created using Geneious. [file 1471-2180-10-258-S3.PDF]

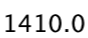

Supplement: Additional file 4 — Full phylogenetic tree based on average unique proteins. The distance between a given pair of organisms was simply the average unique proteins measure for that pair. The unweighted pair group method with arithmetic mean (UPGMA) was used to create a dendrogram from these distances. The graphical representation of the tree was created using Geneious. [file 1471-2180-10-258-S4.PDF]
