# Supplementary material for: Analysis and comparison of the pan-genomic properties of sixteen well-characterized bacterial genera
Source: BMC Microbiol. 2010 Oct 13;10:258. doi: 10.1186/1471-2180-10-258 (PMC3020658; doi:10.1186/1471-2180-10-258)
Supplement: Additional file 5 — Complete list of random groups. These tables list the random groups used for the analysis whose results are summarized in Tables 3 and 4 of the main paper. The column heading NC indicates the number of proteins in that group's core proteome, while NU indicates the number of proteins found in the proteomes of all members of that group, but no other isolates from the same genus. [file 1471-2180-10-258-S5.ZIP › Bacillus_2_isolates.pdf]

Random groups corresponding to *Bacillus* species with 2 isolates.

#	Members of random group	N_C	N_U
1	*B. anthracis* Ames ancestor<br>*B. weihenstephanensis* KBAB4	4204	0
2	*B. amyloliquefaciens* FZB42<br>*B. weihenstephanensis* KBAB4	2153	7
3	*B. amyloliquefaciens* FZB42<br>*B. cereus* subsp. cytotoxis, strain NVH 391-98	1915	1
4	*B. amyloliquefaciens* FZB42<br>*B. halodurans* C-125 / ATCC BAA-125	1904	2
5	*B. anthracis* Ames ancestor<br>*B. licheniformis* DSM 13 / ATCC 14580	2198	1
6	*B. pumilus* SAFR-032<br>*B. halodurans* C-125 / ATCC BAA-125	1921	18
7	*B. thuringiensis* konkukian, strain 97-27<br>*B. weihenstephanensis* KBAB4	4206	6
8	*B. clausii* KSM-K16<br>*B. anthracis* Sterne	1986	0
9	*B. amyloliquefaciens* FZB42<br>*B. thuringiensis* konkukian, strain 97-27	2153	1
10	*B. anthracis* Sterne<br>*B. thuringiensis* Al Hakam	4279	2
11	*B. anthracis* Sterne<br>*B. cereus* subsp. cytotoxis, strain NVH 391-98	3071	0
12	*B. anthracis* Ames ancestor<br>*B. cereus* subsp. cytotoxis, strain NVH 391-98	3036	3
13	*B. amyloliquefaciens* FZB42<br>*B. anthracis* Ames, isolate Porton	2106	0
14	*B. pumilus* SAFR-032<br>*B. thuringiensis* konkukian, strain 97-27	2091	0
15	*B. pumilus* SAFR-032<br>*B. anthracis* Sterne	2077	0
16	*B. cereus* ZK / E33L<br>*B. anthracis* Ames ancestor	4483	1
17	*B. clausii* KSM-K16<br>*B. licheniformis* DSM 13 / ATCC 14580	2119	28
18	*B. clausii* KSM-K16<br>*B. cereus* ATCC 14579 / DSM 31	1922	0
19	*B. anthracis* Ames ancestor<br>*B. cereus* ATCC 14579 / DSM 31	4175	1
20	*B. thuringiensis* konkukian, strain 97-27<br>*B. cereus* ATCC 14579 / DSM 31	4286	17
21	*B. cereus* ZK / E33L<br>*B. weihenstephanensis* KBAB4	4301	36
22	*B. halodurans* C-125 / ATCC BAA-125<br>*B. cereus* ATCC 14579 / DSM 31	1920	3
23	*B. halodurans* C-125 / ATCC BAA-125<br>*B. anthracis* Ames, isolate Porton	1916	0
24	*B. licheniformis* DSM 13 / ATCC 14580<br>*B. subtilis* 168	2914	63
25	*B. cereus* ATCC 10987<br>*B. anthracis* Sterne	4267	0
