# Supplementary material for: Analysis and comparison of the pan-genomic properties of sixteen well-characterized bacterial genera
Source: BMC Microbiol. 2010 Oct 13;10:258. doi: 10.1186/1471-2180-10-258 (PMC3020658; doi:10.1186/1471-2180-10-258)
Supplement: Additional file 5 — Complete list of random groups. These tables list the random groups used for the analysis whose results are summarized in Tables 3 and 4 of the main paper. The column heading NC indicates the number of proteins in that group's core proteome, while NU indicates the number of proteins found in the proteomes of all members of that group, but no other isolates from the same genus. [file 1471-2180-10-258-S5.ZIP › Bacillus_3_isolates.pdf]

Random groups corresponding to *Bacillus* species with 3 isolates.

#	Members of random group	N_C	N_U
1	*B. amyloliquefaciens* FZB42<br>*B. halodurans* C-125 / ATCC BAA-125<br>*B. weihenstephanensis* KBAB4	1629	0
2	*B. pumilus* SAFR-032<br>*B. licheniformis* DSM 13 / ATCC 14580<br>*B. thuringiensis* konkukian, strain 97-27	1922	0
3	*B. amyloliquefaciens* FZB42<br>*B. licheniformis* DSM 13 / ATCC 14580<br>*B. cereus* ATCC 14579 / DSM 31	1980	1
4	*B. licheniformis* DSM 13 / ATCC 14580<br>*B. anthracis* Ames, isolate Porton<br>*B. thuringiensis* konkukian, strain 97-27	2152	0
5	*B. clausii* KSM-K16<br>*B. amyloliquefaciens* FZB42<br>*B. thuringiensis* Al Hakam	1637	1
6	*B. pumilus* SAFR-032<br>*B. cereus* ATCC 10987<br>*B. cereus* subsp. cytotoxis, strain NVH 391-98	1809	0
7	*B. cereus* ATCC 10987<br>*B. cereus* subsp. cytotoxis, strain NVH 391-98<br>*B. weihenstephanensis* KBAB4	2966	6
8	*B. clausii* KSM-K16<br>*B. amyloliquefaciens* FZB42<br>*B. cereus* subsp. cytotoxis, strain NVH 391-98	1556	0
9	*B. clausii* KSM-K16<br>*B. cereus* ATCC 10987<br>*B. anthracis* Sterne	1920	0
10	*B. cereus* ATCC 10987<br>*B. amyloliquefaciens* FZB42<br>*B. halodurans* C-125 / ATCC BAA-125	1634	0
11	*B. cereus* ATCC 10987<br>*B. pumilus* SAFR-032<br>*B. anthracis* Sterne	2021	0
12	*B. anthracis* Ames ancestor<br>*B. subtilis* 168<br>*B. thuringiensis* konkukian, strain 97-27	2122	0
13	*B. cereus* ZK / E33L<br>*B. thuringiensis* Al Hakam<br>*B. thuringiensis* konkukian, strain 97-27	4145	14
14	*B. clausii* KSM-K16<br>*B. pumilus* SAFR-032<br>*B. weihenstephanensis* KBAB4	1623	1
15	*B. anthracis* Sterne<br>*B. weihenstephanensis* KBAB4<br>*B. cereus* ATCC 14579 / DSM 31	3934	0
16	*B. amyloliquefaciens* FZB42<br>*B. cereus* ATCC 14579 / DSM 31<br>*B. thuringiensis* konkukian, strain 97-27	2087	0

17	*B. cereus* ZK / E33L<br>*B. halodurans* C-125 / ATCC BAA-125<br>*B. weihenstephanensis* KBAB4	1918	0
18	*B. cereus* ATCC 10987<br>*B. anthracis* Ames ancestor<br>*B. pumilus* SAFR-032	1986	0
19	*B. cereus* ZK / E33L<br>*B. cereus* subsp. cytotoxis, strain NVH 391-98<br>*B. thuringiensis* konkukian, strain 97-27	3018	2
20	*B. clausii* KSM-K16<br>*B. anthracis* Ames, isolate Porton<br>*B. subtilis* 168	1659	0
21	*B. clausii* KSM-K16<br>*B. halodurans* C-125 / ATCC BAA-125<br>*B. cereus* ATCC 14579 / DSM 31	1674	3
22	*B. cereus* ZK / E33L<br>*B. anthracis* Sterne<br>*B. subtilis* 168	2173	0
23	*B. licheniformis* DSM 13 / ATCC 14580<br>*B. subtilis* 168<br>*B. weihenstephanensis* KBAB4	2041	1
24	*B. cereus* subsp. cytotoxis, strain NVH 391-98<br>*B. halodurans* C-125 / ATCC BAA-125<br>*B. subtilis* 168	1583	0
25	*B. cereus* ZK / E33L<br>*B. amyloliquefaciens* FZB42<br>*B. cereus* subsp. cytotoxis, strain NVH 391-98	1895	1
