# Supplementary material for: Analysis and comparison of the pan-genomic properties of sixteen well-characterized bacterial genera
Source: BMC Microbiol. 2010 Oct 13;10:258. doi: 10.1186/1471-2180-10-258 (PMC3020658; doi:10.1186/1471-2180-10-258)
Supplement: Additional file 5 — Complete list of random groups. These tables list the random groups used for the analysis whose results are summarized in Tables 3 and 4 of the main paper. The column heading NC indicates the number of proteins in that group's core proteome, while NU indicates the number of proteins found in the proteomes of all members of that group, but no other isolates from the same genus. [file 1471-2180-10-258-S5.ZIP › Bacillus_4_isolates.pdf]

Random groups corresponding to *Bacillus* species with 4 isolates.

#	Members of random group	N_C	N_U
1	*B. anthracis* Sterne<br>*B. amyloliquefaciens* FZB42<br>*B. halodurans* C-125 / ATCC BAA-125<br>*B. licheniformis* DSM 13 / ATCC 14580	1603	0
2	*B. clausii* KSM-K16<br>*B. cereus* ATCC 10987<br>*B. subtilis* 168<br>*B. thuringiensis* konkukian, strain 97-27	1637	0
3	*B. clausii* KSM-K16<br>*B. anthracis* Ames ancestor<br>*B. amyloliquefaciens* FZB42<br>*B. anthracis* Ames, isolate Porton	1640	0
4	*B. clausii* KSM-K16<br>*B. anthracis* Sterne<br>*B. amyloliquefaciens* FZB42<br>*B. cereus* ATCC 14579 / DSM 31	1619	0
5	*B. cereus* ATCC 10987<br>*B. amyloliquefaciens* FZB42<br>*B. anthracis* Ames, isolate Porton<br>*B. subtilis* 168	1991	0
6	*B. anthracis* Sterne<br>*B. licheniformis* DSM 13 / ATCC 14580<br>*B. subtilis* 168<br>*B. cereus* ATCC 14579 / DSM 31	1987	0
7	*B. cereus* ZK / E33L<br>*B. halodurans* C-125 / ATCC BAA-125<br>*B. thuringiensis* Al Hakam<br>*B. licheniformis* DSM 13 / ATCC 14580	1671	0
8	*B. pumilus* SAFR-032<br>*B. amyloliquefaciens* FZB42<br>*B. thuringiensis* Al Hakam<br>*B. thuringiensis* konkukian, strain 97-27	1873	0
9	*B. cereus* ATCC 10987<br>*B. amyloliquefaciens* FZB42<br>*B. licheniformis* DSM 13 / ATCC 14580<br>*B. anthracis* Ames, isolate Porton	1926	0
10	*B. clausii* KSM-K16<br>*B. pumilus* SAFR-032<br>*B. anthracis* Sterne<br>*B. weihenstephanensis* KBAB4	1622	0
11	*B. pumilus* SAFR-032<br>*B. cereus* subsp. cytotoxis, strain NVH 391-98<br>*B. thuringiensis* Al Hakam<br>*B. thuringiensis* konkukian, strain 97-27	1771	0
12	*B. pumilus* SAFR-032<br>*B. cereus* ATCC 10987<br>*B. amyloliquefaciens* FZB42<br>*B. thuringiensis* Al Hakam	1850	0

13	*B. cereus* ZK / E33L<br>*B. amyloliquefaciens* FZB42<br>*B. thuringiensis* konkukian, strain 97-27<br>*B. weihenstephanensis* KBAB4	2084	0
14	*B. anthracis* Sterne<br>*B. amyloliquefaciens* FZB42<br>*B. licheniformis* DSM 13 / ATCC 14580<br>*B. anthracis* Ames, isolate Porton	1970	0
15	*B. clausii* KSM-K16<br>*B. anthracis* Sterne<br>*B. licheniformis* DSM 13 / ATCC 14580<br>*B. anthracis* Ames, isolate Porton	1685	0
16	*B. cereus* ATCC 10987<br>*B. anthracis* Ames ancestor<br>*B. cereus* subsp. cytotoxis, strain NVH 391-98<br>*B. thuringiensis* Al Hakam	2730	0
17	*B. amyloliquefaciens* FZB42<br>*B. anthracis* Ames, isolate Porton<br>*B. subtilis* 168<br>*B. weihenstephanensis* KBAB4	2011	0
18	*B. halodurans* C-125 / ATCC BAA-125<br>*B. subtilis* 168<br>*B. weihenstephanensis* KBAB4<br>*B. cereus* ATCC 14579 / DSM 31	1642	0
19	*B. cereus* ZK / E33L<br>*B. amyloliquefaciens* FZB42<br>*B. cereus* subsp. cytotoxis, strain NVH 391-98<br>*B. halodurans* C-125 / ATCC BAA-125	1542	0
20	*B. pumilus* SAFR-032<br>*B. halodurans* C-125 / ATCC BAA-125<br>*B. anthracis* Ames, isolate Porton<br>*B. thuringiensis* konkukian, strain 97-27	1595	0
21	*B. anthracis* Ames ancestor<br>*B. amyloliquefaciens* FZB42<br>*B. cereus* ATCC 14579 / DSM 31<br>*B. weihenstephanensis* KBAB4	2017	0
22	*B. pumilus* SAFR-032<br>*B. cereus* ATCC 10987<br>*B. licheniformis* DSM 13 / ATCC 14580<br>*B. thuringiensis* Al Hakam	1838	0
23	*B. cereus* ATCC 10987<br>*B. anthracis* Sterne<br>*B. licheniformis* DSM 13 / ATCC 14580<br>*B. subtilis* 168	1998	0
24	*B. cereus* ZK / E33L<br>*B. halodurans* C-125 / ATCC BAA-125<br>*B. thuringiensis* konkukian, strain 97-27<br>*B. cereus* ATCC 14579 / DSM 31	1871	0

25	*B. anthracis* Ames ancestor<br>*B. pumilus* SAFR-032<br>*B. amyloliquefaciens* FZB42<br>*B. cereus* ATCC 14579 / DSM 31	1848	0
