# Supplementary material for: Analysis and comparison of the pan-genomic properties of sixteen well-characterized bacterial genera
Source: BMC Microbiol. 2010 Oct 13;10:258. doi: 10.1186/1471-2180-10-258 (PMC3020658; doi:10.1186/1471-2180-10-258)
Supplement: Additional file 5 — Complete list of random groups. These tables list the random groups used for the analysis whose results are summarized in Tables 3 and 4 of the main paper. The column heading NC indicates the number of proteins in that group's core proteome, while NU indicates the number of proteins found in the proteomes of all members of that group, but no other isolates from the same genus. [file 1471-2180-10-258-S5.ZIP › Brucella_2_isolates.pdf]

Random groups corresponding to *Brucella* species with 2 isolates.

#	Members of random group	N_C	N_U
1	*B. suis* biovar 1, strain 1330<br>*B. ovis* ATCC 25840 / 63/290 / NCTC 10512	2584	2
2	*B. suis* biovar 1, strain 1330<br>*B. abortus* S19	2794	0
3	*B. suis* biovar 1, strain 1330<br>*B. melitensis* NCTC 10094 / ATCC 23456 / 16M	2838	1
4	*B. canis* ATCC 23365 / NCTC 10854<br>*B. suis* ATCC 23445 / NCTC 10510	3040	26
5	*B. melitensis* NCTC 10094 / ATCC 23456 / 16M<br>*B. abortus* 2308	2751	10
6	*B. canis* ATCC 23365 / NCTC 10854<br>*B. abortus* S19	2787	0
7	*B. melitensis* NCTC 10094 / ATCC 23456 / 16M<br>*B. abortus* biovar 1, strain 9-941	2754	4
8	*B. suis* biovar 1, strain 1330<br>*B. abortus* 2308	2853	3
9	*B. abortus* biovar 1, strain 9-941<br>*B. canis* ATCC 23365 / NCTC 10854	2839	0
10	*B. suis* biovar 1, strain 1330<br>*B. canis* ATCC 23365 / NCTC 10854	3060	4
11	*B. abortus* biovar 1, strain 9-941<br>*B. suis* ATCC 23445 / NCTC 10510	2827	0
12	*B. abortus* 2308<br>*B. suis* ATCC 23445 / NCTC 10510	2789	0
13	*B. melitensis* NCTC 10094 / ATCC 23456 / 16M<br>*B. suis* ATCC 23445 / NCTC 10510	2823	5
14	*B. abortus* 2308<br>*B. ovis* ATCC 25840 / 63/290 / NCTC 10512	2495	0
15	*B. suis* ATCC 23445 / NCTC 10510<br>*B. ovis* ATCC 25840 / 63/290 / NCTC 10512	2592	3
16	*B. suis* ATCC 23445 / NCTC 10510<br>*B. abortus* S19	2777	1
17	*B. abortus* biovar 1, strain 9-941<br>*B. ovis* ATCC 25840 / 63/290 / NCTC 10512	2511	1
18	*B. abortus* S19<br>*B. ovis* ATCC 25840 / 63/290 / NCTC 10512	2580	0
19	*B. melitensis* NCTC 10094 / ATCC 23456 / 16M<br>*B. abortus* S19	2788	13
20	*B. melitensis* NCTC 10094 / ATCC 23456 / 16M<br>*B. canis* ATCC 23365 / NCTC 10854	2842	3
21	*B. melitensis* NCTC 10094 / ATCC 23456 / 16M<br>*B. ovis* ATCC 25840 / 63/290 / NCTC 10512	2582	7
22	*B. canis* ATCC 23365 / NCTC 10854<br>*B. ovis* ATCC 25840 / 63/290 / NCTC 10512	2596	3
23	*B. abortus* 2308<br>*B. canis* ATCC 23365 / NCTC 10854	2804	0
24	*B. suis* biovar 1, strain 1330<br>*B. abortus* biovar 1, strain 9-941	2910	10
