# Supplementary material for: Analysis and comparison of the pan-genomic properties of sixteen well-characterized bacterial genera
Source: BMC Microbiol. 2010 Oct 13;10:258. doi: 10.1186/1471-2180-10-258 (PMC3020658; doi:10.1186/1471-2180-10-258)
Supplement: Additional file 5 — Complete list of random groups. These tables list the random groups used for the analysis whose results are summarized in Tables 3 and 4 of the main paper. The column heading NC indicates the number of proteins in that group's core proteome, while NU indicates the number of proteins found in the proteomes of all members of that group, but no other isolates from the same genus. [file 1471-2180-10-258-S5.ZIP › Brucella_3_isolates.pdf]

Random groups corresponding to *Brucella* species with 3 isolates.

#	Members of random group	N_C	N_U
1	*B. abortus* 2308<br>*B. canis* ATCC 23365 / NCTC 10854<br>*B. ovis* ATCC 25840 / 63/290 / NCTC 10512	2412	0
2	*B. melitensis* NCTC 10094 / ATCC 23456 / 16M<br>*B. abortus* 2308<br>*B. canis* ATCC 23365 / NCTC 10854	2624	0
3	*B. suis* biovar 1, strain 1330<br>*B. abortus* 2308<br>*B. abortus* S19	2647	1
4	*B. melitensis* NCTC 10094 / ATCC 23456 / 16M<br>*B. suis* ATCC 23445 / NCTC 10510<br>*B. ovis* ATCC 25840 / 63/290 / NCTC 10512	2476	3
5	*B. canis* ATCC 23365 / NCTC 10854<br>*B. suis* ATCC 23445 / NCTC 10510<br>*B. abortus* S19	2687	0
6	*B. melitensis* NCTC 10094 / ATCC 23456 / 16M<br>*B. abortus* 2308<br>*B. abortus* biovar 1, strain 9-941	2692	5
7	*B. melitensis* NCTC 10094 / ATCC 23456 / 16M<br>*B. suis* ATCC 23445 / NCTC 10510<br>*B. abortus* S19	2646	2
8	*B. canis* ATCC 23365 / NCTC 10854<br>*B. suis* ATCC 23445 / NCTC 10510<br>*B. ovis* ATCC 25840 / 63/290 / NCTC 10512	2518	12
9	*B. melitensis* NCTC 10094 / ATCC 23456 / 16M<br>*B. abortus* S19<br>*B. ovis* ATCC 25840 / 63/290 / NCTC 10512	2494	3
10	*B. melitensis* NCTC 10094 / ATCC 23456 / 16M<br>*B. abortus* biovar 1, strain 9-941<br>*B. suis* ATCC 23445 / NCTC 10510	2620	0
11	*B. melitensis* NCTC 10094 / ATCC 23456 / 16M<br>*B. abortus* biovar 1, strain 9-941<br>*B. ovis* ATCC 25840 / 63/290 / NCTC 10512	2424	0
12	*B. canis* ATCC 23365 / NCTC 10854<br>*B. abortus* biovar 1, strain 9-941<br>*B. suis* ATCC 23445 / NCTC 10510	2749	0
13	*B. suis* ATCC 23445 / NCTC 10510<br>*B. abortus* S19<br>*B. ovis* ATCC 25840 / 63/290 / NCTC 10512	2487	1
14	*B. melitensis* NCTC 10094 / ATCC 23456 / 16M<br>*B. canis* ATCC 23365 / NCTC 10854<br>*B. suis* ATCC 23445 / NCTC 10510	2737	1
15	*B. suis* biovar 1, strain 1330<br>*B. melitensis* NCTC 10094 / ATCC 23456 / 16M<br>*B. abortus* biovar 1, strain 9-941	2654	1
16	*B. melitensis* NCTC 10094 / ATCC 23456 / 16M<br>*B. abortus* biovar 1, strain 9-941<br>*B. abortus* S19	2641	0

17	*B. abortus* biovar 1, strain 9-941<br>*B. abortus* S19<br>*B. ovis* ATCC 25840 / 63/290 / NCTC 10512	2480	0
18	*B. abortus* 2308<br>*B. canis* ATCC 23365 / NCTC 10854<br>*B. abortus* S19	2639	1
19	*B. suis* biovar 1, strain 1330<br>*B. abortus* S19<br>*B. ovis* ATCC 25840 / 63/290 / NCTC 10512	2489	0
20	*B. abortus* 2308<br>*B. abortus* biovar 1, strain 9-941<br>*B. suis* ATCC 23445 / NCTC 10510	2727	0
21	*B. abortus* 2308<br>*B. canis* ATCC 23365 / NCTC 10854<br>*B. suis* ATCC 23445 / NCTC 10510	2710	0
22	*B. abortus* 2308<br>*B. suis* ATCC 23445 / NCTC 10510<br>*B. ovis* ATCC 25840 / 63/290 / NCTC 10512	2410	0
23	*B. suis* biovar 1, strain 1330<br>*B. abortus* 2308<br>*B. suis* ATCC 23445 / NCTC 10510	2722	0
24	*B. abortus* biovar 1, strain 9-941<br>*B. canis* ATCC 23365 / NCTC 10854<br>*B. abortus* S19	2654	0
25	*B. abortus* 2308<br>*B. canis* ATCC 23365 / NCTC 10854<br>*B. abortus* biovar 1, strain 9-941	2739	0
