# Supplementary material for: Analysis and comparison of the pan-genomic properties of sixteen well-characterized bacterial genera
Source: BMC Microbiol. 2010 Oct 13;10:258. doi: 10.1186/1471-2180-10-258 (PMC3020658; doi:10.1186/1471-2180-10-258)
Supplement: Additional file 5 — Complete list of random groups. These tables list the random groups used for the analysis whose results are summarized in Tables 3 and 4 of the main paper. The column heading NC indicates the number of proteins in that group's core proteome, while NU indicates the number of proteins found in the proteomes of all members of that group, but no other isolates from the same genus. [file 1471-2180-10-258-S5.ZIP › Burkholderia_2_isolates.pdf]

Random groups corresponding to *Burkholderia* species with 2 isolates.

#	Members of random group	N_C	N_U
1	*B. mallei* ATCC 23344<br>*B. ambifaria* MC40-6	3229	0
2	*B. phytofirmans* DSM 17436 / PsJN<br>*B. pseudomallei* K96243	3385	1
3	*B. cenocepacia* MC0-3<br>*B. thailandensis* E264 / ATCC 700388	3799	7
4	*B. phytofirmans* DSM 17436 / PsJN<br>*B. mallei* SAVP1	2916	0
5	*B. ambifaria* MC40-6<br>*B. cenocepacia* AU 1054	4900	0
6	*B. cenocepacia* HI2424<br>*B. thailandensis* E264 / ATCC 700388	3714	0
7	*B. ambifaria* MC40-6<br>*B. phytofirmans* DSM 17436 / PsJN	3871	9
8	*B. xenovorans* LB400<br>*B. pseudomallei* 668	3349	1
9	*B. cenocepacia* MC0-3<br>*B. mallei* SAVP1	3198	0
10	*B. phytofirmans* DSM 17436 / PsJN<br>*B. vietnamiensis* R1808 / G4 / LMG 22486	3789	21
11	*B. phytofirmans* DSM 17436 / PsJN<br>*B. cenocepacia* AU 1054	3823	0
12	*B. cenocepacia* HI2424<br>*B. vietnamiensis* R1808 / G4 / LMG 22486	4561	5
13	*B. mallei* ATCC 23344<br>*B. pseudomallei* K96243	4094	5
14	*B. phytofirmans* DSM 17436 / PsJN<br>*B. pseudomallei* 1106a	3380	0
15	*B. mallei* NCTC 10247<br>*B. phytofirmans* DSM 17436 / PsJN	3084	0
16	*B. xenovorans* LB400<br>*B. cenocepacia* HI2424	3895	4
17	*B. cenocepacia* MC0-3<br>*B. mallei* NCTC 10229	3418	0
18	*B. cenocepacia* HI2424<br>*B. pseudomallei* K96243	3803	0
19	*B. ambifaria* MC40-6<br>*B. pseudomallei* 668	3828	2
20	*B. cenocepacia* MC0-3<br>*B. cepacia* J2315 / LMG 16656	5623	32
21	*B. phytofirmans* DSM 17436 / PsJN<br>*B. phymatum* DSM 17167 / STM815	4616	177
22	*B. mallei* ATCC 23344<br>*B. phymatum* DSM 17167 / STM815	2881	0
23	*B. xenovorans* LB400<br>*B. phymatum* DSM 17167 / STM815	4537	156
24	*B. phytofirmans* DSM 17436 / PsJN<br>*B. cepacia* J2315 / LMG 16656	3957	29
25	*B. phymatum* DSM 17167 / STM815<br>*B. pseudomallei* 1106a	3309	0
