# Supplementary material for: Analysis and comparison of the pan-genomic properties of sixteen well-characterized bacterial genera
Source: BMC Microbiol. 2010 Oct 13;10:258. doi: 10.1186/1471-2180-10-258 (PMC3020658; doi:10.1186/1471-2180-10-258)
Supplement: Additional file 5 — Complete list of random groups. These tables list the random groups used for the analysis whose results are summarized in Tables 3 and 4 of the main paper. The column heading NC indicates the number of proteins in that group's core proteome, while NU indicates the number of proteins found in the proteomes of all members of that group, but no other isolates from the same genus. [file 1471-2180-10-258-S5.ZIP › Burkholderia_3_isolates.pdf]

Random groups corresponding to *Burkholderia* species with 3 isolates.

#	Members of random group	N_C	N_U
1	*B. mallei* ATCC 23344<br>*B. thailandensis* E264 / ATCC 700388<br>*B. mallei* SAVP1	3324	0
2	*B. ambifaria* MC40-6<br>*B. ambifaria* AMMD / ATCC BAA-244<br>*B. mallei* NCTC 10229	3312	0
3	*B. pseudomallei* 1710b<br>*B. pseudomallei* 668<br>*B. cepacia* J2315 / LMG 16656	3703	0
4	*B. mallei* NCTC 10247<br>*B. mallei* SAVP1<br>*B. cenocepacia* AU 1054	3139	0
5	*B. phytofirmans* DSM 17436 / PsJN<br>*B. xenovorans* LB400<br>*B. pseudomallei* 1710b	3145	0
6	*B. mallei* ATCC 23344<br>*B. xenovorans* LB400<br>*B. pseudomallei* 668	2926	0
7	*B. ambifaria* MC40-6<br>*B. vietnamiensis* R1808 / G4 / LMG 22486<br>*B. cepacia* J2315 / LMG 16656	4218	3
8	*B. cenocepacia* MC0-3<br>*B. pseudomallei* 1710b<br>*B. pseudomallei* 1106a	3777	0
9	*B. mallei* NCTC 10229<br>*B. pseudomallei* 1710b<br>*B. phymatum* DSM 17167 / STM815	2912	0
10	*B. ambifaria* MC40-6<br>*B. pseudomallei* 1710b<br>*B. phymatum* DSM 17167 / STM815	3080	0
11	*B. cenocepacia* MC0-3<br>*B. ambifaria* MC40-6<br>*B. xenovorans* LB400	3612	1
12	*B. xenovorans* LB400<br>*B. ambifaria* AMMD / ATCC BAA-244<br>*B. cepacia* J2315 / LMG 16656	3576	2
13	*B. mallei* NCTC 10247<br>*B. ambifaria* AMMD / ATCC BAA-244<br>*B. cenocepacia* AU 1054	3266	0
14	*B. xenovorans* LB400<br>*B. mallei* NCTC 10229<br>*B. phymatum* DSM 17167 / STM815	2798	0
15	*B. cenocepacia* HI2424<br>*B. mallei* SAVP1<br>*B. cenocepacia* AU 1054	3143	0
16	*B. cenocepacia* MC0-3<br>*B. cenocepacia* HI2424<br>*B. mallei* SAVP1	3167	0

17	*B. phytofirmans* DSM 17436 / PsJN<br>*B. mallei* NCTC 10229<br>*B. vietnamiensis* R1808 / G4 / LMG 22486	2866	0
18	*B. phytofirmans* DSM 17436 / PsJN<br>*B. ambifaria* AMMD / ATCC BAA-244<br>*B. cenocepacia* AU 1054	3622	0
19	*B. mallei* ATCC 23344<br>*B. pseudomallei* 1106a<br>*B. vietnamiensis* R1808 / G4 / LMG 22486	3114	0
20	*B. thailandensis* E264 / ATCC 700388<br>*B. pseudomallei* 1710b<br>*B. pseudomallei* K96243	4448	1
21	*B. mallei* NCTC 10247<br>*B. phytofirmans* DSM 17436 / PsJN<br>*B. phymatum* DSM 17167 / STM815	2863	0
22	*B. ambifaria* MC40-6<br>*B. ambifaria* AMMD / ATCC BAA-244<br>*B. pseudomallei* 1710b	3672	0
23	*B. ambifaria* AMMD / ATCC BAA-244<br>*B. mallei* SAVP1<br>*B. pseudomallei* 668	3163	0
24	*B. mallei* NCTC 10247<br>*B. ambifaria* MC40-6<br>*B. pseudomallei* 1106a	3405	0
25	*B. cenocepacia* MC0-3<br>*B. phytofirmans* DSM 17436 / PsJN<br>*B. vietnamiensis* R1808 / G4 / LMG 22486	3558	2
