# Supplementary material for: Analysis and comparison of the pan-genomic properties of sixteen well-characterized bacterial genera
Source: BMC Microbiol. 2010 Oct 13;10:258. doi: 10.1186/1471-2180-10-258 (PMC3020658; doi:10.1186/1471-2180-10-258)
Supplement: Additional file 5 — Complete list of random groups. These tables list the random groups used for the analysis whose results are summarized in Tables 3 and 4 of the main paper. The column heading NC indicates the number of proteins in that group's core proteome, while NU indicates the number of proteins found in the proteomes of all members of that group, but no other isolates from the same genus. [file 1471-2180-10-258-S5.ZIP › Burkholderia_4_isolates.pdf]

Random groups corresponding to *Burkholderia* species with 4 isolates.

#	Members of random group	N_C	N_U
1	*B. mallei* ATCC 23344<br>*B. phytofirmans* DSM 17436 / PsJN<br>*B. mallei* NCTC 10229<br>*B. pseudomallei* K96243	2877	0
2	*B. xenovorans* LB400<br>*B. vietnamiensis* R1808 / G4 / LMG 22486<br>*B. pseudomallei* 1106a<br>*B. pseudomallei* 668	3105	0
3	*B. mallei* NCTC 10247<br>*B. cenocepacia* HI2424<br>*B. mallei* SAVP1<br>*B. pseudomallei* 1106a	3174	0
4	*B. mallei* ATCC 23344<br>*B. phytofirmans* DSM 17436 / PsJN<br>*B. ambifaria* AMMD / ATCC BAA-244<br>*B. thailandensis* E264 / ATCC 700388	2767	0
5	*B. mallei* NCTC 10247<br>*B. xenovorans* LB400<br>*B. vietnamiensis* R1808 / G4 / LMG 22486<br>*B. pseudomallei* 668	2874	0
6	*B. mallei* NCTC 10247<br>*B. cenocepacia* MC0-3<br>*B. thailandensis* E264 / ATCC 700388<br>*B. cenocepacia* AU 1054	3226	0
7	*B. phymatum* DSM 17167 / STM815<br>*B. pseudomallei* 1710b<br>*B. pseudomallei* 1106a<br>*B. cepacia* J2315 / LMG 16656	3025	0
8	*B. xenovorans* LB400<br>*B. cenocepacia* HI2424<br>*B. ambifaria* AMMD / ATCC BAA-244<br>*B. mallei* NCTC 10229	2857	0
9	*B. pseudomallei* 1710b<br>*B. phymatum* DSM 17167 / STM815<br>*B. pseudomallei* K96243<br>*B. pseudomallei* 668	3206	0
10	*B. mallei* ATCC 23344<br>*B. ambifaria* MC40-6<br>*B. thailandensis* E264 / ATCC 700388<br>*B. pseudomallei* 668	3108	0
11	*B. mallei* NCTC 10229<br>*B. phymatum* DSM 17167 / STM815<br>*B. thailandensis* E264 / ATCC 700388<br>*B. cenocepacia* AU 1054	2761	0
12	*B. ambifaria* MC40-6<br>*B. mallei* NCTC 10229<br>*B. pseudomallei* 1710b<br>*B. thailandensis* E264 / ATCC 700388	3174	0

13	*B. cenocepacia* HI2424<br>*B. phymatum* DSM 17167 / STM815<br>*B. thailandensis* E264 / ATCC 700388<br>*B. pseudomallei* 668	2992	0
14	*B. ambifaria* MC40-6<br>*B. thailandensis* E264 / ATCC 700388<br>*B. vietnamiensis* R1808 / G4 / LMG 22486<br>*B. pseudomallei* K96243	3357	0
15	*B. mallei* NCTC 10247<br>*B. mallei* SAVP1<br>*B. cenocepacia* AU 1054<br>*B. cepacia* J2315 / LMG 16656	3066	0
16	*B. mallei* ATCC 23344<br>*B. cenocepacia* MC0-3<br>*B. cenocepacia* HI2424<br>*B. mallei* SAVP1	2972	0
17	*B. mallei* NCTC 10247<br>*B. cenocepacia* MC0-3<br>*B. pseudomallei* 1106a<br>*B. cepacia* J2315 / LMG 16656	3359	0
18	*B. ambifaria* AMMD / ATCC BAA-244<br>*B. pseudomallei* 1106a<br>*B. pseudomallei* 668<br>*B. pseudomallei* K96243	3778	0
19	*B. phytofirmans* DSM 17436 / PsJN<br>*B. xenovorans* LB400<br>*B. mallei* NCTC 10229<br>*B. phymatum* DSM 17167 / STM815	2755	0
20	*B. ambifaria* AMMD / ATCC BAA-244<br>*B. mallei* SAVP1<br>*B. cenocepacia* AU 1054<br>*B. pseudomallei* 1106a	3058	0
21	*B. ambifaria* AMMD / ATCC BAA-244<br>*B. thailandensis* E264 / ATCC 700388<br>*B. pseudomallei* 1106a<br>*B. vietnamiensis* R1808 / G4 / LMG 22486	3341	0
22	*B. mallei* NCTC 10247<br>*B. ambifaria* AMMD / ATCC BAA-244<br>*B. thailandensis* E264 / ATCC 700388<br>*B. pseudomallei* 1106a	3258	0
23	*B. xenovorans* LB400<br>*B. vietnamiensis* R1808 / G4 / LMG 22486<br>*B. pseudomallei* 668<br>*B. pseudomallei* K96243	3094	0
24	*B. mallei* NCTC 10247<br>*B. mallei* ATCC 23344<br>*B. ambifaria* AMMD / ATCC BAA-244<br>*B. cenocepacia* AU 1054	3069	0

25	*B. mallei* NCTC 10247<br>*B. phytofirmans* DSM 17436 / PsJN<br>*B. cenocepacia* AU 1054<br>*B. pseudomallei* 1106a	2912	0
