# Supplementary material for: Analysis and comparison of the pan-genomic properties of sixteen well-characterized bacterial genera
Source: BMC Microbiol. 2010 Oct 13;10:258. doi: 10.1186/1471-2180-10-258 (PMC3020658; doi:10.1186/1471-2180-10-258)
Supplement: Additional file 5 — Complete list of random groups. These tables list the random groups used for the analysis whose results are summarized in Tables 3 and 4 of the main paper. The column heading NC indicates the number of proteins in that group's core proteome, while NU indicates the number of proteins found in the proteomes of all members of that group, but no other isolates from the same genus. [file 1471-2180-10-258-S5.ZIP › Clostridium_3_isolates.pdf]

Random groups corresponding to *Clostridium* species with 3 isolates.

#	Members of random group	N_C	N_U
1	*C. botulinum* Loch Maree / Type A3<br>*C. difficile* 630<br>*C. botulinum* ATCC 3502, substrain Los Alamos	1401	0
2	*C. kluyveri* ATCC 8527 / DSM 555<br>*C. perfringens* 13 / Type A<br>*C. difficile* 630	923	1
3	*C. beijerinckii* ATCC 51743 / NCIMB 8052<br>*C. botulinum* Eklund 17B / type B<br>*C. difficile* 630	1210	4
4	*C. thermocellum* ATCC 27405 / DSM 1237<br>*C. difficile* 630<br>*C. botulinum* Okra / Type B1	896	0
5	*C. kluyveri* ATCC 8527 / DSM 555<br>*C. perfringens* 13 / Type A<br>*C. botulinum* Okra / Type B1	1094	0
6	*C. thermocellum* ATCC 27405 / DSM 1237<br>*C. botulinum* ATCC 19397 / Type A<br>*C. tetani* Massachusetts / E88	879	0
7	*C. thermocellum* ATCC 27405 / DSM 1237<br>*C. botulinum* ATCC 19397 / Type A<br>*C. novyi* NT	924	0
8	*C. botulinum* Loch Maree / Type A3<br>*C. botulinum* Okra / Type B1<br>*C. difficile* 630	1409	0
9	*C. acetobutylicum* DSM 792 / JCM 1419<br>*C. botulinum* Okra / Type B1<br>*C. phytofermentans* ATCC 700394	1009	0
10	*C. thermocellum* ATCC 27405 / DSM 1237<br>*C. tetani* Massachusetts / E88<br>*C. perfringens* ATCC 13124 / NCTC 8237	785	0
11	*C. botulinum* Eklund 17B / type B<br>*C. acetobutylicum* DSM 792 / JCM 1419<br>*C. novyi* NT	1158	1
12	*C. kluyveri* ATCC 8527 / DSM 555<br>*C. phytofermentans* ATCC 700394<br>*C. perfringens* ATCC 13124 / NCTC 8237	864	0
13	*C. botulinum* ATCC 3502, substrain Sanger<br>*C. beijerinckii* ATCC 51743 / NCIMB 8052<br>*C. acetobutylicum* DSM 792 / JCM 1419	1390	0
14	*C. kluyveri* ATCC 8527 / DSM 555<br>*C. botulinum* Alaska E43 / type E3<br>*C. botulinum* ATCC 19397 / Type A	1222	0
15	*C. phytofermentans* ATCC 700394<br>*C. perfringens* 13 / Type A<br>*C. tetani* Massachusetts / E88	830	0
16	*C. botulinum* Eklund 17B / type B<br>*C. botulinum* Alaska E43 / type E3<br>*C. difficile* 630	1276	12

17	*C. botulinum* Loch Maree / Type A3<br>*C. beijerinckii* ATCC 51743 / NCIMB 8052<br>*C. thermocellum* ATCC 27405 / DSM 1237	989	0
18	*C. botulinum* Loch Maree / Type A3<br>*C. tetani* Massachusetts / E88<br>*C. perfringens* ATCC 13124 / NCTC 8237	1076	0
19	*C. kluyveri* ATCC 8527 / DSM 555<br>*C. beijerinckii* ATCC 51743 / NCIMB 8052<br>*C. botulinum* Langeland / NCTC 10281 / Type F	1344	0
20	*C. difficile* 630<br>*C. perfringens* 13 / Type A<br>*C. botulinum* ATCC 3502, substrain Los Alamos	1021	0
21	*C. botulinum* Alaska E43 / type E3<br>*C. difficile* 630<br>*C. perfringens* 13 / Type A	1056	0
22	*C. perfringens* 13 / Type A<br>*C. phytofermentans* ATCC 700394<br>*C. perfringens* SM101 / Type A	1039	0
23	*C. kluyveri* ATCC 8527 / DSM 555<br>*C. beijerinckii* ATCC 51743 / NCIMB 8052<br>*C. perfringens* SM101 / Type A	1069	0
24	*C. botulinum* ATCC 3502, substrain Sanger<br>*C. novyi* NT<br>*C. perfringens* SM101 / Type A	1090	0
25	*C. kluyveri* ATCC 8527 / DSM 555<br>*C. acetobutylicum* DSM 792 / JCM 1419<br>*C. novyi* NT	1187	2
