# Supplementary material for: Analysis and comparison of the pan-genomic properties of sixteen well-characterized bacterial genera
Source: BMC Microbiol. 2010 Oct 13;10:258. doi: 10.1186/1471-2180-10-258 (PMC3020658; doi:10.1186/1471-2180-10-258)
Supplement: Additional file 5 — Complete list of random groups. These tables list the random groups used for the analysis whose results are summarized in Tables 3 and 4 of the main paper. The column heading NC indicates the number of proteins in that group's core proteome, while NU indicates the number of proteins found in the proteomes of all members of that group, but no other isolates from the same genus. [file 1471-2180-10-258-S5.ZIP › Clostridium_8_isolates.pdf]

Random groups corresponding to *Clostridium* species with 8 isolates.

#	Members of random group	N_C	N_U
1	*C. botulinum* ATCC 3502, substrain Sanger<br>*C. botulinum* Loch Maree / Type A3<br>*C. thermocellum* ATCC 27405 / DSM 1237<br>*C. botulinum* Alaska E43 / type E3<br>*C. phytofermentans* ATCC 700394<br>*C. difficile* 630<br>*C. perfringens* 13 / Type A<br>*C. tetani* Massachusetts / E88	615	0
2	*C. botulinum* Alaska E43 / type E3<br>*C. perfringens* ATCC 13124 / NCTC 8237<br>*C. perfringens* SM101 / Type A<br>*C. botulinum* Eklund 17B / type B<br>*C. beijerinckii* ATCC 51743 / NCIMB 8052<br>*C. acetobutylicum* DSM 792 / JCM 1419<br>*C. phytofermentans* ATCC 700394<br>*C. botulinum* ATCC 3502, substrain Los Alamos	773	0
3	*C. botulinum* Langeland / NCTC 10281 / Type F<br>*C. novyi* NT<br>*C. perfringens* ATCC 13124 / NCTC 8237<br>*C. perfringens* SM101 / Type A<br>*C. beijerinckii* ATCC 51743 / NCIMB 8052<br>*C. perfringens* 13 / Type A<br>*C. difficile* 630<br>*C. botulinum* ATCC 3502, substrain Los Alamos	822	0
4	*C. kluyveri* ATCC 8527 / DSM 555<br>*C. botulinum* Langeland / NCTC 10281 / Type F<br>*C. novyi* NT<br>*C. botulinum* ATCC 19397 / Type A<br>*C. tetani* Massachusetts / E88<br>*C. perfringens* ATCC 13124 / NCTC 8237<br>*C. perfringens* 13 / Type A<br>*C. phytofermentans* ATCC 700394	693	0
5	*C. botulinum* Alaska E43 / type E3<br>*C. botulinum* Langeland / NCTC 10281 / Type F<br>*C. tetani* Massachusetts / E88<br>*C. botulinum* ATCC 19397 / Type A<br>*C. botulinum* Loch Maree / Type A3<br>*C. beijerinckii* ATCC 51743 / NCIMB 8052<br>*C. difficile* 630<br>*C. botulinum* ATCC 3502, substrain Los Alamos	878	0
6	*C. botulinum* Okra / Type B1<br>*C. tetani* Massachusetts / E88<br>*C. botulinum* ATCC 19397 / Type A<br>*C. perfringens* ATCC 13124 / NCTC 8237<br>*C. perfringens* SM101 / Type A<br>*C. acetobutylicum* DSM 792 / JCM 1419<br>*C. perfringens* 13 / Type A<br>*C. phytofermentans* ATCC 700394	709	0

7	*C. botulinum* ATCC 3502, substrain Sanger<br>*C. botulinum* Okra / Type B1<br>*C. novyi* NT<br>*C. botulinum* ATCC 19397 / Type A<br>*C. perfringens* SM101 / Type A<br>*C. acetobutylicum* DSM 792 / JCM 1419<br>*C. difficile* 630<br>*C. perfringens* 13 / Type A	809	0
8	*C. novyi* NT<br>*C. tetani* Massachusetts / E88<br>*C. perfringens* ATCC 13124 / NCTC 8237<br>*C. botulinum* Loch Maree / Type A3<br>*C. beijerinckii* ATCC 51743 / NCIMB 8052<br>*C. difficile* 630<br>*C. phytofermentans* ATCC 700394<br>*C. perfringens* 13 / Type A	659	0
9	*C. kluyveri* ATCC 8527 / DSM 555<br>*C. botulinum* Alaska E43 / type E3<br>*C. botulinum* Okra / Type B1<br>*C. botulinum* ATCC 19397 / Type A<br>*C. perfringens* SM101 / Type A<br>*C. botulinum* Loch Maree / Type A3<br>*C. botulinum* Eklund 17B / type B<br>*C. botulinum* ATCC 3502, substrain Los Alamos	984	0
10	*C. botulinum* ATCC 3502, substrain Sanger<br>*C. botulinum* Langeland / NCTC 10281 / Type F<br>*C. botulinum* Okra / Type B1<br>*C. tetani* Massachusetts / E88<br>*C. botulinum* ATCC 19397 / Type A<br>*C. perfringens* ATCC 13124 / NCTC 8237<br>*C. difficile* 630<br>*C. botulinum* ATCC 3502, substrain Los Alamos	858	0
11	*C. botulinum* ATCC 3502, substrain Sanger<br>*C. botulinum* Alaska E43 / type E3<br>*C. novyi* NT<br>*C. tetani* Massachusetts / E88<br>*C. perfringens* ATCC 13124 / NCTC 8237<br>*C. thermocellum* ATCC 27405 / DSM 1237<br>*C. acetobutylicum* DSM 792 / JCM 1419<br>*C. phytofermentans* ATCC 700394	603	0
12	*C. botulinum* ATCC 3502, substrain Sanger<br>*C. botulinum* Alaska E43 / type E3<br>*C. botulinum* Langeland / NCTC 10281 / Type F<br>*C. botulinum* ATCC 19397 / Type A<br>*C. botulinum* Loch Maree / Type A3<br>*C. beijerinckii* ATCC 51743 / NCIMB 8052<br>*C. difficile* 630<br>*C. botulinum* ATCC 3502, substrain Los Alamos	1045	0

13	*C. kluyveri* ATCC 8527 / DSM 555<br>*C. botulinum* Alaska E43 / type E3<br>*C. botulinum* Langeland / NCTC 10281 / Type F<br>*C. novyi* NT<br>*C. botulinum* Eklund 17B / type B<br>*C. thermocellum* ATCC 27405 / DSM 1237<br>*C. acetobutylicum* DSM 792 / JCM 1419<br>*C. perfringens* 13 / Type A	720	0
14	*C. botulinum* ATCC 3502, substrain Sanger<br>*C. botulinum* Langeland / NCTC 10281 / Type F<br>*C. botulinum* Okra / Type B1<br>*C. tetani* Massachusetts / E88<br>*C. perfringens* ATCC 13124 / NCTC 8237<br>*C. beijerinckii* ATCC 51743 / NCIMB 8052<br>*C. phytofermentans* ATCC 700394<br>*C. perfringens* 13 / Type A	755	0
15	*C. kluyveri* ATCC 8527 / DSM 555<br>*C. botulinum* ATCC 19397 / Type A<br>*C. novyi* NT<br>*C. perfringens* SM101 / Type A<br>*C. beijerinckii* ATCC 51743 / NCIMB 8052<br>*C. thermocellum* ATCC 27405 / DSM 1237<br>*C. difficile* 630<br>*C. botulinum* ATCC 3502, substrain Los Alamos	664	0
16	*C. botulinum* ATCC 3502, substrain Sanger<br>*C. kluyveri* ATCC 8527 / DSM 555<br>*C. tetani* Massachusetts / E88<br>*C. novyi* NT<br>*C. perfringens* ATCC 13124 / NCTC 8237<br>*C. botulinum* Eklund 17B / type B<br>*C. beijerinckii* ATCC 51743 / NCIMB 8052<br>*C. difficile* 630	717	0
17	*C. botulinum* ATCC 3502, substrain Sanger<br>*C. botulinum* Okra / Type B1<br>*C. perfringens* ATCC 13124 / NCTC 8237<br>*C. botulinum* Loch Maree / Type A3<br>*C. botulinum* Eklund 17B / type B<br>*C. beijerinckii* ATCC 51743 / NCIMB 8052<br>*C. thermocellum* ATCC 27405 / DSM 1237<br>*C. acetobutylicum* DSM 792 / JCM 1419	775	0
18	*C. botulinum* Alaska E43 / type E3<br>*C. botulinum* Langeland / NCTC 10281 / Type F<br>*C. perfringens* ATCC 13124 / NCTC 8237<br>*C. perfringens* SM101 / Type A<br>*C. botulinum* Loch Maree / Type A3<br>*C. thermocellum* ATCC 27405 / DSM 1237<br>*C. phytofermentans* ATCC 700394<br>*C. botulinum* ATCC 3502, substrain Los Alamos	699	0

19	*C. botulinum* ATCC 3502, substrain Sanger<br>*C. kluyveri* ATCC 8527 / DSM 555<br>*C. botulinum* ATCC 19397 / Type A<br>*C. novyi* NT<br>*C. botulinum* Loch Maree / Type A3<br>*C. botulinum* Eklund 17B / type B<br>*C. thermocellum* ATCC 27405 / DSM 1237<br>*C. botulinum* ATCC 3502, substrain Los Alamos	803	0
20	*C. botulinum* Alaska E43 / type E3<br>*C. tetani* Massachusetts / E88<br>*C. perfringens* SM101 / Type A<br>*C. botulinum* Loch Maree / Type A3<br>*C. beijerinckii* ATCC 51743 / NCIMB 8052<br>*C. acetobutylicum* DSM 792 / JCM 1419<br>*C. perfringens* 13 / Type A<br>*C. botulinum* ATCC 3502, substrain Los Alamos	872	0
21	*C. botulinum* ATCC 3502, substrain Sanger<br>*C. kluyveri* ATCC 8527 / DSM 555<br>*C. botulinum* Langeland / NCTC 10281 / Type F<br>*C. novyi* NT<br>*C. beijerinckii* ATCC 51743 / NCIMB 8052<br>*C. botulinum* Eklund 17B / type B<br>*C. difficile* 630<br>*C. perfringens* 13 / Type A	790	0
22	*C. botulinum* Langeland / NCTC 10281 / Type F<br>*C. botulinum* ATCC 19397 / Type A<br>*C. novyi* NT<br>*C. tetani* Massachusetts / E88<br>*C. perfringens* SM101 / Type A<br>*C. perfringens* ATCC 13124 / NCTC 8237<br>*C. difficile* 630<br>*C. perfringens* 13 / Type A	758	0
23	*C. botulinum* Langeland / NCTC 10281 / Type F<br>*C. novyi* NT<br>*C. perfringens* ATCC 13124 / NCTC 8237<br>*C. botulinum* Eklund 17B / type B<br>*C. thermocellum* ATCC 27405 / DSM 1237<br>*C. difficile* 630<br>*C. phytofermentans* ATCC 700394<br>*C. botulinum* ATCC 3502, substrain Los Alamos	651	0
24	*C. botulinum* Langeland / NCTC 10281 / Type F<br>*C. botulinum* Okra / Type B1<br>*C. tetani* Massachusetts / E88<br>*C. botulinum* ATCC 19397 / Type A<br>*C. perfringens* SM101 / Type A<br>*C. botulinum* Eklund 17B / type B<br>*C. acetobutylicum* DSM 792 / JCM 1419<br>*C. phytofermentans* ATCC 700394	706	0

25	*C. kluyveri* ATCC 8527 / DSM 555<br>*C. botulinum* Alaska E43 / type E3<br>*C. botulinum* Langeland / NCTC 10281 / Type F<br>*C. perfringens* ATCC 13124 / NCTC 8237<br>*C. perfringens* SM101 / Type A<br>*C. thermocellum* ATCC 27405 / DSM 1237<br>*C. acetobutylicum* DSM 792 / JCM 1419<br>*C. botulinum* ATCC 3502, substrain Los Alamos	730	0
