# Supplementary material for: Analysis and comparison of the pan-genomic properties of sixteen well-characterized bacterial genera
Source: BMC Microbiol. 2010 Oct 13;10:258. doi: 10.1186/1471-2180-10-258 (PMC3020658; doi:10.1186/1471-2180-10-258)
Supplement: Additional file 5 — Complete list of random groups. These tables list the random groups used for the analysis whose results are summarized in Tables 3 and 4 of the main paper. The column heading NC indicates the number of proteins in that group's core proteome, while NU indicates the number of proteins found in the proteomes of all members of that group, but no other isolates from the same genus. [file 1471-2180-10-258-S5.ZIP › Lactobacillus_2_isolates.pdf]

Random groups corresponding to *Lactobacillus* species with 2 isolates.

#	Members of random group	N_C	N_U
1	*L. plantarum* WCFS1 / ATCC BAA-793<br>*L. salivarius* subsp. salivarius, strain UCC118	1202	13
2	*L. helveticus* DPC 4571<br>*L. plantarum* WCFS1 / ATCC BAA-793	929	3
3	*L. sakei* subsp. sakei, strain 23K<br>*L. johnsonii* NCC 533	880	0
4	*L. acidophilus* NCFM<br>*L. johnsonii* NCC 533	1252	22
5	*L. helveticus* DPC 4571<br>*L. johnsonii* NCC 533	1058	2
6	*L. fermentum* IFO 3956 / LMG 18251<br>*L. johnsonii* NCC 533	834	1
7	*L. helveticus* DPC 4571<br>*L. delbrueckii* ATCC BAA-365	1001	5
8	*L. brevis* ATCC 367 / JCM 1170<br>*L. gasseri* ATCC 33323 / DSM 20243	862	0
9	*L. brevis* ATCC 367 / JCM 1170<br>*L. salivarius* subsp. salivarius, strain UCC118	1003	4
10	*L. reuteri* 100-23<br>*L. delbrueckii* ATCC 11842	720	1
11	*L. casei* BL23<br>*L. johnsonii* NCC 533	1032	3
12	*L. helveticus* DPC 4571<br>*L. acidophilus* NCFM	1194	56
13	*L. delbrueckii* ATCC 11842<br>*L. casei* ATCC 334	887	0
14	*L. johnsonii* NCC 533<br>*L. reuteri* F275	902	3
15	*L. reuteri* 100-23<br>*L. gasseri* ATCC 33323 / DSM 20243	794	0
16	*L. reuteri* 100-23<br>*L. acidophilus* NCFM	832	3
17	*L. fermentum* IFO 3956 / LMG 18251<br>*L. brevis* ATCC 367 / JCM 1170	1046	10
18	*L. brevis* ATCC 367 / JCM 1170<br>*L. delbrueckii* ATCC 11842	792	0
19	*L. reuteri* F275<br>*L. casei* ATCC 334	1000	0
20	*L. acidophilus* NCFM<br>*L. delbrueckii* ATCC BAA-365	1011	1
21	*L. helveticus* DPC 4571<br>*L. brevis* ATCC 367 / JCM 1170	828	5
22	*L. gasseri* ATCC 33323 / DSM 20243<br>*L. casei* ATCC 334	977	0
23	*L. reuteri* 100-23<br>*L. plantarum* WCFS1 / ATCC BAA-793	1091	4
24	*L. fermentum* IFO 3956 / LMG 18251<br>*L. acidophilus* NCFM	839	0
25	*L. fermentum* IFO 3956 / LMG 18251<br>*L. salivarius* subsp. salivarius, strain UCC118	1009	9
