# Supplementary material for: Analysis and comparison of the pan-genomic properties of sixteen well-characterized bacterial genera
Source: BMC Microbiol. 2010 Oct 13;10:258. doi: 10.1186/1471-2180-10-258 (PMC3020658; doi:10.1186/1471-2180-10-258)
Supplement: Additional file 5 — Complete list of random groups. These tables list the random groups used for the analysis whose results are summarized in Tables 3 and 4 of the main paper. The column heading NC indicates the number of proteins in that group's core proteome, while NU indicates the number of proteins found in the proteomes of all members of that group, but no other isolates from the same genus. [file 1471-2180-10-258-S5.ZIP › Mycobacterium_2_isolates.pdf]

Random groups corresponding to *Mycobacterium* species with 2 isolates.

#	Members of random group	N_C	N_U
1	*M. marinum* ATCC BAA-535 / M<br>*M. avium* 104	3182	9
2	*M. paratuberculosis* ATCC BAA-968 / K-10<br>*M. vanbaalenii* DSM 7251 / PYR-1	2880	2
3	*M. gilvum* ATCC 700033 / PYR-GCK<br>*M. leprae* TN	1295	0
4	*M. marinum* ATCC BAA-535 / M<br>*M. bovis* BCG / Pasteur 1173P2	2949	0
5	*M. avium* 104<br>*M. abscessus* ATCC 19977 / DSM 44196	2420	16
6	*M. bovis* BCG / Pasteur 1173P2<br>*M. abscessus* ATCC 19977 / DSM 44196	2168	0
7	*M. marinum* ATCC BAA-535 / M<br>*M. bovis* AF2122/97 / ATCC BAA-935	2958	0
8	*M. tuberculosis* ATCC 25618 / H37Rv<br>*M. ulcerans* Agy99	2625	0
9	*M. gilvum* ATCC 700033 / PYR-GCK<br>*M. vanbaalenii* DSM 7251 / PYR-1	4327	471
10	*M. tuberculosis* Oshkosh / CDC 1551<br>*M. leprae* TN	1388	0
11	*M. vanbaalenii* DSM 7251 / PYR-1<br>*M. leprae* TN	1309	0
12	*M. smegmatis* ATCC 700084 / mc(2)155)<br>*M. paratuberculosis* ATCC BAA-968 / K-10	2919	7
13	*M. smegmatis* ATCC 700084 / mc(2)155)<br>*M. gilvum* ATCC 700033 / PYR-GCK	3575	55
14	*M. tuberculosis* Oshkosh / CDC 1551<br>*M. vanbaalenii* DSM 7251 / PYR-1	2437	0
15	*M. abscessus* ATCC 19977 / DSM 44196<br>*M. paratuberculosis* ATCC BAA-968 / K-10	2380	5
16	*M. marinum* ATCC BAA-535 / M<br>*M. ulcerans* Agy99	3779	392
17	*M. smegmatis* ATCC 700084 / mc(2)155)<br>*M. leprae* TN	1313	1
18	*M. marinum* ATCC BAA-535 / M<br>*M. paratuberculosis* ATCC BAA-968 / K-10	3148	0
19	*M. abscessus* ATCC 19977 / DSM 44196<br>*M. ulcerans* Agy99	2269	0
20	*M. abscessus* ATCC 19977 / DSM 44196<br>*M. bovis* AF2122/97 / ATCC BAA-935	2171	0
21	*M. smegmatis* ATCC 700084 / mc(2)155)<br>*M. tuberculosis* ATCC 25618 / H37Rv	2438	0
22	*M. tuberculosis* Oshkosh / CDC 1551<br>*M. paratuberculosis* ATCC BAA-968 / K-10	2558	3
23	*M. tuberculosis* ATCC 25177 / H37Ra<br>*M. bovis* AF2122/97 / ATCC BAA-935	3768	0
24	*M. marinum* ATCC BAA-535 / M<br>*M. gilvum* ATCC 700033 / PYR-GCK	2930	8

25	*M. abscessus* ATCC 19977 / DSM 44196<br>*M. leprae* TN	1258	0
