# Supplementary material for: Analysis and comparison of the pan-genomic properties of sixteen well-characterized bacterial genera
Source: BMC Microbiol. 2010 Oct 13;10:258. doi: 10.1186/1471-2180-10-258 (PMC3020658; doi:10.1186/1471-2180-10-258)
Supplement: Additional file 5 — Complete list of random groups. These tables list the random groups used for the analysis whose results are summarized in Tables 3 and 4 of the main paper. The column heading NC indicates the number of proteins in that group's core proteome, while NU indicates the number of proteins found in the proteomes of all members of that group, but no other isolates from the same genus. [file 1471-2180-10-258-S5.ZIP › Mycobacterium_3_isolates.pdf]

Random groups corresponding to *Mycobacterium* species with 3 isolates.

#	Members of random group	N_C	N_U
1	*M. smegmatis* ATCC 700084 / mc(2)155)<br>*M. gilvum* ATCC 700033 / PYR-GCK<br>*M. vanbaalenii* DSM 7251 / PYR-1	3447	357
2	*M. gilvum* ATCC 700033 / PYR-GCK<br>*M. vanbaalenii* DSM 7251 / PYR-1<br>*M. leprae* TN	1297	1
3	*M. avium* 104<br>*M. gilvum* ATCC 700033 / PYR-GCK<br>*M. abscessus* ATCC 19977 / DSM 44196	2166	1
4	*M. avium* 104<br>*M. tuberculosis* Oshkosh / CDC 1551<br>*M. leprae* TN	1310	0
5	*M. avium* 104<br>*M. abscessus* ATCC 19977 / DSM 44196<br>*M. paratuberculosis* ATCC BAA-968 / K-10	2326	30
6	*M. paratuberculosis* ATCC BAA-968 / K-10<br>*M. bovis* AF2122/97 / ATCC BAA-935<br>*M. ulcerans* Agy99	2300	0
7	*M. smegmatis* ATCC 700084 / mc(2)155)<br>*M. avium* 104<br>*M. tuberculosis* ATCC 25177 / H37Ra	2255	0
8	*M. paratuberculosis* ATCC BAA-968 / K-10<br>*M. vanbaalenii* DSM 7251 / PYR-1<br>*M. leprae* TN	1279	0
9	*M. tuberculosis* Oshkosh / CDC 1551<br>*M. bovis* BCG / Pasteur 1173P2<br>*M. bovis* AF2122/97 / ATCC BAA-935	3622	6
10	*M. avium* 104<br>*M. vanbaalenii* DSM 7251 / PYR-1<br>*M. ulcerans* Agy99	2350	0
11	*M. avium* 104<br>*M. paratuberculosis* ATCC BAA-968 / K-10<br>*M. ulcerans* Agy99	2662	2
12	*M. tuberculosis* Oshkosh / CDC 1551<br>*M. ulcerans* Agy99<br>*M. leprae* TN	1334	0
13	*M. marinum* ATCC BAA-535 / M<br>*M. avium* 104<br>*M. ulcerans* Agy99	2743	8
14	*M. marinum* ATCC BAA-535 / M<br>*M. abscessus* ATCC 19977 / DSM 44196<br>*M. ulcerans* Agy99	2279	32
15	*M. paratuberculosis* ATCC BAA-968 / K-10<br>*M. bovis* AF2122/97 / ATCC BAA-935<br>*M. leprae* TN	1326	0
16	*M. gilvum* ATCC 700033 / PYR-GCK<br>*M. ulcerans* Agy99<br>*M. bovis* AF2122/97 / ATCC BAA-935	2131	0

17	*M. gilvum* ATCC 700033 / PYR-GCK<br>*M. tuberculosis* Oshkosh / CDC 1551<br>*M. ulcerans* Agy99	2133	0
18	*M. gilvum* ATCC 700033 / PYR-GCK<br>*M. tuberculosis* Oshkosh / CDC 1551<br>*M. leprae* TN	1269	0
19	*M. marinum* ATCC BAA-535 / M<br>*M. tuberculosis* ATCC 25618 / H37Rv<br>*M. leprae* TN	1387	0
20	*M. bovis* BCG / Pasteur 1173P2<br>*M. vanbaalenii* DSM 7251 / PYR-1<br>*M. tuberculosis* ATCC 25177 / H37Ra	2430	0
21	*M. abscessus* ATCC 19977 / DSM 44196<br>*M. ulcerans* Agy99<br>*M. leprae* TN	1235	0
22	*M. tuberculosis* Oshkosh / CDC 1551<br>*M. avium* 104<br>*M. tuberculosis* ATCC 25618 / H37Rv	2533	0
23	*M. abscessus* ATCC 19977 / DSM 44196<br>*M. tuberculosis* ATCC 25618 / H37Rv<br>*M. tuberculosis* ATCC 25177 / H37Ra	2173	0
24	*M. gilvum* ATCC 700033 / PYR-GCK<br>*M. tuberculosis* ATCC 25177 / H37Ra<br>*M. bovis* AF2122/97 / ATCC BAA-935	2367	0
25	*M. marinum* ATCC BAA-535 / M<br>*M. bovis* BCG / Pasteur 1173P2<br>*M. ulcerans* Agy99	2604	0
