# Supplementary material for: Analysis and comparison of the pan-genomic properties of sixteen well-characterized bacterial genera
Source: BMC Microbiol. 2010 Oct 13;10:258. doi: 10.1186/1471-2180-10-258 (PMC3020658; doi:10.1186/1471-2180-10-258)
Supplement: Additional file 5 — Complete list of random groups. These tables list the random groups used for the analysis whose results are summarized in Tables 3 and 4 of the main paper. The column heading NC indicates the number of proteins in that group's core proteome, while NU indicates the number of proteins found in the proteomes of all members of that group, but no other isolates from the same genus. [file 1471-2180-10-258-S5.ZIP › Neisseria_2_isolates.pdf]

Random groups corresponding to *Neisseria* species with 2 isolates.

#	**Members of random group**	$\mathbf{N_C}$	$\mathbf{N_U}$
1	*N. gonorrhoeae* ATCC 700825 / FA 1090<br>*N. meningitidis* serogroup C, strain ATCC 700532	1484	0
2	*N. gonorrhoeae* NCCP11945<br>*N. meningitidis* serogroup B, strain MC58	1545	5
3	*N. gonorrhoeae* NCCP11945<br>*N. meningitidis* serogroup C, strain ATCC 700532	1567	3
4	*N. gonorrhoeae* ATCC 700825 / FA 1090<br>*N. meningitidis* serogroup C, strain 053442	1536	7
5	*N. gonorrhoeae* NCCP11945<br>*N. meningitidis* serogroup C, strain 053442	1607	9
6	*N. gonorrhoeae* ATCC 700825 / FA 1090<br>*N. meningitidis* serogroup B, strain MC58	1474	2
7	*N. gonorrhoeae* NCCP11945<br>*N. meningitidis* serogroup A, strain Z2491	1547	1
8	*N. meningitidis* serogroup A, strain Z2491<br>*N. gonorrhoeae* ATCC 700825 / FA 1090	1470	0
