# Supplementary material for: Analysis and comparison of the pan-genomic properties of sixteen well-characterized bacterial genera
Source: BMC Microbiol. 2010 Oct 13;10:258. doi: 10.1186/1471-2180-10-258 (PMC3020658; doi:10.1186/1471-2180-10-258)
Supplement: Additional file 5 — Complete list of random groups. These tables list the random groups used for the analysis whose results are summarized in Tables 3 and 4 of the main paper. The column heading NC indicates the number of proteins in that group's core proteome, while NU indicates the number of proteins found in the proteomes of all members of that group, but no other isolates from the same genus. [file 1471-2180-10-258-S5.ZIP › Neisseria_4_isolates.pdf]

Random groups corresponding to *Neisseria* species with 4 isolates.

#	Members of random group	N_C	N_U
1	*N. gonorrhoeae* NCCP11945<br>*N. gonorrhoeae* ATCC 700825 / FA 1090<br>*N. meningitidis* serogroup C, strain ATCC 700532<br>*N. meningitidis* serogroup B, strain MC58	1403	8
2	*N. meningitidis* serogroup A, strain Z2491<br>*N. gonorrhoeae* NCCP11945<br>*N. meningitidis* serogroup C, strain 053442<br>*N. meningitidis* serogroup C, strain ATCC 700532	1491	8
3	*N. meningitidis* serogroup A, strain Z2491<br>*N. gonorrhoeae* NCCP11945<br>*N. gonorrhoeae* ATCC 700825 / FA 1090<br>*N. meningitidis* serogroup B, strain MC58	1401	5
4	*N. meningitidis* serogroup A, strain Z2491<br>*N. gonorrhoeae* NCCP11945<br>*N. meningitidis* serogroup C, strain ATCC 700532<br>*N. meningitidis* serogroup B, strain MC58	1469	8
5	*N. gonorrhoeae* NCCP11945<br>*N. meningitidis* serogroup A, strain Z2491<br>*N. meningitidis* serogroup C, strain 053442<br>*N. meningitidis* serogroup B, strain MC58	1463	0
6	*N. gonorrhoeae* NCCP11945<br>*N. meningitidis* serogroup C, strain 053442<br>*N. meningitidis* serogroup C, strain ATCC 700532<br>*N. meningitidis* serogroup B, strain MC58	1462	0
7	*N. gonorrhoeae* NCCP11945<br>*N. meningitidis* serogroup C, strain 053442<br>*N. gonorrhoeae* ATCC 700825 / FA 1090<br>*N. meningitidis* serogroup B, strain MC58	1411	17
8	*N. meningitidis* serogroup A, strain Z2491<br>*N. gonorrhoeae* ATCC 700825 / FA 1090<br>*N. meningitidis* serogroup C, strain ATCC 700532<br>*N. meningitidis* serogroup B, strain MC58	1404	3
9	*N. gonorrhoeae* ATCC 700825 / FA 1090<br>*N. meningitidis* serogroup C, strain 053442<br>*N. meningitidis* serogroup C, strain ATCC 700532<br>*N. meningitidis* serogroup B, strain MC58	1401	1
10	*N. gonorrhoeae* NCCP11945<br>*N. gonorrhoeae* ATCC 700825 / FA 1090<br>*N. meningitidis* serogroup C, strain 053442<br>*N. meningitidis* serogroup C, strain ATCC 700532	1424	9
11	*N. meningitidis* serogroup A, strain Z2491<br>*N. gonorrhoeae* ATCC 700825 / FA 1090<br>*N. meningitidis* serogroup C, strain 053442<br>*N. meningitidis* serogroup C, strain ATCC 700532	1427	3
12	*N. gonorrhoeae* NCCP11945<br>*N. meningitidis* serogroup A, strain Z2491<br>*N. meningitidis* serogroup C, strain 053442<br>*N. gonorrhoeae* ATCC 700825 / FA 1090	1416	1

13	*N. meningitidis* serogroup A, strain Z2491<br>*N. gonorrhoeae* NCCP11945<br>*N. gonorrhoeae* ATCC 700825 / FA 1090<br>*N. meningitidis* serogroup C, strain ATCC 700532	1420	4
14	*N. meningitidis* serogroup A, strain Z2491<br>*N. meningitidis* serogroup C, strain 053442<br>*N. gonorrhoeae* ATCC 700825 / FA 1090<br>*N. meningitidis* serogroup B, strain MC58	1403	0
