# Supplementary material for: Analysis and comparison of the pan-genomic properties of sixteen well-characterized bacterial genera
Source: BMC Microbiol. 2010 Oct 13;10:258. doi: 10.1186/1471-2180-10-258 (PMC3020658; doi:10.1186/1471-2180-10-258)
Supplement: Additional file 5 — Complete list of random groups. These tables list the random groups used for the analysis whose results are summarized in Tables 3 and 4 of the main paper. The column heading NC indicates the number of proteins in that group's core proteome, while NU indicates the number of proteins found in the proteomes of all members of that group, but no other isolates from the same genus. [file 1471-2180-10-258-S5.ZIP › Pseudomonas_2_isolates.pdf]

Random groups corresponding to *Pseudomonas* species with 2 isolates.

#	Members of random group	N_C	N_U
1	*P. aeruginosa* UCBPP-PA14<br>*P. fluorescens* PfO-1	3559	4
2	*P. mendocina* ymp<br>*P. entomophila* L48	2820	15
3	*P. aeruginosa* PA7<br>*P. fluorescens* PfO-1	3513	3
4	*P. putida* GB-1<br>*P. syringae* 1448A	3044	5
5	*P. mendocina* ymp<br>*P. fluorescens* Pf-5 / ATCC BAA-477	3015	15
6	*P. entomophila* L48<br>*P. syringae* 1448A	2994	1
7	*P. fluorescens* Pf-5 / ATCC BAA-477<br>*P. putida* W619	3582	9
8	*P. stutzeri* A1501<br>*P. aeruginosa* UCBPP-PA14	2689	0
9	*P. stutzeri* A1501<br>*P. aeruginosa* PA7	2733	14
10	*P. mendocina* ymp<br>*P. putida* W619	2863	8
11	*P. fluorescens* Pf-5 / ATCC BAA-477<br>*P. syringae* 1448A	3282	6
12	*P. mendocina* ymp<br>*P. aeruginosa* PA7	3192	2
13	*P. fluorescens* Pf-5 / ATCC BAA-477<br>*P. aeruginosa* UCBPP-PA14	3802	4
14	*P. entomophila* L48<br>*P. aeruginosa* UCBPP-PA14	3301	0
15	*P. putida* GB-1<br>*P. stutzeri* A1501	2492	5
16	*P. putida* F1 / ATCC 700007<br>*P. fluorescens* PfO-1	3670	5
17	*P. entomophila* L48<br>*P. putida* KT2440	3739	8
18	*P. aeruginosa* PA7<br>*P. entomophila* L48	3286	0
19	*P. putida* GB-1<br>*P. fluorescens* Pf-5 / ATCC BAA-477	3767	32
20	*P. putida* KT2440<br>*P. aeruginosa* UCBPP-PA14	3248	8
21	*P. mendocina* ymp<br>*P. aeruginosa* UCBPP-PA14	3203	1
22	*P. syringae* pathovar syringae, strain B728a<br>*P. putida* KT2440	3039	7
23	*P. syringae* pathovar syringae, strain B728a<br>*P. putida* W619	3145	5
24	*P. putida* F1 / ATCC 700007<br>*P. syringae* pathovar syringae, strain B728a	3087	6
25	*P. aeruginosa* LMG 12228 / ATCC 15692<br>*P. syringae* pathovar syringae, strain B728a	2929	0
