# Supplementary material for: Analysis and comparison of the pan-genomic properties of sixteen well-characterized bacterial genera
Source: BMC Microbiol. 2010 Oct 13;10:258. doi: 10.1186/1471-2180-10-258 (PMC3020658; doi:10.1186/1471-2180-10-258)
Supplement: Additional file 5 — Complete list of random groups. These tables list the random groups used for the analysis whose results are summarized in Tables 3 and 4 of the main paper. The column heading NC indicates the number of proteins in that group's core proteome, while NU indicates the number of proteins found in the proteomes of all members of that group, but no other isolates from the same genus. [file 1471-2180-10-258-S5.ZIP › Pseudomonas_3_isolates.pdf]

Random groups corresponding to *Pseudomonas* species with 3 isolates.

#	Members of random group	N_C	N_U
1	*P. fluorescens* Pf-5 / ATCC BAA-477<br>*P. aeruginosa* UCBPP-PA14<br>*P. fluorescens* PfO-1	3352	1
2	*P. fluorescens* Pf-5 / ATCC BAA-477<br>*P. putida* F1 / ATCC 700007<br>*P. putida* W619	3405	0
3	*P. aeruginosa* PA7<br>*P. putida* W619<br>*P. syringae* tomato, strain DC3000	2693	0
4	*P. syringae* 1448A<br>*P. syringae* tomato, strain DC3000<br>*P. fluorescens* PfO-1	3222	5
5	*P. mendocina* ymp<br>*P. stutzeri* A1501<br>*P. putida* F1 / ATCC 700007	2326	0
6	*P. mendocina* ymp<br>*P. entomophila* L48<br>*P. putida* W619	2715	0
7	*P. putida* GB-1<br>*P. syringae* pathovar syringae, strain B728a<br>*P. putida* W619	2940	0
8	*P. putida* F1 / ATCC 700007<br>*P. syringae* pathovar syringae, strain B728a<br>*P. syringae* 1448A	2933	0
9	*P. putida* GB-1<br>*P. putida* F1 / ATCC 700007<br>*P. syringae* 1448A	2914	0
10	*P. aeruginosa* LMG 12228 / ATCC 15692<br>*P. putida* W619<br>*P. fluorescens* PfO-1	3076	0
11	*P. aeruginosa* PA7<br>*P. aeruginosa* LMG 12228 / ATCC 15692<br>*P. putida* KT2440	3150	0
12	*P. mendocina* ymp<br>*P. putida* W619<br>*P. fluorescens* PfO-1	2745	0
13	*P. putida* GB-1<br>*P. entomophila* L48<br>*P. syringae* pathovar syringae, strain B728a	2875	1
14	*P. stutzeri* A1501<br>*P. aeruginosa* LMG 12228 / ATCC 15692<br>*P. aeruginosa* UCBPP-PA14	2653	1
15	*P. aeruginosa* PA7<br>*P. putida* KT2440<br>*P. syringae* pathovar syringae, strain B728a	2616	1
16	*P. putida* GB-1<br>*P. aeruginosa* PA7<br>*P. putida* F1 / ATCC 700007	3155	0

17	*P. mendocina* ymp<br>*P. putida* F1 / ATCC 700007<br>*P. putida* W619	2788	1
18	*P. stutzeri* A1501<br>*P. syringae* 1448A<br>*P. syringae* tomato, strain DC3000	2315	0
19	*P. mendocina* ymp<br>*P. fluorescens* Pf-5 / ATCC BAA-477<br>*P. syringae* 1448A	2494	0
20	*P. fluorescens* Pf-5 / ATCC BAA-477<br>*P. aeruginosa* LMG 12228 / ATCC 15692<br>*P. aeruginosa* UCBPP-PA14	3697	15
21	*P. aeruginosa* PA7<br>*P. putida* F1 / ATCC 700007<br>*P. putida* KT2440	3148	0
22	*P. putida* KT2440<br>*P. aeruginosa* UCBPP-PA14<br>*P. syringae* tomato, strain DC3000	2621	1
23	*P. fluorescens* Pf-5 / ATCC BAA-477<br>*P. putida* KT2440<br>*P. syringae* 1448A	2793	0
24	*P. stutzeri* A1501<br>*P. aeruginosa* LMG 12228 / ATCC 15692<br>*P. putida* KT2440	2303	0
25	*P. aeruginosa* LMG 12228 / ATCC 15692<br>*P. putida* KT2440<br>*P. fluorescens* PfO-1	3020	0
