# Supplementary material for: Analysis and comparison of the pan-genomic properties of sixteen well-characterized bacterial genera
Source: BMC Microbiol. 2010 Oct 13;10:258. doi: 10.1186/1471-2180-10-258 (PMC3020658; doi:10.1186/1471-2180-10-258)
Supplement: Additional file 5 — Complete list of random groups. These tables list the random groups used for the analysis whose results are summarized in Tables 3 and 4 of the main paper. The column heading NC indicates the number of proteins in that group's core proteome, while NU indicates the number of proteins found in the proteomes of all members of that group, but no other isolates from the same genus. [file 1471-2180-10-258-S5.ZIP › Pseudomonas_4_isolates.pdf]

Random groups corresponding to *Pseudomonas* species with 4 isolates.

#	Members of random group	N_C	N_U
1	*P. stutzeri* A1501<br>*P. putida* F1 / ATCC 700007<br>*P. syringae* pathovar syringae, strain B728a<br>*P. aeruginosa* UCBPP-PA14	2126	0
2	*P. putida* GB-1<br>*P. aeruginosa* LMG 12228 / ATCC 15692<br>*P. aeruginosa* UCBPP-PA14<br>*P. fluorescens* PfO-1	3054	0
3	*P. mendocina* ymp<br>*P. stutzeri* A1501<br>*P. aeruginosa* LMG 12228 / ATCC 15692<br>*P. fluorescens* PfO-1	2277	0
4	*P. stutzeri* A1501<br>*P. putida* KT2440<br>*P. putida* W619<br>*P. fluorescens* PfO-1	2337	0
5	*P. mendocina* ymp<br>*P. aeruginosa* LMG 12228 / ATCC 15692<br>*P. putida* F1 / ATCC 700007<br>*P. syringae* tomato, strain DC3000	2331	1
6	*P. mendocina* ymp<br>*P. stutzeri* A1501<br>*P. entomophila* L48<br>*P. aeruginosa* UCBPP-PA14	2201	0
7	*P. aeruginosa* LMG 12228 / ATCC 15692<br>*P. putida* W619<br>*P. putida* KT2440<br>*P. fluorescens* PfO-1	2949	1
8	*P. putida* GB-1<br>*P. fluorescens* Pf-5 / ATCC BAA-477<br>*P. stutzeri* A1501<br>*P. aeruginosa* UCBPP-PA14	2283	0
9	*P. putida* GB-1<br>*P. fluorescens* Pf-5 / ATCC BAA-477<br>*P. entomophila* L48<br>*P. putida* KT2440	3264	0
10	*P. aeruginosa* LMG 12228 / ATCC 15692<br>*P. putida* KT2440<br>*P. syringae* pathovar syringae, strain B728a<br>*P. aeruginosa* UCBPP-PA14	2594	0
11	*P. mendocina* ymp<br>*P. syringae* pathovar syringae, strain B728a<br>*P. syringae* 1448A<br>*P. fluorescens* PfO-1	2506	1
12	*P. putida* GB-1<br>*P. aeruginosa* UCBPP-PA14<br>*P. syringae* 1448A<br>*P. fluorescens* PfO-1	2590	0

13	*P. fluorescens* Pf-5 / ATCC BAA-477<br>*P. putida* F1 / ATCC 700007<br>*P. putida* W619<br>*P. syringae* pathovar syringae, strain B728a	2834	0
14	*P. putida* F1 / ATCC 700007<br>*P. putida* W619<br>*P. syringae* pathovar syringae, strain B728a<br>*P. aeruginosa* UCBPP-PA14	2622	0
15	*P. mendocina* ymp<br>*P. aeruginosa* PA7<br>*P. putida* F1 / ATCC 700007<br>*P. fluorescens* PfO-1	2601	0
16	*P. putida* GB-1<br>*P. aeruginosa* PA7<br>*P. putida* KT2440<br>*P. fluorescens* PfO-1	2973	0
17	*P. putida* GB-1<br>*P. fluorescens* Pf-5 / ATCC BAA-477<br>*P. syringae* pathovar syringae, strain B728a<br>*P. putida* W619	2826	0
18	*P. fluorescens* Pf-5 / ATCC BAA-477<br>*P. putida* W619<br>*P. aeruginosa* UCBPP-PA14<br>*P. syringae* tomato, strain DC3000	2629	0
19	*P. putida* GB-1<br>*P. entomophila* L48<br>*P. aeruginosa* UCBPP-PA14<br>*P. syringae* tomato, strain DC3000	2559	0
20	*P. entomophila* L48<br>*P. syringae* pathovar syringae, strain B728a<br>*P. aeruginosa* UCBPP-PA14<br>*P. syringae* tomato, strain DC3000	2590	0
21	*P. mendocina* ymp<br>*P. aeruginosa* PA7<br>*P. syringae* pathovar syringae, strain B728a<br>*P. syringae* 1448A	2424	0
22	*P. mendocina* ymp<br>*P. entomophila* L48<br>*P. syringae* pathovar syringae, strain B728a<br>*P. fluorescens* PfO-1	2435	0
23	*P. stutzeri* A1501<br>*P. fluorescens* Pf-5 / ATCC BAA-477<br>*P. aeruginosa* LMG 12228 / ATCC 15692<br>*P. putida* W619	2275	0
24	*P. aeruginosa* PA7<br>*P. putida* F1 / ATCC 700007<br>*P. putida* KT2440<br>*P. aeruginosa* UCBPP-PA14	3113	0

25	*P. mendocina* ymp<br>*P. putida* GB-1<br>*P. putida* F1 / ATCC 700007<br>*P. syringae* tomato, strain DC3000	2429	0
