# Supplementary material for: Analysis and comparison of the pan-genomic properties of sixteen well-characterized bacterial genera
Source: BMC Microbiol. 2010 Oct 13;10:258. doi: 10.1186/1471-2180-10-258 (PMC3020658; doi:10.1186/1471-2180-10-258)
Supplement: Additional file 5 — Complete list of random groups. These tables list the random groups used for the analysis whose results are summarized in Tables 3 and 4 of the main paper. The column heading NC indicates the number of proteins in that group's core proteome, while NU indicates the number of proteins found in the proteomes of all members of that group, but no other isolates from the same genus. [file 1471-2180-10-258-S5.ZIP › Rhizobium_2_isolates.pdf]

Random groups corresponding to *Rhizobium* species with 2 isolates.

#	Members of random group	N_C	N_U
1	*R. etli* CIAT 652<br>*R. leguminosarum* bv. viciae, strain 3841	4485	173
2	*R. etli* CFN 42 / ATCC 51251<br>*R. leguminosarum* bv. trifolii WSM2304	3482	25
3	*R. etli* CFN 42 / ATCC 51251<br>*R. leguminosarum* bv. viciae, strain 3841	4622	389
4	*R. etli* CIAT 652<br>*R. leguminosarum* bv. trifolii WSM2304	3661	115
