# Supplementary material for: Analysis and comparison of the pan-genomic properties of sixteen well-characterized bacterial genera
Source: BMC Microbiol. 2010 Oct 13;10:258. doi: 10.1186/1471-2180-10-258 (PMC3020658; doi:10.1186/1471-2180-10-258)
Supplement: Additional file 5 — Complete list of random groups. These tables list the random groups used for the analysis whose results are summarized in Tables 3 and 4 of the main paper. The column heading NC indicates the number of proteins in that group's core proteome, while NU indicates the number of proteins found in the proteomes of all members of that group, but no other isolates from the same genus. [file 1471-2180-10-258-S5.ZIP › Rickettsia_2_isolates.pdf]

Random groups corresponding to *Rickettsia* species with 2 isolates.

#	**Members of random group**	$\mathbf{N_C}$	$\mathbf{N_U}$
1	*R. prowazekii* Madrid E<br>*R. rickettsii* Sheila Smith	773	0
2	*R. conorii* ATCC VR-613 / Malish 7<br>*R. rickettsii* Sheila Smith	1099	11
3	*R. akari* Hartford<br>*R. typhi* Wilmington / ATCC VR-144	779	0
4	*R. bellii* OSU 85-389<br>*R. felis* ATCC VR-1525 / URRWXCal2	992	1
5	*R. conorii* ATCC VR-613 / Malish 7<br>*R. typhi* Wilmington / ATCC VR-144	782	0
6	*R. rickettsii* Iowa<br>*R. akari* Hartford	866	3
7	*R. prowazekii* Madrid E<br>*R. bellii* RML369-C	782	0
8	*R. conorii* ATCC VR-613 / Malish 7<br>*R. prowazekii* Madrid E	785	0
9	*R. canadensis* McKiel<br>*R. felis* ATCC VR-1525 / URRWXCal2	824	4
10	*R. rickettsii* Iowa<br>*R. conorii* ATCC VR-613 / Malish 7	1101	12
11	*R. rickettsii* Sheila Smith<br>*R. felis* ATCC VR-1525 / URRWXCal2	952	1
12	*R. conorii* ATCC VR-613 / Malish 7<br>*R. bellii* RML369-C	938	0
13	*R. rickettsii* Sheila Smith<br>*R. typhi* Wilmington / ATCC VR-144	771	0
14	*R. akari* Hartford<br>*R. prowazekii* Madrid E	775	0
15	*R. rickettsii* Iowa<br>*R. felis* ATCC VR-1525 / URRWXCal2	951	1
16	*R. bellii* OSU 85-389<br>*R. rickettsii* Sheila Smith	853	0
17	*R. typhi* Wilmington / ATCC VR-144<br>*R. felis* ATCC VR-1525 / URRWXCal2	799	2
18	*R. massiliae* Mtu5<br>*R. prowazekii* Madrid E	764	0
19	*R. rickettsii* Iowa<br>*R. prowazekii* Madrid E	776	0
20	*R. akari* Hartford<br>*R. canadensis* McKiel	775	0
21	*R. canadensis* McKiel<br>*R. bellii* RML369-C	798	1
22	*R. canadensis* McKiel<br>*R. prowazekii* Madrid E	743	0
23	*R. massiliae* Mtu5<br>*R. akari* Hartford	828	0
24	*R. massiliae* Mtu5<br>*R. typhi* Wilmington / ATCC VR-144	766	1
25	*R. conorii* ATCC VR-613 / Malish 7<br>*R. felis* ATCC VR-1525 / URRWXCal2	997	6
