# Supplementary material for: Analysis and comparison of the pan-genomic properties of sixteen well-characterized bacterial genera
Source: BMC Microbiol. 2010 Oct 13;10:258. doi: 10.1186/1471-2180-10-258 (PMC3020658; doi:10.1186/1471-2180-10-258)
Supplement: Additional file 5 — Complete list of random groups. These tables list the random groups used for the analysis whose results are summarized in Tables 3 and 4 of the main paper. The column heading NC indicates the number of proteins in that group's core proteome, while NU indicates the number of proteins found in the proteomes of all members of that group, but no other isolates from the same genus. [file 1471-2180-10-258-S5.ZIP › Shigella_2_isolates.pdf]

Random groups corresponding to *Shigella* species with 2 isolates.

#	Members of random group	N_C	N_U
1	*S. sonnei* Ss046<br>*S. boydii* serovar 18, strain CDC 3083-94	3109	26
2	*S. flexneri* serovar 2a, strain 301<br>*S. boydii* serovar 18, strain CDC 3083-94	3017	8
3	*S. flexneri* serovar 2a, strain 301<br>*S. dysenteriae* serovar 1, strain Sd97 / Sd197	2907	6
4	*S. sonnei* Ss046<br>*S. flexneri* serovar 5b, strain 8401	3142	15
5	*S. flexneri* serovar 2a, strain ATCC 700930<br>*S. dysenteriae* serovar 1, strain Sd97 / Sd197	2781	4
6	*S. boydii* serovar 4, strain Sb227<br>*S. dysenteriae* serovar 1, strain Sd97 / Sd197	2837	12
7	*S. sonnei* Ss046<br>*S. flexneri* serovar 2a, strain ATCC 700930	3083	2
8	*S. boydii* serovar 18, strain CDC 3083-94<br>*S. flexneri* serovar 5b, strain 8401	2948	8
9	*S. boydii* serovar 18, strain CDC 3083-94<br>*S. flexneri* serovar 2a, strain ATCC 700930	2885	3
10	*S. dysenteriae* serovar 1, strain Sd97 / Sd197<br>*S. flexneri* serovar 5b, strain 8401	2799	13
11	*S. sonnei* Ss046<br>*S. flexneri* serovar 2a, strain 301	3217	9
12	*S. boydii* serovar 4, strain Sb227<br>*S. flexneri* serovar 2a, strain ATCC 700930	2942	1
13	*S. sonnei* Ss046<br>*S. boydii* serovar 4, strain Sb227	3156	30
14	*S. flexneri* serovar 2a, strain 301<br>*S. boydii* serovar 4, strain Sb227	3033	4
15	*S. boydii* serovar 18, strain CDC 3083-94<br>*S. dysenteriae* serovar 1, strain Sd97 / Sd197	2830	18
16	*S. sonnei* Ss046<br>*S. dysenteriae* serovar 1, strain Sd97 / Sd197	2959	44
17	*S. boydii* serovar 4, strain Sb227<br>*S. flexneri* serovar 5b, strain 8401	3006	10
