# Supplementary material for: Analysis and comparison of the pan-genomic properties of sixteen well-characterized bacterial genera
Source: BMC Microbiol. 2010 Oct 13;10:258. doi: 10.1186/1471-2180-10-258 (PMC3020658; doi:10.1186/1471-2180-10-258)
Supplement: Additional file 5 — Complete list of random groups. These tables list the random groups used for the analysis whose results are summarized in Tables 3 and 4 of the main paper. The column heading NC indicates the number of proteins in that group's core proteome, while NU indicates the number of proteins found in the proteomes of all members of that group, but no other isolates from the same genus. [file 1471-2180-10-258-S5.ZIP › Shigella_3_isolates.pdf]

Random groups corresponding to *Shigella* species with 3 isolates.

#	Members of random group	N_C	N_U
1	*S. sonnei* Ss046<br>*S. flexneri* serovar 2a, strain ATCC 700930<br>*S. dysenteriae* serovar 1, strain Sd97 / Sd197	2630	0
2	*S. flexneri* serovar 2a, strain 301<br>*S. boydii* serovar 4, strain Sb227<br>*S. flexneri* serovar 5b, strain 8401	2868	3
3	*S. flexneri* serovar 2a, strain ATCC 700930<br>*S. boydii* serovar 18, strain CDC 3083-94<br>*S. dysenteriae* serovar 1, strain Sd97 / Sd197	2537	0
4	*S. flexneri* serovar 2a, strain 301<br>*S. boydii* serovar 18, strain CDC 3083-94<br>*S. flexneri* serovar 5b, strain 8401	2824	0
5	*S. sonnei* Ss046<br>*S. boydii* serovar 18, strain CDC 3083-94<br>*S. flexneri* serovar 5b, strain 8401	2796	3
6	*S. boydii* serovar 4, strain Sb227<br>*S. boydii* serovar 18, strain CDC 3083-94<br>*S. flexneri* serovar 5b, strain 8401	2789	15
7	*S. sonnei* Ss046<br>*S. boydii* serovar 4, strain Sb227<br>*S. dysenteriae* serovar 1, strain Sd97 / Sd197	2705	23
8	*S. sonnei* Ss046<br>*S. flexneri* serovar 2a, strain 301<br>*S. flexneri* serovar 2a, strain ATCC 700930	3022	10
9	*S. boydii* serovar 4, strain Sb227<br>*S. boydii* serovar 18, strain CDC 3083-94<br>*S. dysenteriae* serovar 1, strain Sd97 / Sd197	2665	20
10	*S. sonnei* Ss046<br>*S. boydii* serovar 4, strain Sb227<br>*S. flexneri* serovar 5b, strain 8401	2840	6
11	*S. sonnei* Ss046<br>*S. flexneri* serovar 2a, strain ATCC 700930<br>*S. boydii* serovar 18, strain CDC 3083-94	2744	0
12	*S. boydii* serovar 18, strain CDC 3083-94<br>*S. dysenteriae* serovar 1, strain Sd97 / Sd197<br>*S. flexneri* serovar 5b, strain 8401	2564	0
13	*S. sonnei* Ss046<br>*S. boydii* serovar 4, strain Sb227<br>*S. flexneri* serovar 2a, strain ATCC 700930	2799	3
14	*S. boydii* serovar 4, strain Sb227<br>*S. flexneri* serovar 2a, strain 301<br>*S. dysenteriae* serovar 1, strain Sd97 / Sd197	2637	4
15	*S. sonnei* Ss046<br>*S. flexneri* serovar 2a, strain 301<br>*S. flexneri* serovar 5b, strain 8401	3004	7
16	*S. sonnei* Ss046<br>*S. boydii* serovar 4, strain Sb227<br>*S. boydii* serovar 18, strain CDC 3083-94	2901	50

17	*S. flexneri* serovar 2a, strain 301<br>*S. dysenteriae* serovar 1, strain Sd97 / Sd197<br>*S. flexneri* serovar 5b, strain 8401	2694	3
18	*S. boydii* serovar 4, strain Sb227<br>*S. flexneri* serovar 2a, strain ATCC 700930<br>*S. dysenteriae* serovar 1, strain Sd97 / Sd197	2570	0
19	*S. flexneri* serovar 2a, strain 301<br>*S. boydii* serovar 18, strain CDC 3083-94<br>*S. dysenteriae* serovar 1, strain Sd97 / Sd197	2639	6
20	*S. flexneri* serovar 2a, strain 301<br>*S. boydii* serovar 18, strain CDC 3083-94<br>*S. flexneri* serovar 2a, strain ATCC 700930	2838	6
21	*S. boydii* serovar 4, strain Sb227<br>*S. boydii* serovar 18, strain CDC 3083-94<br>*S. flexneri* serovar 2a, strain ATCC 700930	2733	0
22	*S. sonnei* Ss046<br>*S. flexneri* serovar 2a, strain ATCC 700930<br>*S. flexneri* serovar 5b, strain 8401	2971	1
23	*S. flexneri* serovar 2a, strain 301<br>*S. boydii* serovar 4, strain Sb227<br>*S. boydii* serovar 18, strain CDC 3083-94	2814	5
24	*S. flexneri* serovar 2a, strain ATCC 700930<br>*S. boydii* serovar 18, strain CDC 3083-94<br>*S. flexneri* serovar 5b, strain 8401	2797	2
25	*S. flexneri* serovar 2a, strain 301<br>*S. boydii* serovar 4, strain Sb227<br>*S. flexneri* serovar 2a, strain ATCC 700930	2890	1
