# Supplementary material for: Analysis and comparison of the pan-genomic properties of sixteen well-characterized bacterial genera
Source: BMC Microbiol. 2010 Oct 13;10:258. doi: 10.1186/1471-2180-10-258 (PMC3020658; doi:10.1186/1471-2180-10-258)
Supplement: Additional file 5 — Complete list of random groups. These tables list the random groups used for the analysis whose results are summarized in Tables 3 and 4 of the main paper. The column heading NC indicates the number of proteins in that group's core proteome, while NU indicates the number of proteins found in the proteomes of all members of that group, but no other isolates from the same genus. [file 1471-2180-10-258-S5.ZIP › Staphylococcus_14_isolates.pdf]

Random groups corresponding to *Staphylococcus* species with 14 isolates.

#	Members of random group	N_C	N_U
1	*S. aureus* Mu3 / ATCC 700698<br>*S. aureus* USA300 / TCH1516<br>*S. aureus* Mu50 / ATCC 700699<br>*S. aureus* NCTC 8325<br>*S. aureus* JH9<br>*S. aureus* USA300<br>*S. saprophyticus* ATCC 15305<br>*S. epidermidis* ATCC 12228<br>*S. epidermidis* ATCC 35984 / RP62A<br>*S. aureus* N315<br>*S. aureus* JH1<br>*S. aureus* Newman<br>*S. aureus* bovine RF122 / ET3-1 / RF122<br>*S. haemolyticus* JCSC1435	1427	0
2	*S. aureus* Mu3 / ATCC 700698<br>*S. aureus* USA300 / TCH1516<br>*S. aureus* Mu50 / ATCC 700699<br>*S. aureus* NCTC 8325<br>*S. aureus* JH9<br>*S. aureus* USA300<br>*S. saprophyticus* ATCC 15305<br>*S. epidermidis* ATCC 12228<br>*S. aureus* N315<br>*S. aureus* JH1<br>*S. aureus* COL<br>*S. aureus* Newman<br>*S. haemolyticus* JCSC1435<br>*S. aureus* bovine RF122 / ET3-1 / RF122	1427	0
3	*S. aureus* Mu3 / ATCC 700698<br>*S. aureus* USA300 / TCH1516<br>*S. aureus* MRSA252<br>*S. aureus* NCTC 8325<br>*S. aureus* JH9<br>*S. saprophyticus* ATCC 15305<br>*S. epidermidis* ATCC 35984 / RP62A<br>*S. epidermidis* ATCC 12228<br>*S. aureus* MSSA476<br>*S. aureus* JH1<br>*S. aureus* COL<br>*S. aureus* Newman<br>*S. haemolyticus* JCSC1435<br>*S. aureus* MW2	1438	0

4	*S. aureus* Mu3 / ATCC 700698<br>*S. aureus* USA300 / TCH1516<br>*S. aureus* MRSA252<br>*S. aureus* Mu50 / ATCC 700699<br>*S. aureus* NCTC 8325<br>*S. aureus* USA300<br>*S. saprophyticus* ATCC 15305<br>*S. epidermidis* ATCC 35984 / RP62A<br>*S. aureus* JH1<br>*S. aureus* MSSA476<br>*S. aureus* COL<br>*S. aureus* bovine RF122 / ET3-1 / RF122<br>*S. aureus* Newman<br>*S. aureus* MW2	1447	0
5	*S. aureus* Mu3 / ATCC 700698<br>*S. aureus* USA300 / TCH1516<br>*S. aureus* MRSA252<br>*S. aureus* Mu50 / ATCC 700699<br>*S. aureus* NCTC 8325<br>*S. aureus* JH9<br>*S. aureus* USA300<br>*S. epidermidis* ATCC 35984 / RP62A<br>*S. aureus* N315<br>*S. aureus* MSSA476<br>*S. aureus* JH1<br>*S. aureus* COL<br>*S. haemolyticus* JCSC1435<br>*S. aureus* MW2	1535	0
6	*S. aureus* Mu3 / ATCC 700698<br>*S. aureus* USA300 / TCH1516<br>*S. aureus* NCTC 8325<br>*S. aureus* JH9<br>*S. saprophyticus* ATCC 15305<br>*S. aureus* USA300<br>*S. epidermidis* ATCC 12228<br>*S. epidermidis* ATCC 35984 / RP62A<br>*S. aureus* N315<br>*S. aureus* JH1<br>*S. aureus* Newman<br>*S. aureus* bovine RF122 / ET3-1 / RF122<br>*S. haemolyticus* JCSC1435<br>*S. aureus* MW2	1428	0

7	*S. aureus* USA300 / TCH1516<br>*S. aureus* Mu50 / ATCC 700699<br>*S. aureus* NCTC 8325<br>*S. aureus* USA300<br>*S. aureus* JH9<br>*S. epidermidis* ATCC 35984 / RP62A<br>*S. epidermidis* ATCC 12228<br>*S. aureus* N315<br>*S. aureus* MSSA476<br>*S. aureus* JH1<br>*S. aureus* bovine RF122 / ET3-1 / RF122<br>*S. aureus* Newman<br>*S. haemolyticus* JCSC1435<br>*S. aureus* MW2	1501	0
8	*S. aureus* Mu3 / ATCC 700698<br>*S. aureus* USA300 / TCH1516<br>*S. aureus* MRSA252<br>*S. aureus* Mu50 / ATCC 700699<br>*S. aureus* NCTC 8325<br>*S. aureus* JH9<br>*S. aureus* USA300<br>*S. epidermidis* ATCC 35984 / RP62A<br>*S. aureus* JH1<br>*S. aureus* COL<br>*S. aureus* bovine RF122 / ET3-1 / RF122<br>*S. aureus* Newman<br>*S. haemolyticus* JCSC1435<br>*S. aureus* MW2	1492	0
9	*S. aureus* Mu3 / ATCC 700698<br>*S. aureus* MRSA252<br>*S. aureus* Mu50 / ATCC 700699<br>*S. saprophyticus* ATCC 15305<br>*S. aureus* JH9<br>*S. aureus* USA300<br>*S. epidermidis* ATCC 12228<br>*S. epidermidis* ATCC 35984 / RP62A<br>*S. aureus* N315<br>*S. aureus* MSSA476<br>*S. aureus* JH1<br>*S. aureus* COL<br>*S. aureus* bovine RF122 / ET3-1 / RF122<br>*S. aureus* Newman	1523	0

10	*S. aureus* Mu3 / ATCC 700698<br>*S. aureus* MRSA252<br>*S. aureus* Mu50 / ATCC 700699<br>*S. aureus* NCTC 8325<br>*S. aureus* USA300<br>*S. epidermidis* ATCC 35984 / RP62A<br>*S. epidermidis* ATCC 12228<br>*S. aureus* JH1<br>*S. aureus* MSSA476<br>*S. aureus* COL<br>*S. haemolyticus* JCSC1435<br>*S. aureus* Newman<br>*S. aureus* bovine RF122 / ET3-1 / RF122<br>*S. aureus* MW2	1562	0
11	*S. aureus* Mu3 / ATCC 700698<br>*S. aureus* USA300 / TCH1516<br>*S. aureus* MRSA252<br>*S. aureus* NCTC 8325<br>*S. aureus* JH9<br>*S. aureus* USA300<br>*S. epidermidis* ATCC 35984 / RP62A<br>*S. aureus* N315<br>*S. aureus* JH1<br>*S. aureus* MSSA476<br>*S. haemolyticus* JCSC1435<br>*S. aureus* Newman<br>*S. aureus* bovine RF122 / ET3-1 / RF122<br>*S. aureus* MW2	1500	0
12	*S. aureus* Mu3 / ATCC 700698<br>*S. aureus* USA300 / TCH1516<br>*S. aureus* MRSA252<br>*S. aureus* Mu50 / ATCC 700699<br>*S. aureus* NCTC 8325<br>*S. saprophyticus* ATCC 15305<br>*S. aureus* USA300<br>*S. epidermidis* ATCC 12228<br>*S. epidermidis* ATCC 35984 / RP62A<br>*S. aureus* N315<br>*S. aureus* JH1<br>*S. aureus* MSSA476<br>*S. aureus* COL<br>*S. aureus* Newman	1466	0

13	*S. aureus* Mu3 / ATCC 700698<br>*S. aureus* USA300 / TCH1516<br>*S. aureus* MRSA252<br>*S. aureus* NCTC 8325<br>*S. aureus* JH9<br>*S. epidermidis* ATCC 35984 / RP62A<br>*S. aureus* N315<br>*S. aureus* JH1<br>*S. aureus* MSSA476<br>*S. aureus* COL<br>*S. aureus* bovine RF122 / ET3-1 / RF122<br>*S. aureus* Newman<br>*S. haemolyticus* JCSC1435<br>*S. aureus* MW2	1498	0
14	*S. aureus* Mu3 / ATCC 700698<br>*S. aureus* USA300 / TCH1516<br>*S. aureus* MRSA252<br>*S. aureus* Mu50 / ATCC 700699<br>*S. saprophyticus* ATCC 15305<br>*S. aureus* JH9<br>*S. aureus* USA300<br>*S. epidermidis* ATCC 12228<br>*S. epidermidis* ATCC 35984 / RP62A<br>*S. aureus* N315<br>*S. aureus* MSSA476<br>*S. aureus* JH1<br>*S. aureus* bovine RF122 / ET3-1 / RF122<br>*S. aureus* MW2	1468	0
15	*S. aureus* Mu3 / ATCC 700698<br>*S. aureus* MRSA252<br>*S. aureus* NCTC 8325<br>*S. aureus* JH9<br>*S. saprophyticus* ATCC 15305<br>*S. aureus* USA300<br>*S. epidermidis* ATCC 35984 / RP62A<br>*S. aureus* N315<br>*S. aureus* MSSA476<br>*S. aureus* COL<br>*S. aureus* bovine RF122 / ET3-1 / RF122<br>*S. aureus* Newman<br>*S. haemolyticus* JCSC1435<br>*S. aureus* MW2	1492	0

16	*S. aureus* Mu3 / ATCC 700698<br>*S. aureus* USA300 / TCH1516<br>*S. aureus* Mu50 / ATCC 700699<br>*S. aureus* JH9<br>*S. saprophyticus* ATCC 15305<br>*S. aureus* USA300<br>*S. epidermidis* ATCC 12228<br>*S. epidermidis* ATCC 35984 / RP62A<br>*S. aureus* N315<br>*S. aureus* JH1<br>*S. aureus* COL<br>*S. aureus* bovine RF122 / ET3-1 / RF122<br>*S. haemolyticus* JCSC1435<br>*S. aureus* MW2	1433	0
17	*S. aureus* Mu3 / ATCC 700698<br>*S. aureus* MRSA252<br>*S. aureus* Mu50 / ATCC 700699<br>*S. aureus* NCTC 8325<br>*S. aureus* USA300<br>*S. aureus* JH9<br>*S. epidermidis* ATCC 12228<br>*S. epidermidis* ATCC 35984 / RP62A<br>*S. aureus* N315<br>*S. aureus* JH1<br>*S. aureus* bovine RF122 / ET3-1 / RF122<br>*S. aureus* Newman<br>*S. haemolyticus* JCSC1435<br>*S. aureus* MW2	1574	0
18	*S. aureus* Mu3 / ATCC 700698<br>*S. aureus* USA300 / TCH1516<br>*S. aureus* MRSA252<br>*S. aureus* Mu50 / ATCC 700699<br>*S. aureus* NCTC 8325<br>*S. aureus* JH9<br>*S. epidermidis* ATCC 35984 / RP62A<br>*S. epidermidis* ATCC 12228<br>*S. aureus* N315<br>*S. aureus* JH1<br>*S. aureus* bovine RF122 / ET3-1 / RF122<br>*S. haemolyticus* JCSC1435<br>*S. aureus* Newman<br>*S. aureus* MW2	1502	0

19	*S. aureus* Mu3 / ATCC 700698<br>*S. aureus* MRSA252<br>*S. aureus* NCTC 8325<br>*S. aureus* JH9<br>*S. saprophyticus* ATCC 15305<br>*S. epidermidis* ATCC 35984 / RP62A<br>*S. epidermidis* ATCC 12228<br>*S. aureus* N315<br>*S. aureus* MSSA476<br>*S. aureus* JH1<br>*S. aureus* COL<br>*S. aureus* bovine RF122 / ET3-1 / RF122<br>*S. aureus* Newman<br>*S. aureus* MW2	1528	0
20	*S. aureus* Mu3 / ATCC 700698<br>*S. aureus* USA300 / TCH1516<br>*S. aureus* MRSA252<br>*S. aureus* Mu50 / ATCC 700699<br>*S. aureus* NCTC 8325<br>*S. saprophyticus* ATCC 15305<br>*S. aureus* JH9<br>*S. aureus* USA300<br>*S. aureus* N315<br>*S. aureus* MSSA476<br>*S. aureus* JH1<br>*S. aureus* COL<br>*S. aureus* bovine RF122 / ET3-1 / RF122<br>*S. aureus* MW2	1573	1
21	*S. aureus* Mu3 / ATCC 700698<br>*S. aureus* USA300 / TCH1516<br>*S. aureus* MRSA252<br>*S. aureus* Mu50 / ATCC 700699<br>*S. aureus* NCTC 8325<br>*S. aureus* USA300<br>*S. aureus* JH9<br>*S. saprophyticus* ATCC 15305<br>*S. epidermidis* ATCC 12228<br>*S. epidermidis* ATCC 35984 / RP62A<br>*S. aureus* N315<br>*S. aureus* MSSA476<br>*S. aureus* COL<br>*S. haemolyticus* JCSC1435	1444	0

22	*S. aureus* Mu3 / ATCC 700698<br>*S. aureus* Mu50 / ATCC 700699<br>*S. aureus* USA300<br>*S. aureus* JH9<br>*S. saprophyticus* ATCC 15305<br>*S. epidermidis* ATCC 12228<br>*S. aureus* N315<br>*S. aureus* MSSA476<br>*S. aureus* JH1<br>*S. aureus* COL<br>*S. aureus* bovine RF122 / ET3-1 / RF122<br>*S. haemolyticus* JCSC1435<br>*S. aureus* Newman<br>*S. aureus* MW2	1507	0
23	*S. aureus* Mu3 / ATCC 700698<br>*S. aureus* USA300 / TCH1516<br>*S. aureus* MRSA252<br>*S. aureus* Mu50 / ATCC 700699<br>*S. aureus* NCTC 8325<br>*S. aureus* USA300<br>*S. saprophyticus* ATCC 15305<br>*S. epidermidis* ATCC 35984 / RP62A<br>*S. aureus* N315<br>*S. aureus* JH1<br>*S. aureus* MSSA476<br>*S. aureus* COL<br>*S. aureus* bovine RF122 / ET3-1 / RF122<br>*S. aureus* MW2	1463	0
24	*S. aureus* USA300 / TCH1516<br>*S. aureus* MRSA252<br>*S. aureus* Mu50 / ATCC 700699<br>*S. aureus* NCTC 8325<br>*S. aureus* USA300<br>*S. saprophyticus* ATCC 15305<br>*S. epidermidis* ATCC 12228<br>*S. epidermidis* ATCC 35984 / RP62A<br>*S. aureus* N315<br>*S. aureus* JH1<br>*S. aureus* COL<br>*S. haemolyticus* JCSC1435<br>*S. aureus* Newman<br>*S. aureus* MW2	1432	0

25	*S. aureus* Mu3 / ATCC 700698<br>*S. aureus* USA300 / TCH1516<br>*S. aureus* MRSA252<br>*S. aureus* Mu50 / ATCC 700699<br>*S. aureus* NCTC 8325<br>*S. aureus* USA300<br>*S. epidermidis* ATCC 35984 / RP62A<br>*S. epidermidis* ATCC 12228<br>*S. aureus* N315<br>*S. aureus* JH1<br>*S. aureus* COL<br>*S. haemolyticus* JCSC1435<br>*S. aureus* bovine RF122 / ET3-1 / RF122<br>*S. aureus* MW2	1500	0
