# Supplementary material for: Analysis and comparison of the pan-genomic properties of sixteen well-characterized bacterial genera
Source: BMC Microbiol. 2010 Oct 13;10:258. doi: 10.1186/1471-2180-10-258 (PMC3020658; doi:10.1186/1471-2180-10-258)
Supplement: Additional file 5 — Complete list of random groups. These tables list the random groups used for the analysis whose results are summarized in Tables 3 and 4 of the main paper. The column heading NC indicates the number of proteins in that group's core proteome, while NU indicates the number of proteins found in the proteomes of all members of that group, but no other isolates from the same genus. [file 1471-2180-10-258-S5.ZIP › Staphylococcus_2_isolates.pdf]

Random groups corresponding to *Staphylococcus* species with 2 isolates.

#	Members of random group	N_C	N_U
1	*S. aureus* Mu3 / ATCC 700698<br>*S. haemolyticus* JCSC1435	1864	0
2	*S. haemolyticus* JCSC1435<br>*S. epidermidis* ATCC 35984 / RP62A	1812	5
3	*S. epidermidis* ATCC 12228<br>*S. aureus* MW2	1817	0
4	*S. aureus* USA300<br>*S. epidermidis* ATCC 12228	1815	1
5	*S. saprophyticus* ATCC 15305<br>*S. epidermidis* ATCC 35984 / RP62A	1713	2
6	*S. aureus* MSSA476<br>*S. epidermidis* ATCC 12228	1805	1
7	*S. aureus* Newman<br>*S. epidermidis* ATCC 35984 / RP62A	1769	0
8	*S. aureus* bovine RF122 / ET3-1 / RF122<br>*S. saprophyticus* ATCC 15305	1721	3
9	*S. aureus* Mu50 / ATCC 700699<br>*S. epidermidis* ATCC 12228	1821	1
10	*S. aureus* JH9<br>*S. epidermidis* ATCC 12228	1820	0
11	*S. aureus* MW2<br>*S. epidermidis* ATCC 35984 / RP62A	1814	0
12	*S. haemolyticus* JCSC1435<br>*S. aureus* USA300	1851	1
13	*S. aureus* bovine RF122 / ET3-1 / RF122<br>*S. epidermidis* ATCC 12228	1755	0
14	*S. aureus* MSSA476<br>*S. haemolyticus* JCSC1435	1833	2
15	*S. aureus* NCTC 8325<br>*S. epidermidis* ATCC 35984 / RP62A	1789	0
16	*S. aureus* MRSA252<br>*S. epidermidis* ATCC 12228	1804	1
17	*S. aureus* Newman<br>*S. haemolyticus* JCSC1435	1832	1
18	*S. aureus* JH9<br>*S. epidermidis* ATCC 35984 / RP62A	1830	0
19	*S. aureus* N315<br>*S. epidermidis* ATCC 35984 / RP62A	1829	0
20	*S. aureus* Newman<br>*S. saprophyticus* ATCC 15305	1734	0
21	*S. aureus* COL<br>*S. saprophyticus* ATCC 15305	1743	0
22	*S. haemolyticus* JCSC1435<br>*S. aureus* MW2	1850	1
23	*S. aureus* N315<br>*S. epidermidis* ATCC 12228	1819	0
24	*S. saprophyticus* ATCC 15305<br>*S. epidermidis* ATCC 12228	1721	3
25	*S. aureus* COL<br>*S. epidermidis* ATCC 12228	1792	1
