# Supplementary material for: Analysis and comparison of the pan-genomic properties of sixteen well-characterized bacterial genera
Source: BMC Microbiol. 2010 Oct 13;10:258. doi: 10.1186/1471-2180-10-258 (PMC3020658; doi:10.1186/1471-2180-10-258)
Supplement: Additional file 5 — Complete list of random groups. These tables list the random groups used for the analysis whose results are summarized in Tables 3 and 4 of the main paper. The column heading NC indicates the number of proteins in that group's core proteome, while NU indicates the number of proteins found in the proteomes of all members of that group, but no other isolates from the same genus. [file 1471-2180-10-258-S5.ZIP › Streptococcus_13_isolates.pdf]

Random groups corresponding to *Streptococcus* species with 13 isolates.

#	Members of random group	N_C	N_U
	S. sanguinis SK36		
	S. thermophilus ATCC BAA-250 / LMG 18311		
	S. equi MGCS10565		
	S. pneumoniae serovar 2, strain NCTC 7466		
	S. suis 05ZYH33		
	S. pyogenes serovar M12, strain MGAS2096		
1	*S. pyogenes* serovar M1, strain ATCC BAA-947	802	0
	S. pyogenes serovar M2, strain MGAS10270		
	S. pyogenes serovar M28, strain MGAS6180		
	S. pyogenes serovar M3, strain SSI-1		
	S. pneumoniae ATCC BAA-255 / R6		
	S. pyogenes NZ131		
	S. pyogenes serovar M18, strain MGAS8232		
	S. thermophilus ATCC BAA-250 / LMG 18311		
	S. pyogenes serovar M3, strain ATCC BAA-595		
	S. pneumoniae serovar 2, strain NCTC 7466		
	S. agalactiae serovar V, strain ATCC BAA-611		
	S. pyogenes serovar M12, strain MGAS2096		
	S. pyogenes serovar M1, strain ATCC BAA-947		
2	*S. pyogenes* serovar M28, strain MGAS6180	838	0
	S. pyogenes serovar M3, strain SSI-1		
	S. agalactiae serovar Ia, strain ATCC 27591		
	S. agalactiae serovar III, strain NEM316		
	S. pyogenes NZ131		
	S. pyogenes serovar M5, strain Manfredo		
	S. pyogenes serovar M6, strain ATCC BAA-946)		
	S. sanguinis SK36		
	S. pneumoniae serovar 19F, strain G54		
	S. pyogenes serovar M3, strain ATCC BAA-595		
	S. thermophilus ATCC BAA-250 / LMG 18311		
	S. pneumoniae serovar 2, strain NCTC 7466		
	S. suis 05ZYH33		
3	*S. pyogenes* serovar M12, strain MGAS2096	794	0
	S. pyogenes serovar M28, strain MGAS6180		
	S. agalactiae serovar Ia, strain ATCC 27591		
	S. pyogenes serovar M1, strain ATCC 700294		
	S. pyogenes NZ131		
	S. pyogenes serovar M5, strain Manfredo		
	S. thermophilus ATCC BAA-491 / LMD-9		

4	*S. sanguinis* SK36<br>*S. suis* 98HAH33<br>*S. equi* MGCS10565<br>*S. pneumoniae* CGSP14<br>*S. agalactiae* serovar V, strain ATCC BAA-611<br>*S. pyogenes* serovar M2, strain MGAS10270<br>*S. pyogenes* serovar M28, strain MGAS6180<br>*S. pyogenes* serovar M3, strain SSI-1<br>*S. agalactiae* serovar Ia, strain ATCC 27591<br>*S. pyogenes* serovar M12, strain MGAS9429<br>*S. pneumoniae* Hungary19A-6<br>*S. pyogenes* serovar M18, strain MGAS8232<br>*S. thermophilus* ATCC BAA-491 / LMD-9	774	0
5	*S. sanguinis* SK36<br>*S. pyogenes* serovar M3, strain ATCC BAA-595<br>*S. pneumoniae* CGSP14<br>*S. pyogenes* serovar M1, strain ATCC BAA-947<br>*S. pyogenes* serovar M12, strain MGAS2096<br>*S. pyogenes* serovar M2, strain MGAS10270<br>*S. pyogenes* serovar M28, strain MGAS6180<br>*S. pyogenes* serovar M3, strain SSI-1<br>*S. pyogenes* serovar M1, strain ATCC 700294<br>*S. thermophilus* CNRZ 1066<br>*S. pyogenes* NZ131<br>*S. pyogenes* serovar M6, strain ATCC BAA-946)<br>*S. thermophilus* ATCC BAA-491 / LMD-9	826	0
6	*S. suis* 98HAH33<br>*S. pyogenes* serovar M3, strain ATCC BAA-595<br>*S. thermophilus* ATCC BAA-250 / LMG 18311<br>*S. equi* MGCS10565<br>*S. pyogenes* serovar M12, strain MGAS2096<br>*S. pyogenes* serovar M4, strain MGAS10750<br>*S. pyogenes* serovar M3, strain SSI-1<br>*S. agalactiae* serovar Ia, strain ATCC 27591<br>*S. agalactiae* serovar III, strain NEM316<br>*S. thermophilus* CNRZ 1066<br>*S. pyogenes* NZ131<br>*S. pyogenes* serovar M12, strain MGAS9429<br>*S. thermophilus* ATCC BAA-491 / LMD-9	823	0

7	*S. pneumoniae* serovar 19F, strain G54<br>*S. suis* 98HAH33<br>*S. pneumoniae* TIGR4 / ATCC BAA-334<br>*S. pyogenes* serovar M3, strain ATCC BAA-595<br>*S. pneumoniae* serovar 2, strain NCTC 7466<br>*S. pyogenes* serovar M12, strain MGAS2096<br>*S. pyogenes* serovar M28, strain MGAS6180<br>*S. pneumoniae* ATCC BAA-255 / R6<br>*S. agalactiae* serovar Ia, strain ATCC 27591<br>*S. gordonii* ATCC 35105 / CH1<br>*S. thermophilus* CNRZ 1066<br>*S. pneumoniae* Hungary19A-6<br>*S. pyogenes* serovar M12, strain MGAS9429	810	0
8	*S. suis* 98HAH33<br>*S. pyogenes* serovar M3, strain ATCC BAA-595<br>*S. pneumoniae* CGSP14<br>*S. suis* 05ZYH33<br>*S. agalactiae* serovar V, strain ATCC BAA-611<br>*S. pyogenes* serovar M2, strain MGAS10270<br>*S. pyogenes* serovar M4, strain MGAS10750<br>*S. pyogenes* serovar M3, strain SSI-1<br>*S. pyogenes* serovar M1, strain ATCC 700294<br>*S. pyogenes* serovar M6, strain ATCC BAA-946)<br>*S. pyogenes* serovar M12, strain MGAS9429<br>*S. pyogenes* serovar M18, strain MGAS8232<br>*S. thermophilus* ATCC BAA-491 / LMD-9	806	0
9	*S. pneumoniae* serovar 19F, strain G54<br>*S. equi* MGCS10565<br>*S. suis* 05ZYH33<br>*S. pneumoniae* CGSP14<br>*S. pyogenes* serovar M12, strain MGAS2096<br>*S. pyogenes* serovar M1, strain ATCC BAA-947<br>*S. agalactiae* serovar V, strain ATCC BAA-611<br>*S. pyogenes* serovar M28, strain MGAS6180<br>*S. agalactiae* serovar Ia, strain ATCC 27591<br>*S. agalactiae* serovar III, strain NEM316<br>*S. pyogenes* NZ131<br>*S. pyogenes* serovar M12, strain MGAS9429<br>*S. mutans* serovar c, strain ATCC 700610	851	0

10	*S. suis* 98HAH33<br>*S. sanguinis* SK36<br>*S. thermophilus* ATCC BAA-250 / LMG 18311<br>*S. pneumoniae* serovar 2, strain NCTC 7466<br>*S. suis* 05ZYH33<br>*S. pyogenes* serovar M1, strain ATCC BAA-947<br>*S. agalactiae* serovar V, strain ATCC BAA-611<br>*S. pyogenes* serovar M28, strain MGAS6180<br>*S. gordonii* ATCC 35105 / CH1<br>*S. thermophilus* CNRZ 1066<br>*S. pneumoniae* Hungary19A-6<br>*S. pyogenes* serovar M6, strain ATCC BAA-946)<br>*S. thermophilus* ATCC BAA-491 / LMD-9	788	0
11	*S. suis* 98HAH33<br>*S. sanguinis* SK36<br>*S. pyogenes* serovar M3, strain ATCC BAA-595<br>*S. pneumoniae* serovar 2, strain NCTC 7466<br>*S. pyogenes* serovar M12, strain MGAS2096<br>*S. pyogenes* serovar M28, strain MGAS6180<br>*S. pyogenes* serovar M2, strain MGAS10270<br>*S. gordonii* ATCC 35105 / CH1<br>*S. agalactiae* serovar Ia, strain ATCC 27591<br>*S. agalactiae* serovar III, strain NEM316<br>*S. thermophilus* CNRZ 1066<br>*S. pyogenes* serovar M6, strain ATCC BAA-946)<br>*S. thermophilus* ATCC BAA-491 / LMD-9	805	0
12	*S. suis* 98HAH33<br>*S. pneumoniae* serovar 19F, strain G54<br>*S. thermophilus* ATCC BAA-250 / LMG 18311<br>*S. pyogenes* serovar M3, strain ATCC BAA-595<br>*S. pneumoniae* serovar 2, strain NCTC 7466<br>*S. suis* 05ZYH33<br>*S. pyogenes* serovar M3, strain SSI-1<br>*S. pneumoniae* ATCC BAA-255 / R6<br>*S. agalactiae* serovar Ia, strain ATCC 27591<br>*S. pyogenes* serovar M1, strain ATCC 700294<br>*S. agalactiae* serovar III, strain NEM316<br>*S. pyogenes* NZ131<br>*S. mutans* serovar c, strain ATCC 700610	805	0

13	*S. sanguinis* SK36<br>*S. pneumoniae* serovar 19F, strain G54<br>*S. pneumoniae* TIGR4 / ATCC BAA-334<br>*S. pyogenes* serovar M3, strain ATCC BAA-595<br>*S. pneumoniae* serovar 2, strain NCTC 7466<br>*S. pneumoniae* CGSP14<br>*S. agalactiae* serovar V, strain ATCC BAA-611<br>*S. gordonii* ATCC 35105 / CH1<br>*S. pyogenes* serovar M1, strain ATCC 700294<br>*S. pyogenes* NZ131<br>*S. pneumoniae* Hungary19A-6<br>*S. pyogenes* serovar M6, strain ATCC BAA-946)<br>*S. thermophilus* ATCC BAA-491 / LMD-9	800	0
14	*S. suis* 98HAH33<br>*S. pneumoniae* TIGR4 / ATCC BAA-334<br>*S. thermophilus* ATCC BAA-250 / LMG 18311<br>*S. equi* MGCS10565<br>*S. pneumoniae* serovar 2, strain NCTC 7466<br>*S. pneumoniae* CGSP14<br>*S. pyogenes* serovar M12, strain MGAS2096<br>*S. pyogenes* serovar M28, strain MGAS6180<br>*S. pneumoniae* ATCC BAA-255 / R6<br>*S. pyogenes* serovar M1, strain ATCC 700294<br>*S. agalactiae* serovar III, strain NEM316<br>*S. pyogenes* serovar M5, strain Manfredo<br>*S. thermophilus* ATCC BAA-491 / LMD-9	778	0
15	*S. pneumoniae* TIGR4 / ATCC BAA-334<br>*S. equi* MGCS10565<br>*S. pneumoniae* serovar 2, strain NCTC 7466<br>*S. pyogenes* serovar M12, strain MGAS2096<br>*S. pyogenes* serovar M1, strain ATCC BAA-947<br>*S. pyogenes* serovar M28, strain MGAS6180<br>*S. pyogenes* serovar M4, strain MGAS10750<br>*S. pyogenes* serovar M2, strain MGAS10270<br>*S. agalactiae* serovar Ia, strain ATCC 27591<br>*S. pyogenes* serovar M1, strain ATCC 700294<br>*S. pyogenes* serovar M5, strain Manfredo<br>*S. pyogenes* serovar M12, strain MGAS9429<br>*S. thermophilus* ATCC BAA-491 / LMD-9	807	0

16	*S. suis* 98HAH33<br>*S. pneumoniae* serovar 2, strain NCTC 7466<br>*S. pneumoniae* CGSP14<br>*S. agalactiae* serovar V, strain ATCC BAA-611<br>*S. pyogenes* serovar M1, strain ATCC BAA-947<br>*S. pyogenes* serovar M12, strain MGAS2096<br>*S. pyogenes* serovar M4, strain MGAS10750<br>*S. pneumoniae* ATCC BAA-255 / R6<br>*S. gordonii* ATCC 35105 / CH1<br>*S. pyogenes* NZ131<br>*S. thermophilus* CNRZ 1066<br>*S. pyogenes* serovar M12, strain MGAS9429<br>*S. mutans* serovar c, strain ATCC 700610	797	0
17	*S. pneumoniae* TIGR4 / ATCC BAA-334<br>*S. pyogenes* serovar M3, strain ATCC BAA-595<br>*S. equi* MGCS10565<br>*S. pneumoniae* serovar 2, strain NCTC 7466<br>*S. pyogenes* serovar M1, strain ATCC BAA-947<br>*S. pyogenes* serovar M28, strain MGAS6180<br>*S. pyogenes* serovar M2, strain MGAS10270<br>*S. pyogenes* serovar M4, strain MGAS10750<br>*S. pyogenes* serovar M3, strain SSI-1<br>*S. agalactiae* serovar III, strain NEM316<br>*S. thermophilus* CNRZ 1066<br>*S. pyogenes* serovar M18, strain MGAS8232<br>*S. pyogenes* serovar M6, strain ATCC BAA-946)	823	0
18	*S. sanguinis* SK36<br>*S. pneumoniae* serovar 19F, strain G54<br>*S. suis* 98HAH33<br>*S. suis* 05ZYH33<br>*S. pneumoniae* CGSP14<br>*S. pyogenes* serovar M4, strain MGAS10750<br>*S. pneumoniae* ATCC BAA-255 / R6<br>*S. gordonii* ATCC 35105 / CH1<br>*S. pyogenes* NZ131<br>*S. thermophilus* CNRZ 1066<br>*S. pyogenes* serovar M18, strain MGAS8232<br>*S. pyogenes* serovar M5, strain Manfredo<br>*S. thermophilus* ATCC BAA-491 / LMD-9	807	0

19	*S. suis* 98HAH33<br>*S. thermophilus* ATCC BAA-250 / LMG 18311<br>*S. equi* MGCS10565<br>*S. suis* 05ZYH33<br>*S. pyogenes* serovar M1, strain ATCC BAA-947<br>*S. pyogenes* serovar M3, strain SSI-1<br>*S. pneumoniae* ATCC BAA-255 / R6<br>*S. agalactiae* serovar Ia, strain ATCC 27591<br>*S. agalactiae* serovar III, strain NEM316<br>*S. pyogenes* NZ131<br>*S. pneumoniae* Hungary19A-6<br>*S. pyogenes* serovar M5, strain Manfredo<br>*S. thermophilus* ATCC BAA-491 / LMD-9	783	0
20	*S. pneumoniae* TIGR4 / ATCC BAA-334<br>*S. thermophilus* ATCC BAA-250 / LMG 18311<br>*S. equi* MGCS10565<br>*S. pneumoniae* serovar 2, strain NCTC 7466<br>*S. pyogenes* serovar M1, strain ATCC BAA-947<br>*S. agalactiae* serovar V, strain ATCC BAA-611<br>*S. pyogenes* serovar M28, strain MGAS6180<br>*S. pneumoniae* ATCC BAA-255 / R6<br>*S. pyogenes* serovar M1, strain ATCC 700294<br>*S. pyogenes* NZ131<br>*S. thermophilus* CNRZ 1066<br>*S. pneumoniae* Hungary19A-6<br>*S. pyogenes* serovar M5, strain Manfredo	795	0
21	*S. sanguinis* SK36<br>*S. suis* 98HAH33<br>*S. pneumoniae* TIGR4 / ATCC BAA-334<br>*S. pyogenes* serovar M4, strain MGAS10750<br>*S. pyogenes* serovar M3, strain SSI-1<br>*S. gordonii* ATCC 35105 / CH1<br>*S. agalactiae* serovar Ia, strain ATCC 27591<br>*S. pyogenes* serovar M1, strain ATCC 700294<br>*S. agalactiae* serovar III, strain NEM316<br>*S. pyogenes* NZ131<br>*S. pyogenes* serovar M12, strain MGAS9429<br>*S. pyogenes* serovar M18, strain MGAS8232<br>*S. thermophilus* ATCC BAA-491 / LMD-9	799	0

22	*S. sanguinis* SK36<br>*S. suis* 98HAH33<br>*S. suis* 05ZYH33<br>*S. pneumoniae* CGSP14<br>*S. pyogenes* serovar M12, strain MGAS2096<br>*S. pyogenes* serovar M3, strain SSI-1<br>*S. agalactiae* serovar III, strain NEM316<br>*S. pyogenes* NZ131<br>*S. thermophilus* CNRZ 1066<br>*S. pyogenes* serovar M18, strain MGAS8232<br>*S. pyogenes* serovar M12, strain MGAS9429<br>*S. pneumoniae* Hungary19A-6<br>*S. mutans* serovar c, strain ATCC 700610	799	0
23	*S. pneumoniae* serovar 19F, strain G54<br>*S. pneumoniae* TIGR4 / ATCC BAA-334<br>*S. suis* 05ZYH33<br>*S. pyogenes* serovar M12, strain MGAS2096<br>*S. agalactiae* serovar V, strain ATCC BAA-611<br>*S. pyogenes* serovar M4, strain MGAS10750<br>*S. pyogenes* serovar M28, strain MGAS6180<br>*S. agalactiae* serovar Ia, strain ATCC 27591<br>*S. pyogenes* serovar M1, strain ATCC 700294<br>*S. pyogenes* serovar M18, strain MGAS8232<br>*S. pneumoniae* Hungary19A-6<br>*S. pyogenes* serovar M5, strain Manfredo<br>*S. pyogenes* serovar M6, strain ATCC BAA-946)	887	0
24	*S. sanguinis* SK36<br>*S. pneumoniae* serovar 2, strain NCTC 7466<br>*S. pneumoniae* CGSP14<br>*S. agalactiae* serovar V, strain ATCC BAA-611<br>*S. pyogenes* serovar M28, strain MGAS6180<br>*S. pyogenes* serovar M2, strain MGAS10270<br>*S. pyogenes* serovar M4, strain MGAS10750<br>*S. pyogenes* serovar M3, strain SSI-1<br>*S. agalactiae* serovar Ia, strain ATCC 27591<br>*S. pyogenes* NZ131<br>*S. pyogenes* serovar M12, strain MGAS9429<br>*S. pyogenes* serovar M5, strain Manfredo<br>*S. thermophilus* ATCC BAA-491 / LMD-9	823	0

25	*S. pneumoniae* serovar 19F, strain G54<br>*S. pneumoniae* TIGR4 / ATCC BAA-334<br>*S. pneumoniae* serovar 2, strain NCTC 7466<br>*S. agalactiae* serovar V, strain ATCC BAA-611<br>*S. pyogenes* serovar M28, strain MGAS6180<br>*S. pyogenes* serovar M2, strain MGAS10270<br>*S. pyogenes* serovar M4, strain MGAS10750<br>*S. gordonii* ATCC 35105 / CH1<br>*S. agalactiae* serovar Ia, strain ATCC 27591<br>*S. pyogenes* serovar M5, strain Manfredo<br>*S. pyogenes* serovar M6, strain ATCC BAA-946)<br>*S. pyogenes* serovar M18, strain MGAS8232<br>*S. mutans* serovar c, strain ATCC 700610	868	0
