# Supplementary material for: Analysis and comparison of the pan-genomic properties of sixteen well-characterized bacterial genera
Source: BMC Microbiol. 2010 Oct 13;10:258. doi: 10.1186/1471-2180-10-258 (PMC3020658; doi:10.1186/1471-2180-10-258)
Supplement: Additional file 5 — Complete list of random groups. These tables list the random groups used for the analysis whose results are summarized in Tables 3 and 4 of the main paper. The column heading NC indicates the number of proteins in that group's core proteome, while NU indicates the number of proteins found in the proteomes of all members of that group, but no other isolates from the same genus. [file 1471-2180-10-258-S5.ZIP › Streptococcus_2_isolates.pdf]

Random groups corresponding to *Streptococcus* species with 2 isolates.

#	Members of random group	N_C	N_U
1	*S. sanguinis* SK36<br>*S. pyogenes* serovar M4, strain MGAS10750	1089	0
2	*S. pneumoniae* serovar 19F, strain G54<br>*S. thermophilus* ATCC BAA-491 / LMD-9	1027	0
3	*S. pneumoniae* Hungary19A-6<br>*S. pyogenes* serovar M1, strain ATCC BAA-947	1065	0
4	*S. pneumoniae* TIGR4 / ATCC BAA-334<br>*S. thermophilus* ATCC BAA-491 / LMD-9	1023	0
5	*S. pyogenes* serovar M4, strain MGAS10750<br>*S. mutans* serovar c, strain ATCC 700610	1055	1
6	*S. equi* MGCS10565<br>*S. pyogenes* serovar M12, strain MGAS2096	1218	0
7	*S. pneumoniae* CGSP14<br>*S. pyogenes* serovar M4, strain MGAS10750	1075	0
8	*S. pyogenes* serovar M18, strain MGAS8232<br>*S. mutans* serovar c, strain ATCC 700610	1030	0
9	*S. pyogenes* serovar M5, strain Manfredo<br>*S. suis* 05ZYH33	1057	0
10	*S. pneumoniae* ATCC BAA-255 / R6<br>*S. pyogenes* serovar M2, strain MGAS10270	1063	0
11	*S. gordonii* ATCC 35105 / CH1<br>*S. pyogenes* serovar M28, strain MGAS6180	1086	0
12	*S. pneumoniae* serovar 19F, strain G54<br>*S. pyogenes* serovar M12, strain MGAS2096	1065	0
13	*S. agalactiae* serovar III, strain NEM316<br>*S. pyogenes* serovar M12, strain MGAS2096	1193	0
14	*S. agalactiae* serovar Ia, strain ATCC 27591<br>*S. pneumoniae* serovar 2, strain NCTC 7466	1137	0
15	*S. agalactiae* serovar III, strain NEM316<br>*S. mutans* serovar c, strain ATCC 700610	1146	1
16	*S. agalactiae* serovar Ia, strain ATCC 27591<br>*S. pyogenes* serovar M2, strain MGAS10270	1232	0
17	*S. pyogenes* serovar M28, strain MGAS6180<br>*S. mutans* serovar c, strain ATCC 700610	1064	0
18	*S. pyogenes* serovar M3, strain SSI-1<br>*S. agalactiae* serovar V, strain ATCC BAA-611	1206	0
19	*S. pneumoniae* ATCC BAA-255 / R6<br>*S. pyogenes* serovar M18, strain MGAS8232	1051	0
20	*S. equi* MGCS10565<br>*S. pyogenes* NZ131	1230	0
21	*S. suis* 05ZYH33<br>*S. pyogenes* serovar M6, strain ATCC BAA-946)	1069	0
22	*S. sanguinis* SK36<br>*S. pyogenes* NZ131	1081	0
23	*S. pneumoniae* serovar 19F, strain G54<br>*S. thermophilus* ATCC BAA-250 / LMG 18311	1037	0
24	*S. pyogenes* NZ131<br>*S. thermophilus* ATCC BAA-491 / LMD-9	946	0
25	*S. pyogenes* serovar M1, strain ATCC 700294<br>*S. thermophilus* CNRZ 1066	951	0
