# Supplementary material for: Analysis and comparison of the pan-genomic properties of sixteen well-characterized bacterial genera
Source: BMC Microbiol. 2010 Oct 13;10:258. doi: 10.1186/1471-2180-10-258 (PMC3020658; doi:10.1186/1471-2180-10-258)
Supplement: Additional file 5 — Complete list of random groups. These tables list the random groups used for the analysis whose results are summarized in Tables 3 and 4 of the main paper. The column heading NC indicates the number of proteins in that group's core proteome, while NU indicates the number of proteins found in the proteomes of all members of that group, but no other isolates from the same genus. [file 1471-2180-10-258-S5.ZIP › Streptococcus_3_isolates.pdf]

Random groups corresponding to *Streptococcus* species with 3 isolates.

#	Members of random group	N_C	N_U
1	*S. pneumoniae* ATCC BAA-255 / R6<br>*S. pneumoniae* CGSP14<br>*S. pyogenes* serovar M4, strain MGAS10750	1051	0
2	*S. agalactiae* serovar Ia, strain ATCC 27591<br>*S. suis* 05ZYH33<br>*S. thermophilus* ATCC BAA-491 / LMD-9	938	0
3	*S. pyogenes* serovar M3, strain SSI-1<br>*S. pneumoniae* serovar 19F, strain G54<br>*S. agalactiae* serovar Ia, strain ATCC 27591	987	0
4	*S. sanguinis* SK36<br>*S. pneumoniae* CGSP14<br>*S. mutans* serovar c, strain ATCC 700610	1069	0
5	*S. pneumoniae* serovar 19F, strain G54<br>*S. agalactiae* serovar Ia, strain ATCC 27591<br>*S. thermophilus* ATCC BAA-491 / LMD-9	947	0
6	*S. pneumoniae* ATCC BAA-255 / R6<br>*S. agalactiae* serovar III, strain NEM316<br>*S. pyogenes* serovar M6, strain ATCC BAA-946)	991	0
7	*S. agalactiae* serovar III, strain NEM316<br>*S. pyogenes* serovar M6, strain ATCC BAA-946)<br>*S. pyogenes* serovar M12, strain MGAS2096	1170	0
8	*S. pneumoniae* TIGR4 / ATCC BAA-334<br>*S. equi* MGCS10565<br>*S. pyogenes* NZ131	988	0
9	*S. pneumoniae* ATCC BAA-255 / R6<br>*S. pyogenes* serovar M3, strain ATCC BAA-595<br>*S. agalactiae* serovar III, strain NEM316	998	0
10	*S. pyogenes* serovar M3, strain ATCC BAA-595<br>*S. agalactiae* serovar III, strain NEM316<br>*S. pyogenes* serovar M18, strain MGAS8232	1170	0
11	*S. pneumoniae* TIGR4 / ATCC BAA-334<br>*S. pyogenes* serovar M3, strain ATCC BAA-595<br>*S. pyogenes* NZ131	1051	0
12	*S. pneumoniae* Hungary19A-6<br>*S. pyogenes* serovar M12, strain MGAS2096<br>*S. pyogenes* serovar M2, strain MGAS10270	1065	0
13	*S. pyogenes* serovar M3, strain SSI-1<br>*S. thermophilus* CNRZ 1066<br>*S. pyogenes* serovar M18, strain MGAS8232	942	0
14	*S. thermophilus* CNRZ 1066<br>*S. pyogenes* serovar M6, strain ATCC BAA-946)<br>*S. thermophilus* ATCC BAA-491 / LMD-9	923	0
15	*S. suis* 98HAH33<br>*S. agalactiae* serovar Ia, strain ATCC 27591<br>*S. thermophilus* ATCC BAA-250 / LMG 18311	951	0
16	*S. suis* 98HAH33<br>*S. pyogenes* NZ131<br>*S. agalactiae* serovar V, strain ATCC BAA-611	998	0

17	*S. pyogenes* serovar M3, strain ATCC BAA-595<br>*S. pneumoniae* serovar 2, strain NCTC 7466<br>*S. pyogenes* serovar M5, strain Manfredo	1033	0
18	*S. pneumoniae* ATCC BAA-255 / R6<br>*S. suis* 98HAH33<br>*S. equi* MGCS10565	1009	0
19	*S. suis* 98HAH33<br>*S. gordonii* ATCC 35105 / CH1<br>*S. agalactiae* serovar III, strain NEM316	1043	0
20	*S. agalactiae* serovar Ia, strain ATCC 27591<br>*S. pyogenes* serovar M4, strain MGAS10750<br>*S. mutans* serovar c, strain ATCC 700610	989	0
21	*S. gordonii* ATCC 35105 / CH1<br>*S. pneumoniae* CGSP14<br>*S. agalactiae* serovar V, strain ATCC BAA-611	1045	0
22	*S. suis* 98HAH33<br>*S. pyogenes* serovar M12, strain MGAS9429<br>*S. pyogenes* serovar M5, strain Manfredo	1051	0
23	*S. pneumoniae* ATCC BAA-255 / R6<br>*S. agalactiae* serovar Ia, strain ATCC 27591<br>*S. pneumoniae* TIGR4 / ATCC BAA-334	1121	0
24	*S. sanguinis* SK36<br>*S. thermophilus* CNRZ 1066<br>*S. pyogenes* serovar M6, strain ATCC BAA-946)	903	0
25	*S. suis* 98HAH33<br>*S. pyogenes* serovar M5, strain Manfredo<br>*S. pyogenes* serovar M2, strain MGAS10270	1051	0
