# Supplementary material for: Analysis and comparison of the pan-genomic properties of sixteen well-characterized bacterial genera
Source: BMC Microbiol. 2010 Oct 13;10:258. doi: 10.1186/1471-2180-10-258 (PMC3020658; doi:10.1186/1471-2180-10-258)
Supplement: Additional file 5 — Complete list of random groups. These tables list the random groups used for the analysis whose results are summarized in Tables 3 and 4 of the main paper. The column heading NC indicates the number of proteins in that group's core proteome, while NU indicates the number of proteins found in the proteomes of all members of that group, but no other isolates from the same genus. [file 1471-2180-10-258-S5.ZIP › Streptococcus_6_isolates.pdf]

Random groups corresponding to *Streptococcus* species with 6 isolates.

#	Members of random group	N_C	N_U
1	*S. sanguinis* SK36<br>*S. pneumoniae* ATCC BAA-255 / R6<br>*S. agalactiae* serovar Ia, strain ATCC 27591<br>*S. agalactiae* serovar III, strain NEM316<br>*S. suis* 05ZYH33<br>*S. thermophilus* ATCC BAA-491 / LMD-9	881	0
2	*S. pneumoniae* TIGR4 / ATCC BAA-334<br>*S. thermophilus* ATCC BAA-250 / LMG 18311<br>*S. pyogenes* serovar M6, strain ATCC BAA-946)<br>*S. pyogenes* serovar M5, strain Manfredo<br>*S. pneumoniae* CGSP14<br>*S. agalactiae* serovar V, strain ATCC BAA-611	839	0
3	*S. pyogenes* serovar M3, strain SSI-1<br>*S. pyogenes* NZ131<br>*S. pneumoniae* CGSP14<br>*S. pyogenes* serovar M18, strain MGAS8232<br>*S. suis* 05ZYH33<br>*S. thermophilus* ATCC BAA-491 / LMD-9	832	0
4	*S. gordonii* ATCC 35105 / CH1<br>*S. pyogenes* serovar M1, strain ATCC 700294<br>*S. pyogenes* serovar M12, strain MGAS9429<br>*S. pyogenes* serovar M6, strain ATCC BAA-946)<br>*S. pneumoniae* Hungary19A-6<br>*S. pyogenes* serovar M12, strain MGAS2096	972	0
5	*S. pneumoniae* serovar 19F, strain G54<br>*S. agalactiae* serovar Ia, strain ATCC 27591<br>*S. pyogenes* serovar M1, strain ATCC 700294<br>*S. agalactiae* serovar III, strain NEM316<br>*S. pyogenes* NZ131<br>*S. pneumoniae* Hungary19A-6	957	0
6	*S. equi* MGCS10565<br>*S. pyogenes* serovar M18, strain MGAS8232<br>*S. pneumoniae* Hungary19A-6<br>*S. pyogenes* serovar M12, strain MGAS2096<br>*S. pyogenes* serovar M4, strain MGAS10750<br>*S. pyogenes* serovar M28, strain MGAS6180	974	0
7	*S. pyogenes* serovar M1, strain ATCC 700294<br>*S. pyogenes* serovar M5, strain Manfredo<br>*S. pyogenes* serovar M18, strain MGAS8232<br>*S. pyogenes* serovar M1, strain ATCC BAA-947<br>*S. thermophilus* ATCC BAA-491 / LMD-9<br>*S. pyogenes* serovar M4, strain MGAS10750	906	0
8	*S. sanguinis* SK36<br>*S. pneumoniae* serovar 19F, strain G54<br>*S. pneumoniae* serovar 2, strain NCTC 7466<br>*S. pneumoniae* Hungary19A-6<br>*S. pyogenes* serovar M12, strain MGAS9429<br>*S. pyogenes* serovar M2, strain MGAS10270	967	0

9	*S. pneumoniae* ATCC BAA-255 / R6<br>*S. pyogenes* serovar M3, strain ATCC BAA-595<br>*S. thermophilus* CNRZ 1066<br>*S. pyogenes* serovar M6, strain ATCC BAA-946)<br>*S. pyogenes* serovar M1, strain ATCC BAA-947<br>*S. pyogenes* serovar M4, strain MGAS10750	882	0
10	*S. pneumoniae* TIGR4 / ATCC BAA-334<br>*S. pyogenes* serovar M1, strain ATCC 700294<br>*S. pyogenes* serovar M5, strain Manfredo<br>*S. pyogenes* serovar M18, strain MGAS8232<br>*S. agalactiae* serovar V, strain ATCC BAA-611<br>*S. pyogenes* serovar M2, strain MGAS10270	958	0
11	*S. pyogenes* serovar M1, strain ATCC 700294<br>*S. agalactiae* serovar III, strain NEM316<br>*S. equi* MGCS10565<br>*S. pyogenes* NZ131<br>*S. pyogenes* serovar M28, strain MGAS6180<br>*S. mutans* serovar c, strain ATCC 700610	945	0
12	*S. sanguinis* SK36<br>*S. agalactiae* serovar Ia, strain ATCC 27591<br>*S. equi* MGCS10565<br>*S. pyogenes* serovar M12, strain MGAS9429<br>*S. pyogenes* serovar M18, strain MGAS8232<br>*S. pyogenes* serovar M4, strain MGAS10750	959	0
13	*S. pneumoniae* ATCC BAA-255 / R6<br>*S. pyogenes* serovar M3, strain SSI-1<br>*S. pneumoniae* TIGR4 / ATCC BAA-334<br>*S. pyogenes* serovar M3, strain ATCC BAA-595<br>*S. pyogenes* serovar M12, strain MGAS9429<br>*S. pyogenes* serovar M5, strain Manfredo	1022	0
14	*S. pyogenes* serovar M3, strain SSI-1<br>*S. pyogenes* serovar M3, strain ATCC BAA-595<br>*S. pneumoniae* serovar 2, strain NCTC 7466<br>*S. thermophilus* CNRZ 1066<br>*S. suis* 05ZYH33<br>*S. pyogenes* serovar M28, strain MGAS6180	850	0
15	*S. pneumoniae* ATCC BAA-255 / R6<br>*S. pyogenes* serovar M1, strain ATCC 700294<br>*S. agalactiae* serovar III, strain NEM316<br>*S. pyogenes* serovar M5, strain Manfredo<br>*S. pyogenes* serovar M1, strain ATCC BAA-947<br>*S. pyogenes* serovar M28, strain MGAS6180	973	0
16	*S. agalactiae* serovar Ia, strain ATCC 27591<br>*S. thermophilus* ATCC BAA-250 / LMG 18311<br>*S. pneumoniae* serovar 2, strain NCTC 7466<br>*S. agalactiae* serovar V, strain ATCC BAA-611<br>*S. pyogenes* serovar M4, strain MGAS10750<br>*S. pyogenes* serovar M28, strain MGAS6180	859	0

17	*S. pneumoniae* ATCC BAA-255 / R6<br>*S. gordonii* ATCC 35105 / CH1<br>*S. agalactiae* serovar III, strain NEM316<br>*S. pneumoniae* Hungary19A-6<br>*S. thermophilus* ATCC BAA-491 / LMD-9<br>*S. pyogenes* serovar M2, strain MGAS10270	834	0
18	*S. pneumoniae* serovar 19F, strain G54<br>*S. pneumoniae* ATCC BAA-255 / R6<br>*S. sanguinis* SK36<br>*S. gordonii* ATCC 35105 / CH1<br>*S. pneumoniae* TIGR4 / ATCC BAA-334<br>*S. pyogenes* serovar M12, strain MGAS2096	955	0
19	*S. sanguinis* SK36<br>*S. suis* 98HAH33<br>*S. pneumoniae* TIGR4 / ATCC BAA-334<br>*S. pyogenes* NZ131<br>*S. pneumoniae* CGSP14<br>*S. pyogenes* serovar M6, strain ATCC BAA-946)	931	0
20	*S. pyogenes* serovar M3, strain ATCC BAA-595<br>*S. pyogenes* serovar M1, strain ATCC 700294<br>*S. agalactiae* serovar III, strain NEM316<br>*S. suis* 05ZYH33<br>*S. pyogenes* serovar M12, strain MGAS2096<br>*S. pyogenes* serovar M2, strain MGAS10270	991	0
21	*S. pyogenes* serovar M1, strain ATCC 700294<br>*S. thermophilus* ATCC BAA-250 / LMG 18311<br>*S. pyogenes* NZ131<br>*S. pyogenes* serovar M18, strain MGAS8232<br>*S. pyogenes* serovar M4, strain MGAS10750<br>*S. pyogenes* serovar M2, strain MGAS10270	931	0
22	*S. pyogenes* serovar M3, strain SSI-1<br>*S. sanguinis* SK36<br>*S. agalactiae* serovar Ia, strain ATCC 27591<br>*S. pyogenes* NZ131<br>*S. pneumoniae* serovar 2, strain NCTC 7466<br>*S. pyogenes* serovar M4, strain MGAS10750	950	0
23	*S. pyogenes* serovar M3, strain SSI-1<br>*S. suis* 98HAH33<br>*S. pyogenes* serovar M3, strain ATCC BAA-595<br>*S. thermophilus* ATCC BAA-250 / LMG 18311<br>*S. pneumoniae* serovar 2, strain NCTC 7466<br>*S. pneumoniae* Hungary19A-6	832	0
24	*S. pneumoniae* TIGR4 / ATCC BAA-334<br>*S. pyogenes* serovar M1, strain ATCC 700294<br>*S. pyogenes* NZ131<br>*S. pneumoniae* serovar 2, strain NCTC 7466<br>*S. pyogenes* serovar M6, strain ATCC BAA-946)<br>*S. pyogenes* serovar M12, strain MGAS9429	1014	0

25	*S. sanguinis* SK36<br>*S. pneumoniae* ATCC BAA-255 / R6<br>*S. thermophilus* ATCC BAA-250 / LMG 18311<br>*S. pneumoniae* serovar 2, strain NCTC 7466<br>*S. pyogenes* serovar M12, strain MGAS2096<br>*S. agalactiae* serovar V, strain ATCC BAA-611	838	0
