# Supplementary material for: Analysis and comparison of the pan-genomic properties of sixteen well-characterized bacterial genera
Source: BMC Microbiol. 2010 Oct 13;10:258. doi: 10.1186/1471-2180-10-258 (PMC3020658; doi:10.1186/1471-2180-10-258)
Supplement: Additional file 5 — Complete list of random groups. These tables list the random groups used for the analysis whose results are summarized in Tables 3 and 4 of the main paper. The column heading NC indicates the number of proteins in that group's core proteome, while NU indicates the number of proteins found in the proteomes of all members of that group, but no other isolates from the same genus. [file 1471-2180-10-258-S5.ZIP › Vibrio_2_isolates.pdf]

Random groups corresponding to *Vibrio* species with 2 isolates.

#	Members of random group	N_C	N_U
1	*V. cholerae* serovar O1, strain ATCC 39315<br>*V. vulnificus* YJ016	2802	9
2	*V. vulnificus* YJ016<br>*V. parahaemolyticus* RIMD 2210633	3282	20
3	*V. cholerae* serovar O1, strain ATCC 39541<br>*V. fischeri* MJ11	2367	1
4	*V. harveyi* ATCC BAA-1116 / BB120<br>*V. fischeri* MJ11	2706	25
5	*V. harveyi* ATCC BAA-1116 / BB120<br>*V. fischeri* ATCC 700601 / ES114	2672	15
6	*V. fischeri* MJ11<br>*V. vulnificus* YJ016	2688	16
7	*V. fischeri* ATCC 700601 / ES114<br>*V. parahaemolyticus* RIMD 2210633	2689	0
8	*V. vulnificus* CMCP6<br>*V. fischeri* ATCC 700601 / ES114	2650	2
9	*V. vulnificus* CMCP6<br>*V. cholerae* serovar O1, strain ATCC 39541	2791	1
10	*V. vulnificus* YJ016<br>*V. fischeri* ATCC 700601 / ES114	2675	7
11	*V. vulnificus* CMCP6<br>*V. parahaemolyticus* RIMD 2210633	3252	22
12	*V. fischeri* MJ11<br>*V. cholerae* serovar O1, strain ATCC 39315	2345	0
13	*V. cholerae* serovar O1, strain ATCC 39315<br>*V. fischeri* ATCC 700601 / ES114	2345	0
14	*V. cholerae* serovar O1, strain ATCC 39541<br>*V. fischeri* ATCC 700601 / ES114	2381	5
15	*V. cholerae* serovar O1, strain ATCC 39315<br>*V. parahaemolyticus* RIMD 2210633	2677	1
16	*V. vulnificus* CMCP6<br>*V. fischeri* MJ11	2664	4
17	*V. cholerae* serovar O1, strain ATCC 39541<br>*V. parahaemolyticus* RIMD 2210633	2704	0
18	*V. fischeri* MJ11<br>*V. parahaemolyticus* RIMD 2210633	2732	9
19	*V. harveyi* ATCC BAA-1116 / BB120<br>*V. cholerae* serovar O1, strain ATCC 39315	2639	2
20	*V. harveyi* ATCC BAA-1116 / BB120<br>*V. vulnificus* YJ016	3132	22
21	*V. cholerae* serovar O1, strain ATCC 39541<br>*V. harveyi* ATCC BAA-1116 / BB120	2689	13
22	*V. harveyi* ATCC BAA-1116 / BB120<br>*V. parahaemolyticus* RIMD 2210633	3509	310
23	*V. cholerae* serovar O1, strain ATCC 39541<br>*V. vulnificus* YJ016	2823	6
24	*V. vulnificus* CMCP6<br>*V. cholerae* serovar O1, strain ATCC 39315	2768	3
25	*V. vulnificus* CMCP6<br>*V. harveyi* ATCC BAA-1116 / BB120	3118	23
