# Supplementary material for: Analysis and comparison of the pan-genomic properties of sixteen well-characterized bacterial genera
Source: BMC Microbiol. 2010 Oct 13;10:258. doi: 10.1186/1471-2180-10-258 (PMC3020658; doi:10.1186/1471-2180-10-258)
Supplement: Additional file 5 — Complete list of random groups. These tables list the random groups used for the analysis whose results are summarized in Tables 3 and 4 of the main paper. The column heading NC indicates the number of proteins in that group's core proteome, while NU indicates the number of proteins found in the proteomes of all members of that group, but no other isolates from the same genus. [file 1471-2180-10-258-S5.ZIP › Xanthomonas_3_isolates.pdf]

Random groups corresponding to *Xanthomonas* species with 3 isolates.

#	Members of random group	N_C	N_U
1	*X. oryzae* pathovar oryzae, strain PXO99A<br>*X. axonopodis* pathovar citri, strain 306<br>*X. campestris* pathovar campestris, strain B100	2783	0
2	*X. oryzae* pathovar oryzae, strain PXO99A<br>*X. campestris* pathovar campestris, strain 8004<br>*X. oryzae* oryzae, strain KXO85 / KACC10331	2766	0
3	*X. oryzae* pathovar oryzae, strain PXO99A<br>*X. campestris* campestris, strain ATCC 33913<br>*X. campestris* pathovar vesicatoria, strain 85-10	2818	0
4	*X. axonopodis* pathovar citri, strain 306<br>*X. campestris* pathovar campestris, strain B100<br>*X. oryzae* pathovar oryzae, strain MAFF 311018	2822	0
5	*X. oryzae* pathovar oryzae, strain PXO99A<br>*X. campestris* pathovar campestris, strain 8004<br>*X. campestris* pathovar campestris, strain B100	2822	0
6	*X. campestris* campestris, strain ATCC 33913<br>*X. campestris* pathovar campestris, strain 8004<br>*X. oryzae* oryzae, strain KXO85 / KACC10331	2806	0
7	*X. campestris* pathovar vesicatoria, strain 85-10<br>*X. oryzae* oryzae, strain KXO85 / KACC10331<br>*X. campestris* pathovar campestris, strain B100	2771	0
8	*X. axonopodis* pathovar citri, strain 306<br>*X. campestris* pathovar vesicatoria, strain 85-10<br>*X. campestris* pathovar campestris, strain B100	3335	8
9	*X. campestris* pathovar campestris, strain 8004<br>*X. campestris* pathovar vesicatoria, strain 85-10<br>*X. axonopodis* pathovar citri, strain 306	3339	4
10	*X. campestris* campestris, strain ATCC 33913<br>*X. axonopodis* pathovar citri, strain 306<br>*X. campestris* pathovar vesicatoria, strain 85-10	3310	3
11	*X. campestris* pathovar campestris, strain 8004<br>*X. campestris* pathovar campestris, strain B100<br>*X. oryzae* pathovar oryzae, strain MAFF 311018	2871	0
12	*X. campestris* campestris, strain ATCC 33913<br>*X. oryzae* oryzae, strain KXO85 / KACC10331<br>*X. campestris* pathovar campestris, strain B100	2789	0
13	*X. oryzae* pathovar oryzae, strain PXO99A<br>*X. oryzae* oryzae, strain KXO85 / KACC10331<br>*X. campestris* pathovar campestris, strain B100	2749	0
14	*X. campestris* pathovar campestris, strain 8004<br>*X. axonopodis* pathovar citri, strain 306<br>*X. oryzae* pathovar oryzae, strain MAFF 311018	2824	0
15	*X. campestris* campestris, strain ATCC 33913<br>*X. axonopodis* pathovar citri, strain 306<br>*X. campestris* pathovar campestris, strain B100	3334	1
16	*X. campestris* pathovar campestris, strain 8004<br>*X. oryzae* oryzae, strain KXO85 / KACC10331<br>*X. campestris* pathovar campestris, strain B100	2788	0

17	*X. campestris* campestris, strain ATCC 33913<br>*X. campestris* pathovar vesicatoria, strain 85-10<br>*X. oryzae* oryzae, strain KXO85 / KACC10331	2786	0
18	*X. campestris* pathovar campestris, strain 8004<br>*X. campestris* campestris, strain ATCC 33913<br>*X. axonopodis* pathovar citri, strain 306	3358	5
19	*X. axonopodis* pathovar citri, strain 306<br>*X. oryzae* oryzae, strain KXO85 / KACC10331<br>*X. oryzae* pathovar oryzae, strain MAFF 311018	2888	2
20	*X. oryzae* pathovar oryzae, strain PXO99A<br>*X. campestris* campestris, strain ATCC 33913<br>*X. campestris* pathovar campestris, strain B100	2824	0
21	*X. axonopodis* pathovar citri, strain 306<br>*X. oryzae* oryzae, strain KXO85 / KACC10331<br>*X. campestris* pathovar campestris, strain B100	2747	0
22	*X. oryzae* pathovar oryzae, strain PXO99A<br>*X. campestris* campestris, strain ATCC 33913<br>*X. campestris* pathovar campestris, strain 8004	2847	0
23	*X. campestris* pathovar campestris, strain 8004<br>*X. campestris* pathovar vesicatoria, strain 85-10<br>*X. oryzae* pathovar oryzae, strain MAFF 311018	2859	0
24	*X. campestris* campestris, strain ATCC 33913<br>*X. campestris* pathovar vesicatoria, strain 85-10<br>*X. oryzae* pathovar oryzae, strain MAFF 311018	2864	1
25	*X. oryzae* pathovar oryzae, strain PXO99A<br>*X. campestris* pathovar campestris, strain 8004<br>*X. axonopodis* pathovar citri, strain 306	2786	0
