# Supplementary material for: Analysis and comparison of the pan-genomic properties of sixteen well-characterized bacterial genera
Source: BMC Microbiol. 2010 Oct 13;10:258. doi: 10.1186/1471-2180-10-258 (PMC3020658; doi:10.1186/1471-2180-10-258)
Supplement: Additional file 5 — Complete list of random groups. These tables list the random groups used for the analysis whose results are summarized in Tables 3 and 4 of the main paper. The column heading NC indicates the number of proteins in that group's core proteome, while NU indicates the number of proteins found in the proteomes of all members of that group, but no other isolates from the same genus. [file 1471-2180-10-258-S5.ZIP › Xanthomonas_4_isolates.pdf]

Random groups corresponding to *Xanthomonas* species with 4 isolates.

#	Members of random group	N_C	N_U
1	*X. campestris* pathovar vesicatoria, strain 85-10<br>*X. axonopodis* pathovar citri, strain 306<br>*X. oryzae* oryzae, strain KXO85 / KACC10331<br>*X. oryzae* pathovar oryzae, strain MAFF 311018	2832	2
2	*X. campestris* campestris, strain ATCC 33913<br>*X. campestris* pathovar campestris, strain 8004<br>*X. oryzae* oryzae, strain KXO85 / KACC10331<br>*X. campestris* pathovar campestris, strain B100	2784	0
3	*X. oryzae* pathovar oryzae, strain PXO99A<br>*X. oryzae* oryzae, strain KXO85 / KACC10331<br>*X. campestris* pathovar campestris, strain B100<br>*X. oryzae* pathovar oryzae, strain MAFF 311018	2740	4
4	*X. oryzae* pathovar oryzae, strain PXO99A<br>*X. campestris* pathovar campestris, strain 8004<br>*X. campestris* pathovar campestris, strain B100<br>*X. oryzae* pathovar oryzae, strain MAFF 311018	2800	0
5	*X. oryzae* pathovar oryzae, strain PXO99A<br>*X. axonopodis* pathovar citri, strain 306<br>*X. oryzae* oryzae, strain KXO85 / KACC10331<br>*X. campestris* pathovar campestris, strain B100	2695	0
6	*X. oryzae* pathovar oryzae, strain PXO99A<br>*X. campestris* pathovar campestris, strain 8004<br>*X. axonopodis* pathovar citri, strain 306<br>*X. campestris* pathovar campestris, strain B100	2772	0
7	*X. campestris* pathovar campestris, strain 8004<br>*X. oryzae* oryzae, strain KXO85 / KACC10331<br>*X. campestris* pathovar campestris, strain B100<br>*X. oryzae* pathovar oryzae, strain MAFF 311018	2773	0
8	*X. campestris* pathovar campestris, strain 8004<br>*X. campestris* pathovar vesicatoria, strain 85-10<br>*X. axonopodis* pathovar citri, strain 306<br>*X. campestris* pathovar campestris, strain B100	3313	33
9	*X. campestris* pathovar vesicatoria, strain 85-10<br>*X. axonopodis* pathovar citri, strain 306<br>*X. campestris* pathovar campestris, strain B100<br>*X. oryzae* pathovar oryzae, strain MAFF 311018	2809	0
10	*X. oryzae* pathovar oryzae, strain PXO99A<br>*X. campestris* campestris, strain ATCC 33913<br>*X. oryzae* oryzae, strain KXO85 / KACC10331<br>*X. campestris* pathovar campestris, strain B100	2739	0
11	*X. oryzae* pathovar oryzae, strain PXO99A<br>*X. campestris* pathovar campestris, strain 8004<br>*X. campestris* campestris, strain ATCC 33913<br>*X. oryzae* pathovar oryzae, strain MAFF 311018	2827	0
12	*X. campestris* pathovar campestris, strain 8004<br>*X. campestris* campestris, strain ATCC 33913<br>*X. axonopodis* pathovar citri, strain 306<br>*X. campestris* pathovar campestris, strain B100	3329	40

13	*X. oryzae* pathovar oryzae, strain PXO99A<br>*X. campestris* campestris, strain ATCC 33913<br>*X. campestris* pathovar campestris, strain 8004<br>*X. campestris* pathovar vesicatoria, strain 85-10	2811	0
14	*X. campestris* pathovar campestris, strain 8004<br>*X. campestris* campestris, strain ATCC 33913<br>*X. campestris* pathovar vesicatoria, strain 85-10<br>*X. oryzae* oryzae, strain KXO85 / KACC10331	2780	0
15	*X. oryzae* pathovar oryzae, strain PXO99A<br>*X. campestris* campestris, strain ATCC 33913<br>*X. oryzae* oryzae, strain KXO85 / KACC10331<br>*X. oryzae* pathovar oryzae, strain MAFF 311018	2785	14
16	*X. campestris* pathovar vesicatoria, strain 85-10<br>*X. axonopodis* pathovar citri, strain 306<br>*X. oryzae* oryzae, strain KXO85 / KACC10331<br>*X. campestris* pathovar campestris, strain B100	2737	0
17	*X. campestris* campestris, strain ATCC 33913<br>*X. campestris* pathovar vesicatoria, strain 85-10<br>*X. oryzae* oryzae, strain KXO85 / KACC10331<br>*X. oryzae* pathovar oryzae, strain MAFF 311018	2766	0
18	*X. oryzae* pathovar oryzae, strain PXO99A<br>*X. campestris* campestris, strain ATCC 33913<br>*X. campestris* pathovar campestris, strain B100<br>*X. oryzae* pathovar oryzae, strain MAFF 311018	2804	0
19	*X. campestris* pathovar vesicatoria, strain 85-10<br>*X. oryzae* oryzae, strain KXO85 / KACC10331<br>*X. campestris* pathovar campestris, strain B100<br>*X. oryzae* pathovar oryzae, strain MAFF 311018	2751	0
20	*X. campestris* campestris, strain ATCC 33913<br>*X. campestris* pathovar vesicatoria, strain 85-10<br>*X. campestris* pathovar campestris, strain B100<br>*X. oryzae* pathovar oryzae, strain MAFF 311018	2835	0
21	*X. oryzae* pathovar oryzae, strain PXO99A<br>*X. campestris* pathovar vesicatoria, strain 85-10<br>*X. oryzae* oryzae, strain KXO85 / KACC10331<br>*X. oryzae* pathovar oryzae, strain MAFF 311018	2850	29
22	*X. oryzae* pathovar oryzae, strain PXO99A<br>*X. campestris* campestris, strain ATCC 33913<br>*X. campestris* pathovar vesicatoria, strain 85-10<br>*X. oryzae* oryzae, strain KXO85 / KACC10331	2733	0
23	*X. campestris* campestris, strain ATCC 33913<br>*X. axonopodis* pathovar citri, strain 306<br>*X. oryzae* oryzae, strain KXO85 / KACC10331<br>*X. oryzae* pathovar oryzae, strain MAFF 311018	2753	1
24	*X. oryzae* pathovar oryzae, strain PXO99A<br>*X. campestris* pathovar campestris, strain 8004<br>*X. axonopodis* pathovar citri, strain 306<br>*X. oryzae* oryzae, strain KXO85 / KACC10331	2700	0

25	*X. campestris* pathovar campestris, strain 8004<br>*X. campestris* campestris, strain ATCC 33913<br>*X. axonopodis* pathovar citri, strain 306<br>*X. oryzae* oryzae, strain KXO85 / KACC10331	2750	0
