# Supplementary material for: Analysis and comparison of the pan-genomic properties of sixteen well-characterized bacterial genera
Source: BMC Microbiol. 2010 Oct 13;10:258. doi: 10.1186/1471-2180-10-258 (PMC3020658; doi:10.1186/1471-2180-10-258)
Supplement: Additional file 5 — Complete list of random groups. These tables list the random groups used for the analysis whose results are summarized in Tables 3 and 4 of the main paper. The column heading NC indicates the number of proteins in that group's core proteome, while NU indicates the number of proteins found in the proteomes of all members of that group, but no other isolates from the same genus. [file 1471-2180-10-258-S5.ZIP › Yersinia_4_isolates.pdf]

Random groups corresponding to *Yersinia* species with 4 isolates.

#	Members of random group	N_C	N_U
1	*Y. pseudotuberculosis* serovar O:3, strain YPIII<br>*Y. pestis* biovar Orientalis, strain CO-92<br>*Y. pestis* bv. Antiqua, strain Nepal516<br>*Y. pestis* biovar Mediaevalis, strain KIM5	3256	0
2	*Y. pestis* Pestoides F<br>*Y. pestis* biovar Orientalis, strain CO-92<br>*Y. pseudotuberculosis* serovar IB, strain PB1/+<br>*Y. pestis* bv., strain Antiqua	3322	0
3	*Y. pseudotuberculosis* serovar I, strain IP32953<br>*Y. pseudotuberculosis* serovar IB, strain PB1/+<br>*Y. pseudotuberculosis* serovar O:1b, strain IP 31758<br>*Y. pestis* bv., strain Antiqua	3390	0
4	*Y. pseudotuberculosis* serovar I, strain IP32953<br>*Y. pseudotuberculosis* serovar O:3, strain YPIII<br>*Y. enterocolitica* serovar O:8, strain 8081<br>*Y. pestis* biovar Mediaevalis, strain KIM5	2681	0
5	*Y. pestis* bv. Antiqua, strain Angola<br>*Y. pseudotuberculosis* serovar I, strain IP32953<br>*Y. pestis* biovar Mediaevalis, strain 91001<br>*Y. enterocolitica* serovar O:8, strain 8081	2612	0
6	*Y. pestis* biovar Orientalis, strain CO-92<br>*Y. enterocolitica* serovar O:8, strain 8081<br>*Y. pestis* bv. Antiqua, strain Nepal516<br>*Y. pseudotuberculosis* serovar IB, strain PB1/+	2634	0
7	*Y. pseudotuberculosis* serovar O:3, strain YPIII<br>*Y. pestis* biovar Orientalis, strain CO-92<br>*Y. enterocolitica* serovar O:8, strain 8081<br>*Y. pestis* biovar Mediaevalis, strain KIM5	2653	0
8	*Y. pestis* bv. Antiqua, strain Angola<br>*Y. pseudotuberculosis* serovar IB, strain PB1/+<br>*Y. pseudotuberculosis* serovar O:1b, strain IP 31758<br>*Y. pestis* biovar Mediaevalis, strain KIM5	3067	0
9	*Y. pestis* Pestoides F<br>*Y. pseudotuberculosis* serovar O:3, strain YPIII<br>*Y. pestis* biovar Mediaevalis, strain KIM5<br>*Y. pestis* bv., strain Antiqua	3286	0
10	*Y. pestis* biovar Mediaevalis, strain 91001<br>*Y. enterocolitica* serovar O:8, strain 8081<br>*Y. pseudotuberculosis* serovar IB, strain PB1/+<br>*Y. pseudotuberculosis* serovar O:1b, strain IP 31758	2680	0
11	*Y. enterocolitica* serovar O:8, strain 8081<br>*Y. pestis* biovar Orientalis, strain CO-92<br>*Y. pestis* biovar Mediaevalis, strain KIM5<br>*Y. pestis* bv., strain Antiqua	2670	0
12	*Y. pestis* bv. Antiqua, strain Angola<br>*Y. pestis* biovar Mediaevalis, strain 91001<br>*Y. pseudotuberculosis* serovar IB, strain PB1/+<br>*Y. pestis* bv., strain Antiqua	3145	0

13	*Y. pestis* bv. Antiqua, strain Angola<br>*Y. pseudotuberculosis* serovar O:3, strain YPIII<br>*Y. pseudotuberculosis* serovar O:1b, strain IP 31758<br>*Y. pestis* biovar Mediaevalis, strain KIM5	3066	0
14	*Y. pestis* Pestoides F<br>*Y. pestis* biovar Mediaevalis, strain 91001<br>*Y. pseudotuberculosis* serovar O:3, strain YPIII<br>*Y. pestis* bv., strain Antiqua	3260	0
15	*Y. pseudotuberculosis* serovar I, strain IP32953<br>*Y. pestis* biovar Mediaevalis, strain 91001<br>*Y. pseudotuberculosis* serovar IB, strain PB1/+<br>*Y. pestis* biovar Mediaevalis, strain KIM5	3286	0
16	*Y. pestis* bv. Antiqua, strain Angola<br>*Y. pestis* Pestoides F<br>*Y. pseudotuberculosis* serovar O:3, strain YPIII<br>*Y. pseudotuberculosis* serovar IB, strain PB1/+	3071	0
17	*Y. pseudotuberculosis* serovar I, strain IP32953<br>*Y. pestis* bv. Antiqua, strain Angola<br>*Y. pestis* biovar Orientalis, strain CO-92<br>*Y. pseudotuberculosis* serovar IB, strain PB1/+	3131	0
18	*Y. pseudotuberculosis* serovar O:3, strain YPIII<br>*Y. pestis* biovar Orientalis, strain CO-92<br>*Y. pestis* bv. Antiqua, strain Nepal516<br>*Y. pseudotuberculosis* serovar O:1b, strain IP 31758	3250	0
19	*Y. pseudotuberculosis* serovar I, strain IP32953<br>*Y. pseudotuberculosis* serovar IB, strain PB1/+<br>*Y. pseudotuberculosis* serovar O:1b, strain IP 31758<br>*Y. pestis* biovar Mediaevalis, strain KIM5	3303	0
20	*Y. pestis* bv. Antiqua, strain Angola<br>*Y. pseudotuberculosis* serovar IB, strain PB1/+<br>*Y. pseudotuberculosis* serovar O:1b, strain IP 31758<br>*Y. pestis* bv., strain Antiqua	3106	0
21	*Y. pseudotuberculosis* serovar O:3, strain YPIII<br>*Y. pestis* biovar Orientalis, strain CO-92<br>*Y. pseudotuberculosis* serovar O:1b, strain IP 31758<br>*Y. pestis* bv., strain Antiqua	3289	0
22	*Y. pestis* bv. Antiqua, strain Angola<br>*Y. pestis* Pestoides F<br>*Y. enterocolitica* serovar O:8, strain 8081<br>*Y. pestis* biovar Mediaevalis, strain KIM5	2556	1
23	*Y. pestis* bv. Antiqua, strain Angola<br>*Y. pestis* biovar Orientalis, strain CO-92<br>*Y. pseudotuberculosis* serovar O:1b, strain IP 31758<br>*Y. pseudotuberculosis* serovar IB, strain PB1/+	3059	0
24	*Y. pseudotuberculosis* serovar I, strain IP32953<br>*Y. pestis* Pestoides F<br>*Y. pseudotuberculosis* serovar O:3, strain YPIII<br>*Y. enterocolitica* serovar O:8, strain 8081	2700	0

25	*Y. pestis* bv. Antiqua, strain Angola<br>*Y. pestis* Pestoides F<br>*Y. enterocolitica* serovar O:8, strain 8081<br>*Y. pseudotuberculosis* serovar IB, strain PB1/+	2606	0
