# Supplementary material for: Analysis and comparison of the pan-genomic properties of sixteen well-characterized bacterial genera
Source: BMC Microbiol. 2010 Oct 13;10:258. doi: 10.1186/1471-2180-10-258 (PMC3020658; doi:10.1186/1471-2180-10-258)
Supplement: Additional file 5 — Complete list of random groups. These tables list the random groups used for the analysis whose results are summarized in Tables 3 and 4 of the main paper. The column heading NC indicates the number of proteins in that group's core proteome, while NU indicates the number of proteins found in the proteomes of all members of that group, but no other isolates from the same genus. [file 1471-2180-10-258-S5.ZIP › Yersinia_7_isolates.pdf]

Random groups corresponding to *Yersinia* species with 7 isolates.

#	Members of random group	N_C	N_U
1	*Y. pestis* bv. Antiqua, strain Angola<br>*Y. pseudotuberculosis* serovar O:3, strain YPIII<br>*Y. pestis* biovar Mediaevalis, strain 91001<br>*Y. pestis* biovar Orientalis, strain CO-92<br>*Y. pestis* bv. Antiqua, strain Nepal516<br>*Y. pseudotuberculosis* serovar O:1b, strain IP 31758<br>*Y. pestis* bv., strain Antiqua	2965	0
2	*Y. pseudotuberculosis* serovar I, strain IP32953<br>*Y. pestis* biovar Mediaevalis, strain 91001<br>*Y. enterocolitica* serovar O:8, strain 8081<br>*Y. pseudotuberculosis* serovar IB, strain PB1/+<br>*Y. pseudotuberculosis* serovar O:1b, strain IP 31758<br>*Y. pestis* biovar Mediaevalis, strain KIM5<br>*Y. pestis* bv., strain Antiqua	2625	0
3	*Y. pestis* bv. Antiqua, strain Angola<br>*Y. pestis* biovar Mediaevalis, strain 91001<br>*Y. pseudotuberculosis* serovar O:3, strain YPIII<br>*Y. pestis* biovar Orientalis, strain CO-92<br>*Y. enterocolitica* serovar O:8, strain 8081<br>*Y. pseudotuberculosis* serovar IB, strain PB1/+<br>*Y. pestis* bv., strain Antiqua	2519	0
4	*Y. pestis* bv. Antiqua, strain Angola<br>*Y. pseudotuberculosis* serovar O:3, strain YPIII<br>*Y. pestis* Pestoides F<br>*Y. enterocolitica* serovar O:8, strain 8081<br>*Y. pestis* bv. Antiqua, strain Nepal516<br>*Y. pestis* biovar Mediaevalis, strain KIM5<br>*Y. pestis* bv., strain Antiqua	2494	0
5	*Y. pseudotuberculosis* serovar I, strain IP32953<br>*Y. pestis* bv. Antiqua, strain Angola<br>*Y. pseudotuberculosis* serovar O:3, strain YPIII<br>*Y. enterocolitica* serovar O:8, strain 8081<br>*Y. pestis* bv. Antiqua, strain Nepal516<br>*Y. pseudotuberculosis* serovar IB, strain PB1/+<br>*Y. pestis* bv., strain Antiqua	2530	0
6	*Y. pestis* biovar Mediaevalis, strain 91001<br>*Y. pseudotuberculosis* serovar O:3, strain YPIII<br>*Y. enterocolitica* serovar O:8, strain 8081<br>*Y. pestis* bv. Antiqua, strain Nepal516<br>*Y. pseudotuberculosis* serovar O:1b, strain IP 31758<br>*Y. pestis* biovar Mediaevalis, strain KIM5<br>*Y. pestis* bv., strain Antiqua	2600	0
7	*Y. pestis* biovar Mediaevalis, strain 91001<br>*Y. pestis* Pestoides F<br>*Y. pestis* biovar Orientalis, strain CO-92<br>*Y. enterocolitica* serovar O:8, strain 8081<br>*Y. pestis* bv. Antiqua, strain Nepal516<br>*Y. pseudotuberculosis* serovar O:1b, strain IP 31758<br>*Y. pseudotuberculosis* serovar IB, strain PB1/+	2565	0

8	*Y. pseudotuberculosis* serovar I, strain IP32953<br>*Y. pestis* bv. Antiqua, strain Angola<br>*Y. pseudotuberculosis* serovar O:3, strain YPIII<br>*Y. pestis* Pestoides F<br>*Y. pseudotuberculosis* serovar IB, strain PB1/+<br>*Y. pestis* biovar Mediaevalis, strain KIM5<br>*Y. pestis* bv., strain Antiqua	2989	0
9	*Y. pestis* bv. Antiqua, strain Angola<br>*Y. pestis* biovar Mediaevalis, strain 91001<br>*Y. pseudotuberculosis* serovar O:3, strain YPIII<br>*Y. pestis* Pestoides F<br>*Y. enterocolitica* serovar O:8, strain 8081<br>*Y. pestis* biovar Orientalis, strain CO-92<br>*Y. pseudotuberculosis* serovar O:1b, strain IP 31758	2497	0
10	*Y. pestis* bv. Antiqua, strain Angola<br>*Y. pestis* biovar Orientalis, strain CO-92<br>*Y. enterocolitica* serovar O:8, strain 8081<br>*Y. pseudotuberculosis* serovar O:1b, strain IP 31758<br>*Y. pseudotuberculosis* serovar IB, strain PB1/+<br>*Y. pestis* biovar Mediaevalis, strain KIM5<br>*Y. pestis* bv., strain Antiqua	2512	0
11	*Y. pestis* bv. Antiqua, strain Angola<br>*Y. pseudotuberculosis* serovar I, strain IP32953<br>*Y. pseudotuberculosis* serovar O:3, strain YPIII<br>*Y. pestis* biovar Mediaevalis, strain 91001<br>*Y. enterocolitica* serovar O:8, strain 8081<br>*Y. pseudotuberculosis* serovar IB, strain PB1/+<br>*Y. pestis* bv., strain Antiqua	2540	0
12	*Y. pseudotuberculosis* serovar I, strain IP32953<br>*Y. pestis* bv. Antiqua, strain Angola<br>*Y. pestis* biovar Mediaevalis, strain 91001<br>*Y. pseudotuberculosis* serovar O:3, strain YPIII<br>*Y. pestis* Pestoides F<br>*Y. enterocolitica* serovar O:8, strain 8081<br>*Y. pestis* bv., strain Antiqua	2521	0
13	*Y. pestis* bv. Antiqua, strain Angola<br>*Y. pseudotuberculosis* serovar I, strain IP32953<br>*Y. pestis* biovar Mediaevalis, strain 91001<br>*Y. pestis* bv. Antiqua, strain Nepal516<br>*Y. pseudotuberculosis* serovar IB, strain PB1/+<br>*Y. pseudotuberculosis* serovar O:1b, strain IP 31758<br>*Y. pestis* bv., strain Antiqua	2980	0
14	*Y. pestis* bv. Antiqua, strain Angola<br>*Y. pseudotuberculosis* serovar I, strain IP32953<br>*Y. pseudotuberculosis* serovar O:3, strain YPIII<br>*Y. enterocolitica* serovar O:8, strain 8081<br>*Y. pestis* biovar Orientalis, strain CO-92<br>*Y. pseudotuberculosis* serovar IB, strain PB1/+<br>*Y. pestis* bv., strain Antiqua	2529	0

15	*Y. pseudotuberculosis* serovar O:3, strain YPIII<br>*Y. pestis* biovar Mediaevalis, strain 91001<br>*Y. pestis* Pestoides F<br>*Y. pestis* biovar Orientalis, strain CO-92<br>*Y. enterocolitica* serovar O:8, strain 8081<br>*Y. pestis* bv. Antiqua, strain Nepal516<br>*Y. pestis* biovar Mediaevalis, strain KIM5	2559	0
16	*Y. pseudotuberculosis* serovar I, strain IP32953<br>*Y. pestis* biovar Mediaevalis, strain 91001<br>*Y. pseudotuberculosis* serovar O:3, strain YPIII<br>*Y. pestis* biovar Orientalis, strain CO-92<br>*Y. pseudotuberculosis* serovar IB, strain PB1/+<br>*Y. pseudotuberculosis* serovar O:1b, strain IP 31758<br>*Y. pestis* biovar Mediaevalis, strain KIM5	3176	1
17	*Y. pseudotuberculosis* serovar I, strain IP32953<br>*Y. pestis* biovar Orientalis, strain CO-92<br>*Y. enterocolitica* serovar O:8, strain 8081<br>*Y. pestis* bv. Antiqua, strain Nepal516<br>*Y. pseudotuberculosis* serovar IB, strain PB1/+<br>*Y. pseudotuberculosis* serovar O:1b, strain IP 31758<br>*Y. pestis* biovar Mediaevalis, strain KIM5	2593	0
18	*Y. pestis* bv. Antiqua, strain Angola<br>*Y. pseudotuberculosis* serovar O:3, strain YPIII<br>*Y. pestis* Pestoides F<br>*Y. pestis* biovar Orientalis, strain CO-92<br>*Y. pestis* bv. Antiqua, strain Nepal516<br>*Y. pseudotuberculosis* serovar IB, strain PB1/+<br>*Y. pestis* bv., strain Antiqua	2958	0
19	*Y. pseudotuberculosis* serovar O:3, strain YPIII<br>*Y. pestis* biovar Mediaevalis, strain 91001<br>*Y. pestis* biovar Orientalis, strain CO-92<br>*Y. pseudotuberculosis* serovar IB, strain PB1/+<br>*Y. pseudotuberculosis* serovar O:1b, strain IP 31758<br>*Y. pestis* biovar Mediaevalis, strain KIM5<br>*Y. pestis* bv., strain Antiqua	3184	0
20	*Y. pestis* biovar Mediaevalis, strain 91001<br>*Y. pestis* Pestoides F<br>*Y. pestis* biovar Orientalis, strain CO-92<br>*Y. enterocolitica* serovar O:8, strain 8081<br>*Y. pseudotuberculosis* serovar IB, strain PB1/+<br>*Y. pestis* biovar Mediaevalis, strain KIM5<br>*Y. pestis* bv., strain Antiqua	2588	0
21	*Y. pseudotuberculosis* serovar I, strain IP32953<br>*Y. pseudotuberculosis* serovar O:3, strain YPIII<br>*Y. pestis* Pestoides F<br>*Y. pestis* biovar Orientalis, strain CO-92<br>*Y. enterocolitica* serovar O:8, strain 8081<br>*Y. pseudotuberculosis* serovar O:1b, strain IP 31758<br>*Y. pestis* bv., strain Antiqua	2607	0

22	*Y. pestis* bv. Antiqua, strain Angola<br>*Y. pestis* biovar Mediaevalis, strain 91001<br>*Y. pestis* Pestoides F<br>*Y. pseudotuberculosis* serovar O:1b, strain IP 31758<br>*Y. pseudotuberculosis* serovar IB, strain PB1/+<br>*Y. pestis* biovar Mediaevalis, strain KIM5<br>*Y. pestis* bv., strain Antiqua	2977	0
23	*Y. pestis* bv. Antiqua, strain Angola<br>*Y. pestis* Pestoides F<br>*Y. pestis* biovar Orientalis, strain CO-92<br>*Y. pestis* bv. Antiqua, strain Nepal516<br>*Y. pseudotuberculosis* serovar O:1b, strain IP 31758<br>*Y. pseudotuberculosis* serovar IB, strain PB1/+<br>*Y. pestis* biovar Mediaevalis, strain KIM5	2940	0
24	*Y. pestis* bv. Antiqua, strain Angola<br>*Y. pseudotuberculosis* serovar I, strain IP32953<br>*Y. pestis* Pestoides F<br>*Y. pestis* biovar Mediaevalis, strain 91001<br>*Y. pseudotuberculosis* serovar O:3, strain YPIII<br>*Y. enterocolitica* serovar O:8, strain 8081<br>*Y. pestis* biovar Mediaevalis, strain KIM5	2496	0
25	*Y. pestis* bv. Antiqua, strain Angola<br>*Y. pseudotuberculosis* serovar I, strain IP32953<br>*Y. pseudotuberculosis* serovar O:3, strain YPIII<br>*Y. pestis* biovar Mediaevalis, strain 91001<br>*Y. pseudotuberculosis* serovar O:1b, strain IP 31758<br>*Y. pestis* biovar Mediaevalis, strain KIM5<br>*Y. pestis* bv., strain Antiqua	2992	0
